# Spinal cord structural and functional architecture and its shared organization with the brain across the adult lifespan

**DOI:** 10.1038/s41467-026-71963-2

**Published:** 2026-04-16

**Authors:** Caroline Landelle, Nawal Kinany, Samuelle St-Onge, Ovidiu Lungu, Dimitri Van De Ville, Bratislav Misic, Véronique Marchand-Pauvert, Benjamin De Leener, Julien Doyon

**Affiliations:** 1 McConnell Brain Imaging Centre, Department of Neurology and Neurosurgery, Montreal Neurological Institute, McGill University, Montreal, QC Canada; 2 Department of Radiology and Medical Informatics, University of Geneva, Geneva, Switzerland; 3 Neuro-X Institute, Ecole Polytechnique Fédérale de Lausanne (EPFL), Geneva, Switzerland; 4 NeuroPoly Lab, Institute of Biomedical Engineering, Polytechnique Montreal, Montreal, QC Canada; 5 CHU Sainte-Justine Research Centre, Montreal, QC Canada; 6 Centre de recherche de l’Institut Universitaire de Gériatrie de Montréal, Centre de Recherche de l’Institut Universitaire en Santé Mentale de Montréal et Département de Psychiatrie et Addictologie, Université de Montréal, Montréal, QC Canada; 7 Sorbonne Université, Inserm, CNRS, Laboratoire d’Imagerie Biomédicale, LIB, Paris, France; 8 Department of Computer Engineering and Software Engineering, Polytechnique Montreal, Montreal, QC Canada

**Keywords:** Neural ageing, Spinal cord, Sensory processing

## Abstract

The spinal cord connects the brain to peripheral systems. Yet its integration with cerebral networks remains a key neuroscience question. Capturing structural and functional central nervous system (CNS) changes throughout the lifespan is essential for characterizing healthy and pathological aging. Leveraging a unique multimodal dataset combining spinal and cerebrospinal imaging, we jointly mapped the spinal cord structural and functional architecture across adulthood. Our results revealed age-related changes across these modalities and identified organizational principles shared with the brain. These changes were most pronounced in the somatosensory pathway, with microstructural decline coupled to shifts in functional connectivity and local spontaneous activity as aging progresses. Extending analyses to the brain uncovered convergent CNS-wide aging mechanisms, including gray matter loss, decreased functional segregation, and increased spontaneous activity, highlighting shared neural aging trajectories. Together, our findings provide a systems-level view of the age-related alteration in the CNS, offering a foundation for future studies investigating potential imaging markers of sensorimotor dysfunction.

## Abstract (web summary)

Using multimodal spinal cord and brain imaging, this study reveals pronounced age-related changes in the spinal somatosensory pathway and shared central nervous system aging mechanisms, including gray matter loss and decreased functional segregation.

## Introduction

Mapping the brain connectome is a core pursuit in neuroscience aimed at understanding how the anatomical architecture shapes functional interactions and how these evolve across the lifespan. Key findings indicate that brain microstructural and functional features follow a sensory-to-associative hierarchy^1–4^, characterized by stronger coupling in sensory and motor cortices compared to associative areas. This hierarchical organization reflects a functional organization from perception and action to integration^5^, which evolves across the lifespan and in diseases^6–8^. However, these investigations are limited to the brain and overlook the spinal cord, a critical component of the central nervous system (CNS) that relays and processes somatomotor information between the brain and the periphery. This gap raises important questions: does the spinal cord exhibit properties similar to those of the cortical sensory and motor areas, such as strong similarities among functionally related spinal segments? How does this organization evolve across the lifespan? Mapping the healthy structural and functional architecture of the spinal cord is thus crucial for detecting deviations that may underlie pathological aging and neurological disorders.

Recent neuroimaging advances have provided unique opportunities to assess both functional and structural properties of the spinal cord in vivo^9–11^, and to image the brain and spinal cord simultaneously^12,13^. Concomitant advances in spinal cord-specific analysis pipelines have enabled direct functional connectivity mapping between multiple spinal segments in both health and disease^14–19^ and unveiled the large-scale cerebrospinal topography of the somatomotor pathways in vivo^20–22^. Altogether, these advances have made it possible to deepen our understanding of somatomotor pathway architecture, from the cortex down to the spinal cord.

Importantly, the neural bases that support somatosensory and motor function undergo significant changes across the lifespan, resulting in altered sensorimotor performances with aging^23–25^. Elucidating how aging affects the different CNS structures is thus essential for delineating healthy aging trajectories, identifying periods of heightened vulnerability to sensorimotor disorders such as Parkinson’s disease, and predicting how aging progresses in individuals with spinal cord conditions. Although substantial research has explored these changes at the brain level^11,26,27^, few studies have focused on the spinal cord’s involvement. Notably, existing literature on the aging spinal cord has mainly investigated structure and function separately. Structural investigations have primarily reported age-related demyelination or reductions in cross-sectional area using neuroimaging techniques^28–31^, while functional changes, such as hyperactivity, have been inferred from electrophysiological studies in non-human models^32–34^. To date, however, no study has directly linked age-related structural and functional changes along the cervical spinal cord in vivo, nor examined how these changes reflect those observed at the brain level, leaving a critical gap in our understanding of spinal cord aging and its contribution to sensorimotor decline.

In this study, we acquired a unique and comprehensive adult lifespan neuroimaging dataset (participants from 20 to 80 years) to characterize cervical spinal cord microstructure and simultaneously brain and spinal cord function in vivo. Using multimodal spinal MRI, we examine spinal architecture, structure-function coupling, and age-related alterations. We expected that due to the high specificity of sensorimotor processing^2^, regions within the same spinal segments would share similar functional and structural characteristics. We further hypothesized that aging would be associated with spinal cord microstructural atrophy^28^ and functional changes^33,35^, which would manifest differently in the dorsal and ventral pathways due to their distinct roles in somatosensory and motor processing, respectively, as well as their cellular composition. Building on this expectation of region-specific changes and extensive literature on the aging brain^36^, we also predicted that functional age-related changes in the spinal cord would be linked to structural alterations. Lastly, we conducted structural and functional MRI, simultaneously for the brain and spinal cord, enabling us to extend our understanding of the hierarchical organization from the cortex down to the spinal cord, and to investigate the age-related changes across CNS levels. We then hypothesized that structural and functional changes in the spinal cord, including atrophy, decreased functional segregation, and alterations in local dynamics, parallel those observed in the brain, hence supporting the existence of shared CNS-wide aging trajectories.

## Results

This cross-sectional study included three microstructural spinal cord imaging contrasts: T2*-weighted (T2s), magnetization transfer (MT) and diffusion-weighted imaging (DWI), as well as structural (T1w: T1-weighted) and functional MRI volumes covering simultaneously the brain and cervical spinal cord (from the top of the brain to T1 vertebral level) in 70 healthy right-handed participants aged 20–80 years old (36 females, Supplementary Figs. S1, S2A, and Table S1). Data quality was systematically assessed across modalities using visual inspection of all images, as well as quantitative measures including framewise displacement (FD, Supplementary Fig. S2B), and temporal signal-to-noise ratio (tSNR; Supplementary Fig. S2C) of functional images. Three participants were excluded due to excessive motion (FD > 0.3 mm). When testing for age effects, FD showed a significant increase with age at the brain level (*t*(65) = 4.75, *P* < 0.001) but not at the spinal cord level (*t*(65) = 0.34, *P* = 0.74). Functional data quality was further evaluated using tSNR, which showed no significant age-related effects in either the spinal cord (mean ± SD: 22.16 ± 2.0; *P* = 0.095) or the brain (58.55 ± 4.9; *P* = 0.22). Exclusions based on structural scans were due to modality-specific artifacts (*n* = 15) or incomplete acquisitions (*n* = 1). After quality control, the final sample comprised 67 participants with T2s, T1w, and fMRI imaging. Among these, 65 participants 55 also had MT and 55 DWI data.

**Fig. 1:**
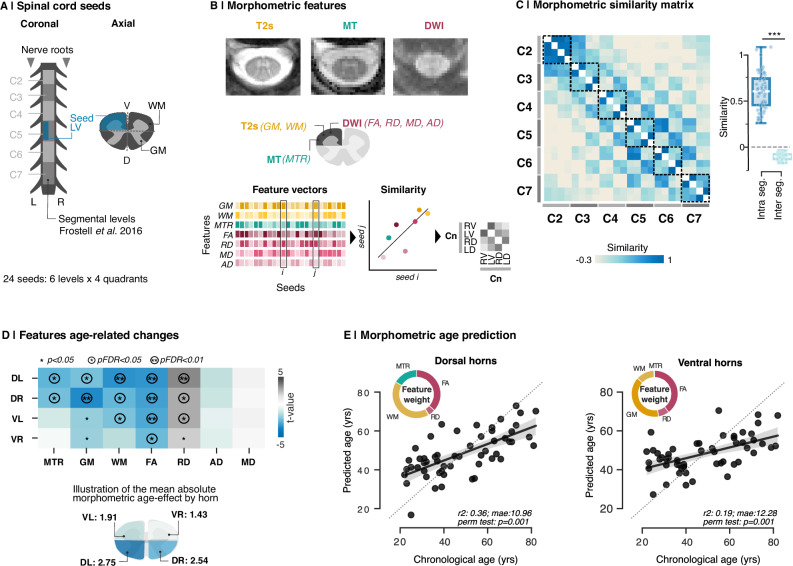
Age-related changes of the spinal cord microstructure. **A** Spinal cord images were parcellated into 24 regions [C2 to C7 Frostell segments subdivided into four horns: dorsal-right (DR), dorsal-left (DL), ventral-right (VR), and ventral-left (VL)]. **B** Seven microstructural features were extracted for each spinal cord region (*n* = 24) from T2s contrast (GM gray matter voxel counts, WM white matter voxel counts), the magnetization transfer (MTR magnetization transfer ratio), and the diffusion (DWI) image (FA fractional anisotropy, RD radial diffusivity, MD mean diffusivity, AD axial diffusivity). Each region is represented as a seven-feature vector. Morphometric similarity was then computed between the feature vectors of every pair of regions using correlations. The resulting correlation coefficients were compiled into a participant-specific 24x24 morphometric similarity matrix. **C** Group-average morphometric similarity matrix. Intra- and inter-segments similarity were compared using a two-tailed paired *t* test (***P = 4.06e-29, *n* = 54). Boxplots show intra- (left) and inter-segments (right) similarity. Each box represents the distribution (i.e., from the 25th to the 75th percentile), whereas the medians are represented by the horizontal white line inside the box. Vertical extending lines denote the extreme values within a 1.5 interquartile range. Each dot is an individual participant. **D** The heatmap represents *t* values reflecting the age-related effect on each microstructural feature derived from linear mixed models, within participant as a random effect (blue indicates a decrease with age, gray an increase). The significance of each fixed effect was assessed using a two-sided *t* test. The mean absolute age-effect across features is illustrated on an axial view of the spinal cord for each quadrant, providing a concise overview of how each region is affected across metrics. **E** Microstructural features were used to predict the age of the participants using an elastic net regression model separately for dorsal and ventral regions. The scatter plot shows the relationship between chronological age and model-predicted age, solid line represents the linear regression fit, and the shaded gray area indicated the 95% confidence interval. The significance of the linear fit was assessed using a two-sided *t* test. The donut plot illustrates the percentage of contribution (or weight) of each feature to the prediction model.

Spinal cord analyses were performed using Frostell’s segmental level parcellation^37^, with each segment subdivided into four parcels: dorsal-right (DR), dorsal-left (DL), ventral-right (VR), and ventral-left (VL, Fig. 1A). Each parcel includes both gray and white matter, ensuring comparability between structural and functional analyses while minimizing the partial volume effects between tissue types. To validate Frostell’s segmental organization in our dataset, we employed a data-driven approach to extract functional segments (Supplementary Fig. S3A)^17^. We found a high degree of overlap between the Frostell’s atlas and the extracted components, as evidenced by a Dice coefficient of 0.80 ± 0.03 (Supplementary Fig. S3B).

The results are organized as follows. We first investigated whether different spinal regions exhibit similar microstructural organization and how this organization changes with aging. Second, we studied the spinal cord functional organization and its age-related alterations. Third, we characterized the coupling between spinal structural and functional organization in relation to aging. Finally, we examined whether structural and functional age-related changes in the spinal cord parallel those observed in the brain.

### Aging of the spinal cord microstructure

To characterize the network organization of the spinal cord, we integrated multiple microstructural MRI features that capture distinct anatomical properties across regions. More specifically, we extracted seven microstructural features (Fig. 1B): T2*w-based GM and WM voxel counts, MT ratio (MTR) from MT imaging, and diffusion-derived metrics from DWI (i.e., FA: fractional anisotropy, RD: radial diffusivity, MD: mean diffusivity, AD: axial diffusivity) between C2 and C7 segments. To provide a multivariate representation of spinal cord microstructure, we combined all microstructural features into a single regional profile, allowing us to capture shared microstructural patterns between the different regions that are not evident when individual metrics are examined separately. The morphometric similarity between two spinal cord regions was then defined as the Pearson correlation between their microstructural features, providing an index of how similar regions are in their overall microstructural organization. High morphometric similarity therefore indicates that two regions share comparable microstructural organization across multiple MRI features. Using this approach, we found that regions belonging to the same spinal cord segment exhibited greater similarity than those from other cervical segments (Fig. 1C, paired two-tailed *t* test; *t*(53) = 22.86, *P* < 0.001).

Next, we examined age-related changes in the microstructural organization of each spinal cord parcel to determine whether aging affects the spinal cord uniformly or follows a region-specific pattern. Using a linear mixed model (LMM) including age and sex as fixed effects and participants as random effects, we estimated the linear age-related alterations for each feature across the four spinal quadrants (i.e., DR, DL, VR, VL). Figure 1D provides a comprehensive overview of age effects across all features and quadrants, showing significant age-related decreases for MTR, WM voxels count, GM voxels count and FA, alongside an increase in RD (Fig. 1D and Supplementary Table S3). The greatest changes were found in the dorsal-left (DL) horn (MTR: p_FDR_ = 0.011, WM: p_FDR_ = 0.0012, GM: p_FDR_ = 0.019, FA: p_FDR_ < 0.0013, RD: p_FDR_ < 0.0031). To assess the stability of these estimates, we performed bootstrap resampling (1000 iterations), and the resulting 95% confidence intervals are reported in Supplementary Table S3. Averaging the absolute *t* values across features confirmed that microstructural changes were more pronounced in the DL horn (*t *= 2.71 ± 1.12), followed by DR (*t *= 2.54 ± 1.17), VL (*t* = 1.91 ± 0.97), and VR (*t* = 1.44 ± 0.9) horns. To further visualize age-related patterns, we generated average metric maps for different age subgroups (Supplementary Figs. S4 and S5). Note that sex effects are also reported in supplementary materials (Supplementary Table S3) and show an increase in RD, GM, and WM voxel counts and a decrease in FA in males compared to females.

To confirm whether the spinal cord microstructural aging follows a region-specific, distinctly affecting the dorsal (somatosensory pathway) and ventral hemicord (motor pathway), we employed two elastic net regression models incorporating all extracted features to predict participants’ age. Microstructural features predicted age more accurately in the dorsal spinal cord (Mean absolute error, MAE: 10.96 years, *r*^2^ = 0.36) than in the ventral regions (MAE: 12.28 years, *r*^2^ = 0.19, Fig. 1E). Permutation testing (1000 iterations with shuffled age labels) confirmed that both models performed significantly above chance (dorsal: p_perm_R² <0.001, p_perm_MAE < 0.001; ventral: p_perm_R² <0.001, p_perm_MAE < 0.001; Supplementary Fig. S7). In the dorsal horn, WM voxel counts and FA contributed most strongly to age prediction (WM voxel counts: 83.24% of the explained variance, FA: 73.21%, MTR: 32.93%, RD: 9.34%; GM voxel counts: 0.33%, AD: 0%, MD: 0%). In the ventral horn, FA and GM voxel counts were the dominant contributors (FA: 57.88%, GM counts: 57.40%, WM counts: 18.64%, RD: 11.07%, MTR: 0.81%, AD: 0%, MD: 0%). These results indicate that the age prediction is not driven by a single metric, but instead reflects complementary metric contributions, with distinct dorsal–ventral profiles.

Lastly, complementary analyses were performed within the three white matter tracts (dorsal column, lateral funiculi, and ventral funiculi) to assess tract-specific microstructural changes in the spinal cord. Results showed that the dorsal column (proprioceptive and tactile fibers) exhibited the strongest age-related effect in MTR, FA, and RD (Supplementary Fig. S6). Lower but significant age-related changes were also observed for the three metrics in the lateral funiculi (mainly motor fibers), whereas in the ventral funiculi (nociceptive and thermoceptive fibers), only FA and RD showed significant changes with aging. Overall, these findings suggest that aging of the spinal cord microstructure is more pronounced in somatosensory ascending pathways than in motor descending pathways.

### Aging effects on the functional topography of the spinal cord

**Fig. 2:**
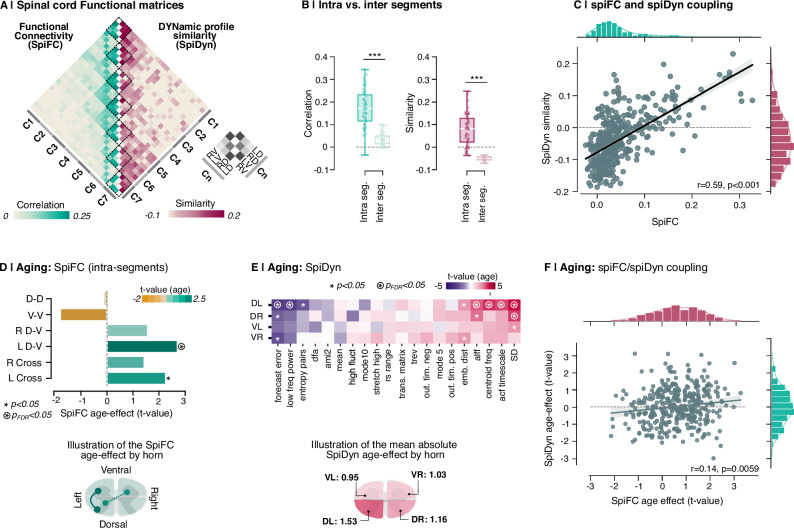
Age-related changes in spinal cord functional topography. **A** Spinal cord functional connectivity (SpiFC, green) and dynamic profile similarity (SpiDyn, pink) matrices were computed using pairwise correlation between all possible spinal regions (28 × 28). **B** The two boxplots show intra- (left) and inter-segments (right) correlation (green) or similarity (pink). Each box represents the distribution (i.e., from the 25th to the 75th percentile), whereas the medians are represented by the horizontal white line inside the box. Vertical extending lines denote the extreme values within a 1.5 interquartile range. Each dot is an individual participant. Intra- and inter-segment SpiFC and SpiDyn values were compared using a two-tailed paired *t* test. Both were significantly higher for region pairs belonging to the same segment (***P = 3.82^e-26^ and *P* = 8.66^e-24^, *n* = 67). **C** SpiFC and SpiDyn coupling values were assessed by correlation between group-average SpiFC connectivity values with SpiDyn similarity values across all spinal region pairs (28 × 27/2 = 378 combinations). Each dot represents a pairwise combination of spinal regions, a linear regression line is overlaid for visualization, and the shaded gray area indicated the 95% confidence interval. *P* = 2.52^e-36^. **D** Age-effects on functional connectivity within spinal segments (intra-segment connectivity) were estimated using linear mixed models (LMMs) for six connection types: dorso-dorsal (D-D), ventro-ventral (V-V), right dorso-ventral (R D-V), left dorso-ventral (L D-V), right cross-horns (R Cross), left cross-horns (L Cross). The significance of each fixed effect was assessed using a two-sided *t* test. Bar plots show the resulting age-effect *t* values for each connection type. Positive *t* values (green) indicate increased connectivity with age, whereas negative *t* values (yellow) indicate decreased connectivity. Significant effects before FDR correction (dotted lines, p_uncorr_ = 0.023, p_FDR_ = 0.069) and after FDR correction (solid lines, p_uncorr_ = 0.0065, p_FDR_ = 0.039 are illustrated on axial spinal cord schematics. **E** Age-effects on SpiDyn features were estimated using LMMs. The heatmap shows *t* values for age effects across SpiDyn feature (violet indicates a decrease with age, pink an increase). The significance of each fixed effect was assessed using a two-sided *t* test. The mean absolute effect across features is illustrated on axial spinal cord schematics. **D**, **E** Significant results before and after FDR correction are marked with an asterisk or a circled asterisk, respectively. **F** To determine whether spinal region pairs showing age-related changes in SpiFC also exhibit corresponding changes in SpiDyn, we computed correlations between *t* values from age-effect models. The scatter plot shows the correlation between age-related effects on SpiDyn and SpiFC across spinal region pairs (*r* denotes the Spearman’s correlation coefficient). Each dot represents one pairwise combination of spinal regions, a linear regression line was overlaid on the scatter plot for visualization, and the shaded gray area indicated the 95% confidence interval. Cn cervical spinal cord level, DL dorsal left, DR dorsal right, VL ventral left, VR ventral right.

To extend our analysis beyond morphometric organization, we characterized the functional architecture of the spinal cord by evaluating distinct functional properties across its regions. As a first step in characterizing spinal cord functional organization, we employed the most widely used fMRI approach for assessing spinal cord function at rest: inter-regional functional connectivity (SpiFC)^15,18,38,39^. This approach estimates the degree of synchronized activity across spinal cord regions by computing pairwise correlations between their time series. Based on the correlation coefficient, we constructed SpiFC matrix across 28 spinal regions (C1 to C7 segments, each divided into four quadrants, Fig. 2A). To capture the intra-regional dynamical signature of spinal cord function beyond inter-regional connectivity, we next analyzed the dynamic profiles of spinal cord activity (SpiDyn) by analyzing the spontaneous temporal dynamic of timeseries fluctuations within each of the 28 regions. Specifically, SpiDyn characterizes the regional signal fluctuation from 20 features: the signal mean, standard deviation, amplitude of low fluctuation frequency (ALFF); a commonly used fMRI biomarker of local spinal activity^40–42^, alongside 17 timeseries features derived from the catch-22 toolbox^43^, which quantify the dynamic properties of the spontaneous fMRI signals, such as signal complexity, temporal predictability and variability. To summarize the similarity of dynamic signature across regions, we constructed a SpiDyn similarity matrix by computing pairwise correlations between the 20-dimensional feature vectors of each spinal region (Fig. 2A). This provides an index of how alike the dynamic profiles are across the spinal cord, analogous to the morphometric similarity approach applied to structural features. Results revealed that spinal regions within the same spinal segment had significantly greater functional connectivity and dynamic profile similarity than those from different segments (paired two-tailed *t* test for SpiFC: *t*(66) = 17.25, *P* < 0.001 and SpiDyn: *t*(66) = 15.52, *P* < 0.001; Fig. 2B). We also found a positive correlation between SpiDyn and SpiFC (Spearman’s *r* = 0.59, *P* < 0.001; Fig. 2C), hence suggesting that spinal regions with similar local dynamics exhibit synchronized spontaneous activity.

To investigate whether aging impacts this spinal cord functional organization, we first estimated age-related changes in SpiFC. The results revealed a significant increase in average inter-segmental connectivity with age (*t*(65) = 2.93, *P* = 0.0033), whereas intra-segmental connectivity showed no significant change (*t*(65) = 0.85, *P* = 0.39). When examining the six specific connection types (i.e., dorso-dorsal, ventro-ventral, left dorso-ventral, right dorso-ventral, left cross-horns, right cross-horns), inter-segmental connectivity significantly increased with age across all connections except the dorso-dorsal (Supplementary Table S4). For intra-segmental connectivity, age-related increases were observed in the left dorso-ventral (*t* = 2.71, *P* = 0.0065, p_FDR_ = 0.039) and left cross-horns connections (*t *= 2.26, *P* = 0.023, p_FDR_ = 0.069), although the latter did not survive correction for multiple comparisons (Fig. 2D and Supplementary Table S5). The increase in inter-segmental connections suggests a decline in the specificity of spinal connectivity with age. In parallel, selective increases in intra-segmental dorso-ventral FC, particularly those driven by the left dorsal horn, likely reflect age-related changes in sensory modulation of motor neurons.

We further examined age-related changes in local spinal cord dynamics (Fig. 2E). The left dorsal horn showed the most pronounced changes, with several SpiDyn features exhibiting a significant increase with aging after multiple corrections (standard deviation, autocorrelation timescale, centroid frequency, and ALFF) or decrease (Forecast error, low-frequency power); Supplementary Table S6). Standard deviation also increased in the right dorsal horn. These patterns suggest that dorsal horns, especially the left side, exhibit age-related changes characterized by increased signal variability and amplitude (i.e., higher standard deviation and ALFF), alongside reduced signal complexity (i.e., lower low-frequency power) and enhanced predictability (i.e., longer autocorrelation timescale and centroid frequency, decreased forecast error).

Finally, we investigated local dynamic features that showed statistically significant age-related changes to investigate whether alterations in inter-regional spiFC, measured independently from SpiDyn, were associated with local dynamic properties evolving with age across spinal regions. Specifically, we quantified the age-related effect for each pairwise connection in the SpiFC matrix and each pairwise similarity in the SpiDyn matrix before assessing their relationship using Spearman correlation. Notably, the results revealed a weak but significant positive correlation (*r* = 0.15, *P* = 0.003), indicating that spinal regions exhibiting age-related changes in functional connectivity also show corresponding age effects in their time-series dynamic similarity profiles. These results suggest a simultaneous alteration in both spontaneous inter-regional connectivity and local intrinsic dynamics with aging at the spinal cord level. Recognizing the weak association is important as it may reflect a combination of shared and distinct neurobiological processes, highlighting the need to assess both functional aspects to comprehensively capture age-related changes. Sex effects were systematically tested and are reported in supplementary material (Supplementary Tables S4–S6). We observed sex differences in SpiFC, with females showing increased intra-segmental connectivity compared to males, whereas SpiDyn analyses revealed higher ALFF values in males than females in the dorsal hemicord. These findings highlight the importance of accounting for sex as a variable in the analysis.

### Aging of the spinal cord structure-function coupling

**Fig. 3:**
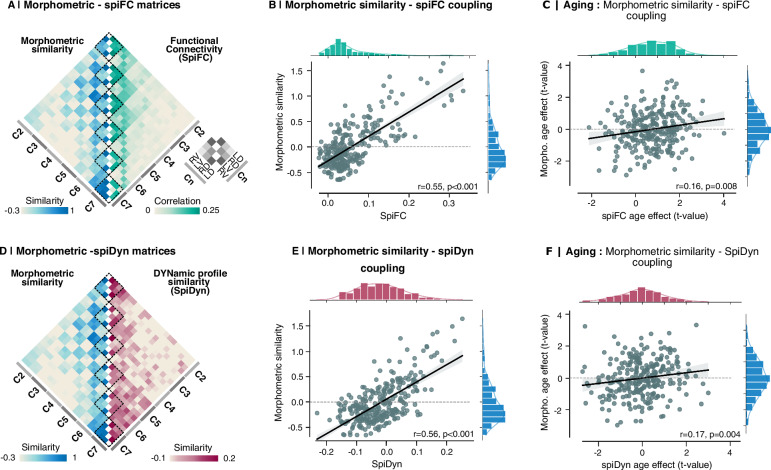
Age-related changes in spinal cord structure–function coupling. **A**, **D** Spinal cord morphometric similarity matrices (blue, **A**, **D**) and spinal cord functional connectivity matrix (SpiFC, green, **A**) or spinal cord dynamic profile similarity matrix (SpiDyn, pink, **D**) were computed using pairwise correlation between all possible spinal regions (24 × 24). **B**, **E** Coupling between group-average morphometric similarity and SpiFC (**B**) or SpiDyn (**E**) was assessed using Spearman correlations across pairwise combinations of spinal regions (24 × 23/2 = 276 combinations). Each dot represents one region–region pair, and *r* denotes Spearman’s correlation coefficient. We found a strong positive correlation between the two matrices (SpiFC: *P* = 2.50^e-23^ and SpiDyn *P* = 3.52^e-24^). **C**, **F** To determine whether spinal region pairs showing age-related changes in morphometric similarity also exhibit corresponding changes in SpiFC or SpiDyn, we computed correlations between *t* values from age-effect models. The scatter plot shows the correlation between age-related effects on morphometric similarity and SpiFC or SpiDyn across spinal region pairs (r denotes the Spearman’s correlation coefficient). **B**, **C**, **E**, **F** Each dot represents a pairwise combination of spinal regions; a linear regression line was overlaid on the scatter plot for visualization, the shaded gray area indicated the 95% confidence interval, and *r* denotes the Spearman coefficient of correlation.

We next aimed to determine whether functional connectivity (SpiFC) and regional activity patterns (SpiDyn) mirror the microstructural architecture of the spinal cord. The results revealed a strong and significant correlation between morphometric similarity and both SpiFC (Spearman’s *r* = 0.55, *P* < 0.001, Fig. 3A, B) and SpiDyn similarity measures (Spearman’s *r* = 0.56, *P* < 0.006, Fig. 3D, E). These findings suggest that the spinal cord organization, encompassing both inter-regional connectivity and local dynamics, may be shaped by its microstructural architecture.

Next, to assess whether aging similarly affects both functional and structural organization of the spinal cord, we estimated how the strength of coupling between microstructural and functional measures (i.e., SpiFC and SpiDyn) is modulated by age. Correlation analyses revealed that microstructural-functional coupling increases with aging, both for inter-regional functional connectivity (Spearman’s *r*: 0.16, *P* = 0.0083, Fig. 3C) and intra-regional dynamic profiles (Spearman’s *r*: 0.17, *P* = 0.004, Fig. 3D).

Altogether, these results highlight a strong coupling between microstructural and functional organization at the spinal cord level, with structural age-related effects being linked to changes in functional connectivity and local dynamics.

### Parallel structural and functional age-related changes of the brain and spinal cord

**Fig. 4:**
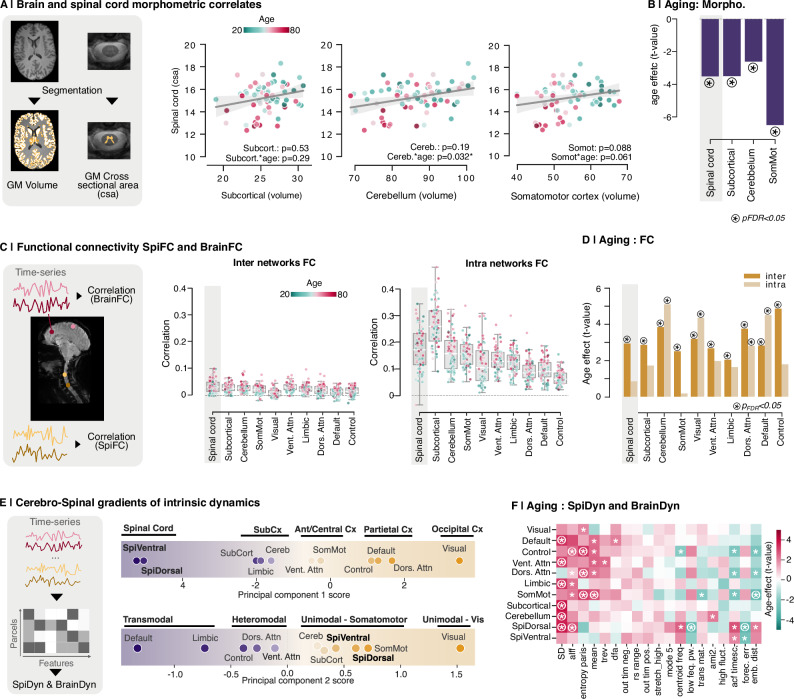
Brain and spinal cord correlates and their age-related changes. **A** Cerebral gray matter (GM) volume (mL) and spinal cross-sectional area (CSA, mm2) were obtained after segmentation of brain T1w and the spinal cord T2s contrasts, respectively. Linear regressions between CSA and brain GM volume are shown for the subcortical area, cerebellum, and somatomotor cortex. Reported p-values correspond to the main effect of brain area and area x age interactions. Solid line represents the linear regression fit, the shaded gray area indicated the 95% confidence interval, and each dot represents an individual participant (*n* = 67), color-coded by age from green (younger) to pink (older). **B** Spinal cord GM CSA and GM volume for each brain network were entered into regression models, including age and sex as fixed effects. Bar plots represent age-related effects (*t* values) for each network (spinal cord network highlighted in gray). **C** Brain or spinal cord time-series were extracted and correlated between regions of interest to measure functional connectivity (FC). Boxplots show intra- (left) and inter-networks (right) FC across ten networks, with the spinal cord network highlighted in gray. Each box represents the distribution (i.e., from the 25th to the 75th percentile), whereas the medians are represented by the horizontal white line inside the box. Vertical extending lines denote the extreme values within a 1.5 interquartile range. Each dot is an individual participant (*n* = 67), color-coded by age from green (younger) to pink (older). **D** FC values were entered in linear mixed models (LMMs) to assess age-related changes. The significance of each fixed effect was assessed using a two-sided *t* test. Bar plots display age-related effects (*t* values) across networks, with yellow representing inter- and pale-yellow intra-network FC. Circled asterisks indicate significant *t* values after FDR correction (*P* < 0.05). **E** Parcel-specific intrinsic dynamic features were derived from timeseries across both brain and spinal cord structures. Principal component analysis (PCA) was applied to the parcels x timeseries features matrix to identify dominant patterns of time-series dynamic variation. To access how strongly each network expresses the first (top) or second (bottom) principal component, feature matrices were projected onto PCA-derived eigenvectors, yielding parcel-wise scores. Each dot represents the centroid score of parcels within a network, positioned along the respective gradient. **F** Age effects on timeseries dynamic features were estimated using LMM. The heatmap represents *t* values for age effects across dynamic features across networks (green: decrease, pink: increase with age). Significant effects before and after FDR correction are represented by asterisks or circled asterisks, respectively.

To investigate the structural and functional hierarchical organization from the cortex down to the spinal cord and its age-related changes across CNS levels, we conducted structural and functional MRI scans, the latter being acquired simultaneously for the brain and spinal cord. First, the relationship between spinal cord and brain structural integrity was assessed to determine whether age-related changes occur in parallel across regions. We performed brain-level analyses across 200 brain cortical regions, 24 cerebellar regions, and six subcortical structures (combining Schaefer and Cobra atlases)^44,45^. This parcellation enabled the investigation of nine brain networks, along with the whole cervical spinal cord network. To simplify the description of the results, in the main text, we focused on the networks related to somatomotor processing (i.e., the somatomotor, spinal cord, cerebellar, and subcortical networks) in Fig. 4A, B, while the results from the other networks are provided in the Supplementary Material. Across these three brain networks, we did not find any significant relationship between the spinal cord GM cross-sectional area (CSA) and the brain GM volume (Fig. 4A, Subcortical: *t*(66)= −0.64, *P* = 0.52; Cerebellum: *t* = −1.33, *P* = 0.19; Somatomotor: *t*(66)= −1.73, *P* = 0.088). Furthermore, there were no significant differences in the age effect between the spinal cord CSA and the subcortical or somatomotor region volume. However, the spinal cord appeared to be more strongly affected by age compared to the cerebellum, as indicated by a significant age-by-region interaction (*t* = 2.20, *P* = 0.031). Importantly, all ten networks revealed strong volumes or CSA decreasing with aging (Fig. 4B, Supplementary Fig. S8, and Table S7).

Next, we aimed to deepen our understanding of the functional organization spanning the brain and the spinal cord by computing the average intra-network FC (i.e., FC between parcels within the same network) and inter-network FC (i.e., FC between parcels belonging to distinct networks) for each of the ten networks. Across all networks, intra-network FC was consistently higher than inter-network FC (Fig. 4C and Supplementary Table S8; *t*(66) = 40, *P* < 0.001), while a weaker but positive FC was observed between networks at each CNS level (*t*(66) = 14.8, *P* < 0.001; Supplementary Table S9). This pattern held for both brain and spinal cord networks, suggesting a shared organizational principle of network segregation at rest, characterized by strong, coherent FC within the same network and relatively weak but consistent FC across them. Next, we examine age-related functional connectivity changes in both brain and spinal cord networks to determine whether functional reorganization with aging follows a system-wide pattern or emerges differentially across specific networks. The aging effect was assessed using a linear mixed model for both intra- and inter-network FC, including age and sex as fixed effects and participants as a random effect. We found a significant increase in the spinal cord and brain inter-network FC with aging, suggesting a shift toward less specific FC patterns, reflecting less segregated networks^46^ at each CNS level (Fig. 4D, F and Supplementary Table S10). In contrast, spinal cord intra-network FC did not exhibit significant age-related alterations, consistent with most brain networks, except for the posterior networks (visual, dorsal attentional, and default mode) and the cerebellum, which showed an increase in intra-network FC with aging (Fig. 4D and Supplementary Table S11).

To situate spinal intrinsic dynamics within a broader CNS organizational framework, we investigated the topographical organization of time-series dynamics across each level of the CNS. Principal component analysis (PCA) applied to the parcels-by-features matrix extracted independent patterns of intrinsic dynamics, with the first two components collectively accounting for 62% of the variance. The first principal component (PC1) captured a cortical–subcortical-spinal cord gradient of intrinsic dynamics (Fig. 4E, top). Within the cortex, this gradient followed the well-established dorsolateral-ventromedial axis of functional organization^47,48^. The second component (PC2) revealed a unimodal-transmodal gradient of intrinsic dynamics^1,5,47,48^ Fig. 4E, bottom). The unimodal end of this gradient comprised the visual cortex, followed by somatomotor regions, including the somatomotor cortex, spinal cord, and subcortical structures (cerebellum, basal ganglia). Progressing along the gradient, it encompassed heteromodal associative areas capable of integrating information across modalities and finished in default mode regions, which are widely recognized as transmodal.

To assess age-related changes in dynamic functional organization across the CNS, we applied LMMs to each time-series feature, including age and sex as fixed effects and participants as random effects. Notably, ALFF and standard deviation (SD), key metrics of signal amplitude fluctuation, together with entropy pairs, a measure of temporal complexity, significantly increased with age across several networks (Supplementary Table S12). These widespread changes suggest that aging is associated with a general increase in spontaneous neural fluctuation throughout the CNS.

## Discussion

Despite the spinal cord’s key role in somatomotor function and its clinical relevance in conditions such as spinal cord injury, multiple sclerosis, and age-related pathologies like Parkinson’s disease, in vivo neuroimaging of the human spinal cord has historically lagged that of the brain. This disparity stems from significant technical challenges in imaging a deep, narrow structure surrounded by different tissues, cerebrospinal fluid, and moving organs. Over the past decade, however, substantial progress in both acquisition and analysis techniques has enabled more precise microstructural imaging and the extraction of robust functional signals from the spinal cord^9–11^. Leveraging these advances, we built a unique multimodal dataset spanning the entire cervical spinal cord and brain in a cross-sectional cohort of 70 healthy young and older adults. This integrative approach allows us to reveal that spinal somatosensory regions (dorsal) are predominantly affected by aging. Altogether, we identified region-specific aging effects, quantified structure-function coupling in the spinal cord, and revealed shared organizational principles across the CNS.

Using distinct microstructural and functional analyses, we observed pronounced age-related changes in the dorsal spinal cord, which is primarily responsible for somatosensory processing. Older adults exhibited reductions in white matter, gray matter volume, magnetization transfer ratio (MTR), and diffusion metrics such as fractional anisotropy (FA) and axial diffusivity (AD), alongside increased radial diffusivity (RD), consistent with declines in tissue integrity, potentially reflecting neural shrinkage, impaired axonal organization, or dys/demyelination. These results align with previous research showing that aging leads to a preferential loss of large myelinated sensory fibers, reduced conduction velocity, and impaired somatosensory acuity^49^. Electrophysiological assessments of proprioceptive-motor loops (H-reflex) in humans have similarly demonstrated greater deterioration in the sensory component compared to the motor component with aging^50–53^. Extending these findings, our complementary tract-specific microstructural analyses revealed significant demyelination in dorsal WM columns, associated with tactile and proprioceptive afferents, whereas no such changes were observed in WM ventral columns, which mainly contain nociceptive and thermoceptive fibers^54^. This pattern mirrors a prior ex vivo study showing that the dorsal column is particularly susceptible to age-related degeneration^55^, supporting the hypothesis that aging may differentially affect the somatosensory system, with proprioceptive afferents being particularly vulnerable^23,56–58^. Our functional analyses further mirrored these changes: the dorsal cord exhibited increased local spontaneous activity (higher SD and ALFF), reduced signal richness (decreased low-frequency power), and enhanced predictability (increased autocorrelation). In addition, increased functional connectivity was found between the dorsal and ventral horns that primarily involves the left dorsal quadrant (i.e., left dorso-ventral and left dorsal-right ventral FC). Ipsilateral dorso-ventral connectivity, reflecting the relay of somatosensory signals to motor circuits (sensorimotor integration), may be sensitive to age-related alterations in spinal reflex loops^59^. Changes in connectivity between the left dorsal horn and the contralateral ventral horn may, in turn, reflect modifications in cross-communication via commissural interneurons involved in cross-limb coordination^60^. These connectivity and spontaneous activity findings align with rodent studies showing age-related dorsal hyperexcitability and shifts in excitation-inhibition balance^32,33,35,61^. Interestingly, in our study, the left dorsal quadrant, which receives somatosensory input from the participants’ non-dominant upper limb, was most affected. While this study did not allow us to determine the reason for this lateralization, it may reflect differences in limb use, horn specialization, or other factors, highlighting a potential target for future studies and somatosensory rehabilitation.

Across both microstructural and functional metrics, the ventral horn showed less pronounced age-related in the ventral horn. The literature on age-related changes in the ventral horn is inconsistent regarding whether motor neurons are lost or undergo atrophy with aging^62–65^. Nonetheless, there is some evidence of age-related alterations, including a reduction in the excitatory-inhibitory synaptic ratio and an increase in senescent motor neurons^62,66^. Although the functional effects in the ventral quadrants were less pronounced in our analyses, these previous findings align with the microstructural changes that we also observed in the ventral horns (i.e., reduction in spinal cord gray and white matter as well as microstructural integrity with aging). To better understand these region-specific changes with aging along the sensory-motor axis, future studies should investigate spinal cord function beyond resting-state assessments by incorporating motor tasks, somatosensory-stimuli paradigms or sensorimotor decline assessment, allowing a more comprehensive evaluation of somatosensory and motor alterations associated with aging.

Beyond separate spinal cord structural and functional analyses, we examined their interplay and how this coupling evolves with aging. We uncovered a close structure-function correspondence along the cervical spinal cord, consistent across measures of functional organization, whether assessed through inter-regional connectivity or local spontaneous activity. This tight coupling mirrors the structural-functional coupling observed in the literature for the primary somatomotor cortex, where highly specialized afference and efference support greater correspondence between structure and function compared to other brain regions^2,47^. Like the somatomotor cortex, the spinal cord exhibited a highly specialized functional architecture, exemplified by reflex loops, the most stereotyped stimulus-response correspondence, which can involve as few as a single synapse between somatosensory afference and motor efference, directly linking sensation and action^67^. Importantly, the spinal structural-functional coupling is not static but changes with aging, with structural decline associated with altered functional connectivity and local dynamics. These findings highlight the need for multidimensional functional metrics to fully capture its reorganization and develop sensitive biomarkers of spinal cord aging or dysfunction. We speculate that the structural-functional coupling changes with aging may reflect neural plasticity, such as the emergence of compensatory mechanisms, but could also result from structural alteration, such as demyelination, that slows and reduces the fidelity of the signal transmission, ultimately leading to increased inter-segmental connectivity or greater signal variability (i.e., increase ALFF, standard deviation). Combining behavioral assessments with multimodal imaging will be critical to distinguish adaptive and maladaptive changes and to determine how this structural-functional correspondence evolves in other contexts, such as motor learning, injury recovery, or neurodegenerative diseases.

We next asked whether shared structural and functional organizational principles span the architecture of the CNS across its different levels. Mapping the connectome beyond the cortex, particularly in structures like the spinal cord, is essential to understand how the CNS interfaces with the body and the environment, as well as how this relationship evolves across the lifespan. Recent studies have begun to integrate multimodal imaging of both cortical and extracortical structures, including the brainstem or the spinal cord, as well as peripheral organ systems, to provide a more comprehensive view of CNS-body communications^20,68,69^. Building on this emerging framework, we leveraged neuroimaging data from simultaneous imaging of the spinal cord and brain within the same individuals, uniquely positioning us to examine CNS-wide patterns of organization and their age-related changes. This approach not only deepened our understanding of brain organization but also enabled us to validate and contextualize our spinal cord findings, revealing common principles of neural architecture and shared age-related changes across the neuroaxis. Notably, shared organizational principles were more apparent in functional than in structural analyses. While functional metrics revealed consistent patterns across spinal cord and brain regions involved in sensorimotor processing, structural volumetric analyses did not show a significant correlation between the spinal cord and brain regions, consistent with previous reports that brain GM volume does not predict spinal cord GM CSA^30,70^. Examining functional connectivity organization revealed similar patterns of connectivity in both structures, characterized by stronger connectivity within networks and weaker connectivity between distinct networks. Analogous to the brain, inter-network FC in the spinal cord increased with aging. This phenomenon, well-documented in brain regions such as the primary somatomotor cortex^71–73^, is associated with dedifferentiation (i.e., a decrease in neural specialization), which manifests as more widespread connectivity across networks or a recruitment of additional brain areas during motor tasks and somatosensory stimulation^23–25^. Our results suggest that a similar reduction in network segregation extends across the CNS. Future studies could explore whether these spinal connectivity changes are linked to cortical networks and vice versa by examining cortico-spinal resting-state connectivity patterns^20,22^.

We further examine local time-series dynamics across brain and spinal cord regions, using a principal functional gradient approach, to characterize the intrinsic macroscale organization of CNS activity^1^, independently of aging effects. This analysis revealed two continuous patterns of intrinsic organization. One pattern captured a cortical–subcortical–spinal cord axis, spanning the occipital cortex to the spinal cord. This gradient extended the well-established anteroposterior cortical gradient^47,48^ by integrating subcortical regions and the spinal cordand thereby capturing the neuroaxis of the human CNS along a rostro-caudal coordinate system. The second pattern followed a unimodal-transmodal gradient, from the visual network to the default mode network^1,5,47,48^. Remarkably, the cerebellum, subcortical nuclei, and the spinal cord were positioned close to the somatomotor network along this gradient, likely reflecting their shared functional involvement in somatomotor processing. Together, these gradients provide compelling evidence for a hierarchical and continuous functional organization across the CNS, bridging cortical, subcortical, and spinal levels. We further examined age-related changes in local spontaneous activity throughout the CNS and found that increased signal variation (higher ALFF and standard deviations) was a consistent feature. This pattern suggests that aging is associated with a generalized shift toward less stable neural activity, potentially driven by reduced neural specialization, compensatory recruitment of additional neural resources, or greater physiological noise. These findings reinforce the notion that aging induces widespread functional reorganization, not only in the brain but across the entire CNS.

In the present study, we extended the concept of the in vivo cortical connectome to the spinal cord and characterized its age-related changes using multimodal imaging and multidimensional analyses. This opens avenues for understanding the trajectory of the somatomotor system across the lifespan and holds promise for clinical application. For example, both the brain and spinal cord are affected in Parkinson’s disease^18,74–76^, yet they are not studied together. Applying integrated structural and functional analyses across these structures could yield deeper insights into disease mechanisms and help identify early biomarkers of somatomotor dysfunctions. Moreover, our observation of age-related region-specific alterations in the spinal somatosensory pathways raises important questions about the underlying peripheral *versus* central mechanisms driving these changes. Future longitudinal studies, combining neuroimaging, electrophysiology, and behavioral measurement, will be essential to track the temporal evolution of the CNS-wide reorganization with aging. Ultimately, this work lays the foundation for a more comprehensive understanding of the neural basis of sensorimotor aging and its disruption in neurological conditions.

Despite the strengths of our multimodal and multilevel approach, several limitations warrant consideration. First, although we observed consistent patterns of age-related change, our cross-sectional design limits causal inference regarding the temporal dynamics of CNS aging. Longitudinal studies will be essential to disentangle progressive decline from compensatory reorganization and to explore potential nonlinear trajectories of aging. Second, our study focused on the resting state to characterize functional organization and its age-related changes. It is important to recognize the complementary insights offered by task-based paradigms, and sensorimotor decline assessment can more directly probe functional specialization and recruitment patterns during sensorimotor engagement. Finally, a further limitation of our study is the lack of microstructural imaging data at the brain level, which prevented direct comparisons between spinal and cerebral fine-scale tissue integrity. Future studies incorporating advanced brain microstructural imaging techniques will thus be essential to fully characterize the interplay between structure and function across the entire CNS.

In summary, we leveraged recent advances in neuroimaging to extend the scope of aging research beyond the brain, encompassing the spinal cord. Our findings highlight the continuity and specialization of sensorimotor systems across the neuroaxis and reveal shared patterns of age-related reorganization. This work opens avenues for identifying CNS-wide biomarkers and therapeutic targets for age-related neurodegenerative disorders.

## Methods

### Participants and MRI acquisitions

Seventy right-handed healthy participants (36 females; age 48.82 ± 16.94 years old [20–80], years) were included in this study. The experiment was approved by the local ethics committee (McGill University Health Centre REB 2019-4626), all participants gave their written consent in accordance with the Helsinki Declaration and received a compensation of 50 CAD for their participation. The MRI data were acquired at the Neuro (Montreal Neurological Institute, Canada) using a 3-Tesla MRI Scanner (Magnetom-Prisma^fit^, Siemens, Erlangen, Germany) equipped with a 64-channel head and neck coil. Participants were positioned head-first supine and were instructed to relax, minimize motion, and swallow. Throughout the scanning session, the participants wore Neck and Brachial Plexus SatPads around their neck and on their chest, respectively, as well as physiological sensors that included a pulse sensor on their index finger and a respiration belt (Siemens Physiology Monitoring Unit).

A high-resolution T1w anatomical image covering the whole brain and the cervical spinal cord up to the first thoracic vertebrae was acquired to facilitate tissue segmentation, as well as vertebral labeling for normalization to the PAM50 template^77^ for spinal functional and microstructural. This acquisition was followed by simultaneous brain/spinal cord echo planar imaging (EPI) (same field of view as the T1w image) to investigate brain and spinal cord activity during rest (i.e., no explicit task), while participants were instructed to refrain from specific thoughts and passively view the *Inscapes* video^78^. The microstructural images were acquired at the cervical spinal level (C1 to C7 vertebrae), including T2*w, magnetization transfer (MT) and multi-shell diffusion (DWI) MRI sequences. The detailed acquisition parameters for each image are summarized in Table 1, and data from two representative participants are represented in Supplementary Fig. S1. Thus, microstructural images were specific to the spinal cord, while functional and T1w images were simultaneously acquired for the brain and the spinal cord. Further analyses of structural integrity will be consequently based on T2*w, DWI, and MT images for the spinal cord, while the brain analyses will be done based on the T1w image.

**Table 1:** Acquisition parameters for each contrast image

	T2*w	Magnetization transfert (GRE-MT1/GRE MT0)	DWI	T1w (MPRAGE)	EPI
Structure coverage (top/bottom)	Brainstem/ vertebra C7	vertebrae C1–C7	vertebrae C1–C7	Top of the brain/ vertebra T1	Top of the brain/ vertebra T1
Coil elements	HC: 1–7; NC: 1–2	HC: 5–7; NC: 1–2	HC: 7; NC: 1–2	HC: 1–7; NC: 1–2	HC: 1–7; NC: 1–2
Field of view	180 × 190 × 162 mm^3^	230 × 240 × 166 mm^3^	86 × 41 × 122 mm^3^	229 × 365 × 375 mm^3^	192 × 213 × 316 mm^3^
Orientation	Transversal	Transversal	Transversal	Transversal	Transversal
Slices (*n*)	30	30	24	288	69
Volumes (*n*)	–	–	–	–	230
Voxel size	0.4 × 0.4 × 5 mm^3^	0.9 × 0.9 ×5 mm^3^	0.9 × 0.9 × 5 mm^3^	1.3 × 1.3 × 1.3 mm^3^	1.6 × 1.6 × 4 mm^3^
iPAT mode	Grappa	Grappa	–	Grappa	Grappa
TR	34 ms	35 ms	780 ms	2300 ms	1550 ms
TE	14 ms	2.62 ms	64 ms	3.3 ms	23 ms
Flip angle	5	9	90	9	70
Acceleration factor (phase encoding)	2	2	–	2	2
Multiband (slice encoding)	–	–	–	–	3

T2*w T2-star weighted, *GRE-MT1/GRE-MT0* gradient echo with magnetization transfer (on/off), *DWI* diffusion-weighted imaging, *T1w* T1-weighted, *MPRAGE* magnetization prepared rapid gradient echo, *EPI* echo planar imaging, *HC* head coil, *NC* neck coil, *TR* repetition time, *TE* echo time, *iPAT* integrated parallel acquisition technique, *GRAPPA* generalized autocalibrating partially parallel acquisition; *FOV* field of view.

### Data curation

Data were sorted, transformed into NIFTI, and organized using the Brain Imaging Data Structure (BIDS) standard (with dcm2bids v2.1.4). We used an in-house python pipeline^79^ (publicly available) based on the Spinal Cord Toolbox (SCT, version 5.6.0)^80^, the Oxford Center for fMRI of the Software Library (FSL, version 5.0), the Statistical Parametric Mapping (SPM12, running on Matlab 2021b), the Tapas PhysiO toolbox (release 2022a, V8.1.0)^81^ and the Nilearn toolbox (version 0.9.1).

### Structural data processing

After visual inspection of the structural images, three participants were excluded due to motion artifacts on T1w and T2*w images; five from the MTR and 12 from DWI due to data quality issues. For analyses combining multiple contrasts, only the remaining common 54 participants were included (Supplementary Table S1).

#### Brain and spinal cord preprocessing

First, the T1w image was preprocessed, starting with the separation of brain and spinal cord slices to perform tailored preprocessing on each structure.

Brain tissue segmentation was performed automatically using the CAT12 toolbox (an SMP12 extension), which employs the DARTEL (Diffeomorphic Anatomical Registration using Exponentiated Lie Algebra) algorithm to categorize brain voxels into gray matter (GW), white matter (WM), and cerebrospinal fluid (CSF)^82^. A study-specific brain template in MNI space was generated from all participants’ brain T1w images using DARTEL. This study-specific template enables better inter-participant alignment by iteratively optimizing deformation fields to minimize anatomical variability, which is particularly beneficial when analyzing participants across a wide age range.

Spinal cord segmentation included the GM and WM; it was performed automatically (with SCT, *sct_propseg*), and manual adjustments were applied when necessary. First, the T1w image was warped into the PAM50 space (0.5 × 0.5 × 0.5 mm^3^) using cord segmentation and disk labeling (with SCT, *sct_register_to_template*). Next, spinal cord segmentation was computed on T2*w, MT0, MT1 *(i.e.,* with and without an MT pulse), and the mean DWI images by automatic methods (with SCT, sct_propseg) with manual corrections when necessary. Specific preprocessing was also applied to each contrast image. The GM of the T2*w images was automatically segmented (with SCT, sct_deepseg_gm), the MT0 image was registered to the MT1 image, and DWI images were corrected for motion correction (with SCT, sct_dwi_moco). Finally, the three images were normalized into the PAM50 template using their segmentation as well as the T1w to PAM50 warping field to initialize the registration (with SCT, *sct_register_multimodal*).

#### Spinal cord microstructural metric extraction

Microstructural analyses were conducted in the participant’s native space. We used the previously generated warping field to transform the PAM50 template to the individual space to extract metrics within the spinal levels atlas (SCT v6.1)^37^. Because of the limited reliability of automatic tissue segmentation at the C1 level, attributable to poor anatomical contrast, microstructural analyses were confined to segments C2 to C7 where tissue boundaries could be delineated more robustly. First, we calculated the number of voxels within the GM or the WM in the T2*w image (voxel counts) from the automatic segmentation of the image in individual native space. The magnetization transfer ratio (MTR) was computed for each voxel using the coregistered MT0 and MT1 images (*sct_compute_mtr)*. Finally, we computed fractional anisotropy (FA), mean diffusivity (MD), radial diffusivity (RD), and axial diffusivity (AD) from the DWI images using DIPY library via the SCT (*sct_dmri_compute_dti*). The average values of these microstructural metrics were extracted within the C2 to C7 spinal levels. Each spinal level was divided into four quadrants (ventral-left, ventral-right, dorsal-left, dorsal-right), resulting in a total of 24 spinal cord parcels (Fig. 1A). Each quadrant includes both gray and white matter, as some quantitative MRI metrics are sensitive to microstructural properties of both tissue types. Complementary tract-specific analyses (dorsal columns, ventral funiculi, and lateral funiculi) were also conducted for MT and DWI metrics. We choose to define levels based on spinal segments rather than vertebral levels to more accurately reflect the functional organization, which aligns with the rootlet insertion^17^, thereby enabling a more robust comparison with our functional results.

#### Spinal cord morphometric similarity matrix

We derived the spinal cord morphometric similarity matrix using the same procedure as that employed for the brain^83^. Specifically, each of the 24 spinal regions was represented as a vector of multiple structural features (GM voxel counts, WM voxel counts, MTR, FA, MD, AD, RD) (Fig. 1B). Each of the feature vectors for each individual was normalized (z-scored). Pairwise Pearson correlations were computed as a measure of similarity between feature vectors of all parcel pairs to generate individual-level morphometric matrices (24 × 24). To estimate the within and between segmental networks similarity, the mean similarity was calculated for intra-segmental (i.e., the four parcels from the same spinal level) and inter-segmental (i.e., parcels from distinct spinal levels) parcel pairs. Finally, we average the matrices across individuals to obtain a group-level morphometric matrix.

#### Age effects and prediction analyses

Age effects were estimated for each microstructural metric within each quadrant (ventral-left, ventral-right, dorsal-left, dorsal-right) using linear mixed models. Each model included all spinal levels within the quadrant as repeated measurements per participant, with participant as a random effect (see “Statistical analyses” for details). Thus, reported results summarize effects at the quadrant level. In addition, Microstructural features were used to predict participants’ age. Given the strong structural, asymmetric, and distinct functional role between the ventral and dorsal divisions of the spinal cord^11^, age prediction analyses were conducted separately for these two divisions. Individual age values were predicted from all microstructural features extracted from the ventral or dorsal parcels using an elastic net regression model (alpha = 1.2 and lambda = 1). To validate the model, we performed a fivefold cross-validation. In each iteration, the dataset was randomly split into fivefolds, with approximately 10–11 participants (20%) allocated for testing, while the remaining participants (80%) were used for training. This process was repeated 10 times, resulting in a total of 50 train-test evaluations. The mean absolute error (MAE) and the Pearson’s correlation coefficient (*r*) between predicted brain age and chronological age were averaged across the repetition to increase the robustness of the results. In addition, we report *R*², calculated as the squared Pearson correlation coefficient (*r*²), representing the proportion of variance in chronological age explained by the model predictions. For each iteration, the model produces regression weights for each predictor, i.e., each feature, corresponding to the importance of each feature in predicting age. To ensure that our age prediction models were robust and not driven by spurious correlations, we performed permutation testing (1000 permutations) by randomly shuffling age labels across participants. ElasticNet models were re-fitted for each permutation, generating null distributions of *R*² and MAE values.

#### Brain and spinal cord structural metric extraction

Structural metrics were extracted in the participant’s native space. To investigate the relationship between the structural integrity of the spinal cord and that of the brain for each individual, we computed spinal cord GM cross-sectional area (CSA) and brain GM volume. The motivation for using these two metrics is that they are the most widely used structural biomarkers for assessing spinal cord or brain atrophy, as they can be derived from various structural contrasts (T1w, T2w, MT). In addition, they were selected as the most comparable metrics between the brain and spinal cord. Here, we used the GM segmentation derived from T2*w images for the spinal cord to compute GM CSA and T1w images to calculate brain GM volume. Specifically, spinal cord structural integrity was assessed using GM cross-sectional area (CSA) measured for each of the six segmental levels from C2 to C7 (via SCT, *sct_process_segmentation*). By contrast, brain structural integrity was computed using total GM volume (in mL) across 200 GM cortical (Schaefer atlas 7Networks)^44^, 24 GM cerebellar and six subcortical structures (from the Cobra atlas)^45^, extracted via the CAT12 toolbox. Finally, for each of the ten networks (seven Schaefer’s cortical, spinal cord, cerebellar, and subcortical networks), we calculated the total structural integrity by summing brain GM volumes or by computing the mean GM-CSA across all corresponding parcels.

First, we estimated the relationship between spinal cord CSA and each brain network structure using an OLS model, with age and sex included as fixed effects. Then, we tested the interaction between brain network GM volume and age to determine whether age modulates the spinal cord-brain structure relationship (see Supplementary Table S2 and “Statistical analyses” for details).

### Functional data processing

The same 67 participants included for T1w and T2s analyses were also included for functional analyses (Supplementary Table S1). Functional processes are similar to those developed in our previous studies^17,18,20^.

#### Preprocessing

Slice-timing correction was applied to the functional images, followed by the separation of brain and spinal cord slices to enable structure-specific preprocessing, which included the following steps:

i) *Motion correction*. Spinal cord motion correction used slice-wise realignment and spline interpolation (SCT, *sct_fmri_moco*) and was performed inside a 30 mm cylindrical mask centered on the spinal cord centerline, including the spinal canal. At the brain level, we removed the non-brain tissue FSL, BET) before performing motion correction with rigid-body realignment (FSL, MCFLIRT). Using the realignment motion parameters, we calculated the framewise displacement (i.e., *FD*, motion between two consecutive volumes) at both spinal cord and brain levels. Three participants with FD > 0.3 mm either at the brain or spinal cord levels were excluded. For the remaining 67 participants, we test age-effect on FD by applying OLS models on the brain and spinal cord FD (Supplementary Fig. S2B). Then, we computed the temporal signal-to-noise ratio (tSNR) to evaluate the quality of the functional data over time. For each voxel, tSNR was calculated by dividing the mean signal intensity across time by its standard deviation. tSNR was not used as a criterion for excluding participants; it was reported for descriptive quality control and to compare data quality across participants of different ages.
ii) *Image segmentation*. The resulting mean motion corrected image was segmented in two steps for the spinal cord: the spinal cord centerline was extracted manually, and the cord (GM + WM) and cerebrospinal fluid (CSF) were segmented (SCT, *sct_propseg*). Tissue segmentations were manually corrected when necessary.
iii) *Normalization into the MNI or PAM50 template*. Similar to the microstructural images, the spinal cord mean motion corrected functional image was normalized into the PAM50 template using its cord segmentation as well as the T1w to PAM50 warping field to initialize the registration (with SCT, *sct_register_multimodal*). At the brain level, normalization was done in three steps using SPM12. First, we coregistered the mean functional image to the T1w space. Next, we warped the T1w image into the MNI template (2 × 2 × 2 mm^3^). Finally, the resulting deformation field was applied to move the functional images into MNI space. The functional image to template space warping fields were applied to the spinal cord and brain 4D functional images after the denoising steps.

#### Time series denoising

We modeled slice-wise nuisance regressors to account for physiological noise for each participant. We used the same denoising approach for both brain and spinal cord data, with the only difference being the number of CompCor components applied at each level. First, we used the RETROspective Image CORrection (RETROICOR) procedure^84^ to compute noise regressors from peripheral physiological recordings (heart rate and respiration, Tapas PhysiO toolbox, an SPM extension)^81^. Specifically, we modeled four respiratory harmonics, three cardiac, and one multiplicative term for the interactions between respiratory and cardiac noise (18 regressors in total, similar to^20,85^ and adjusted the regressors timing to each slice. Second, we used the CompCor approach^86^ to identify non-neural fluctuations by extracting the first principal components of the unsmoothed brain or spinal cord cerebrospinal fluid (CSF) signal in the participant’s native space (12 components for the brain slices and 5 components for the spinal cord slices). The first five slice-wise discrete cosine transform (DCT) basis functions were added for detrending. These nuisance regressors were finally combined with the six brain and two spinal cord slice-wise motion parameters. The removal of the noise confounds was applied slice by slice and based on a projection on the orthogonal of the fMRI time-series space and was applied orthogonally to a band-pass temporal filter (0.01–0.17 Hz, with Nilearn, *img.clean_img*). No smoothing, nor any signal standardization, was applied to the data at this step of the processing.

#### Functional connectivity analyses

Functional connectivity was performed on the denoised time-series, normalized in template space, and z-scored.

#### ICAPs framework

We employed the iCAPs framework to identify seven spinal cord segments in a data-driven manner^17,87^. This approach extracts transient activity using total activation^88^ and the temporal clustering of these signals. Specifically, a regularized hemodynamic deconvolution was used to extract activity-inducing signals from the smoothed, denoised, normalized, z-scored, and unfiltered time series. Innovation signals (i.e., temporal derivative of the activity-inducing time series) were then clustered using K-means to generate group-level innovation-driven coactivation patterns (iCAPs). To assess the spatial similarity with Frostell’s atlas^37^, we computed the Dice coefficient between the resulting components and Frostell’s segmental levels.

#### Spinal cord functional connectivity analysis

The spinal cord was divided into four quadrants along the dorsal–ventral and left-right axes, across the seven spinal cord levels (i.e., 28 parcels). Each quadrant included both gray and white matter, ensuring comparability with structural analyses while minimizing the partial volume effects between tissue types. For each individual, the unsmoothed and band-pass filtered time series within each parcel were extracted and averaged. The level of functional connectivity (FC) was then computed using Pearson’s correlation coefficient between each pair of parcel time-series, resulting in a 28 × 28 individual-level spinal functional connectivity (SpiFC) matrix. Individual matrices were then converted using Fisher’s r-to-z transformation. The population-level SpiFC was constructed as the mean SpiFC across all individuals. The mean FC was also calculated for intra- and inter-segmental level parcel pairs to assess intra- and inter-network FC. The age effect was assessed using a linear mixed model (see “Statistical analyses” for details and Supplementary S2) for intra- or inter-segmental levels of the six parcel pairs (ventro-ventral, dorso-dorsal, right dorso-ventral, left dorso-ventral, right dorso- left ventral, and left dorso-right ventral).

#### Analysis of intra- *versus* inter FC and age effects across the brain and spinal cord

To assess whether the spinal cord FC organization is similar to that of the brain, we conducted parallel analyses at the brain level using data from the same individuals. The brain was parceled into 200 cortical areas using the seven-network Schaefer atlas^44^, along with 24 cerebellar and six subcortical regions using the Cobra atlas^45^, yielding a 230 × 230 individual-level brain functional connectivity (BrainFC) matrix. The brain parcels were grouped into cerebellar, subcortical, and seven cortical networks. To evaluate the FC organization across the CNS, we computed intra- and inter-network FC separately within the spinal cord (averaged across the seven spinal segments, each treated as a distinct network) and the brain (one subcortical, one cerebellar, and seven cortical networks). This parallel approach allowed us to investigate systematically inter- *vesus*. intra-network FC across CNS regions and examine whether the spinal cord follows similar network organization principles as the brain does. Finally, we investigated the age-related changes in this FC organization. Specifically, using LMMs, we tested the age-effect for each intra- and inter-network FC of the brain or spinal cord (see “Statistical analyses” and Supplementary S2 for details).

#### Time-series dynamic properties extraction

Time-series dynamic properties extraction was performed on time-series denoised, unsmoothed, and normalized in template space.

#### CAnonical Time-series Characteristics (catch-22) extraction

For a given spinal cord parcel (28 parcels, from C1 to C7 segmental levels, divided into four quadrants), the dynamic properties of the z-scored BOLD time-series were summarized using a set of features extracted with the Canonical Time-series Characteristics python toolbox, catch22^43^. The catch-22 features were based on the HCTSA toolbox^89^ and designed to be a highly explanatory subset of features typically encountered in time-series (while not specifically defined for BOLD signal). We excluded five features that exhibited a dominant single value in more than 25% of the observations (i.e., across spinal parcels and participants), as such features inherently lack variability and discriminative ability, violating the assumptions that features capture meaningful variability (Supplementary Fig. S9). We also add to these features the calculation of the standard deviation and the mean on non-z-scored time series. To compare our data with a standard biomarker commonly used at the spinal cord level^40–42^ to assess local resting-state activity, we calculated the amplitude of low-frequency fluctuations (ALFF). ALFF was computed by first deriving the power spectrum of the detrended non-z-scored BOLD time series at each voxel, then taking the square root of the mean power within the low-frequency range (0.01–0.027 Hz). The average ALFF value was then calculated within each of the 28 spinal cord parcels. In total, we obtained 20 time-series dynamic features (17 from catch-22, mean, standard deviation, ALFF) that include, but are not limited to, distributional properties, autocorrelations, and variability of a given time-series.

#### Spinal cord dynamic profile similarity matrix (SpiDyn)

To compute the dynamic profile similarity matrix across the 28 spinal parcels, each feature was normalized across spinal cord parcels to the unit interval using a scaled robust sigmoid function, ensuring comparability across the 20 features with different value ranges^90^. Similar to the approach used for computing the morphometric matrices, the spinal cord dynamic profile similarity matrices (SpiDyn matrices) were generated by calculating pairwise Pearson correlations between the 20-dimensional feature vectors across all parcel pairs, resulting in individual-level 28 × 28 similarity matrices (see details in “Spinal cord morphometric similarity matrix“).

#### Modeling age change in time-series dynamic features

When analyzing each time-series dynamic feature to test the age effect, normalization or rescaling was not applied to preserve interpretability in the original feature scale and avoid introducing distortions. We estimated linear age-related changes using LMMs. Each model included spinal levels within the parcels (i.e., right-ventral, left-ventral, right-dorsal, and left-dorsal) as repeated measurements per participant, with participant as a random effect (see “Statistical analyses” and Supplementary Table S2 for details).

#### Analysis of local time-series dynamics and age effects across the brain and spinal cord

To assess the topographical organization of time-series dynamics across the CNS, we applied principal component analysis (PCA; *n* = 10 components) to the parcels-by-features matrix (254 parcels × 20 features) to capture independent patterns of time-series dynamics. The resulting component loadings were summarized by computing the centroid for each of the predefined networks (nine brain and two spinal cord networks). For this analysis, the spinal cord was subdivided into ventral and dorsal networks (encompassing, respectively, left and right ventral or dorsal parcels) to test whether an intrinsic functional dissociation or association exists between these subdivisions, analogous to the antero/posterior or unimodal/transmodal gradients observed at the cortical level^5,48^. Through this analysis, we aimed to uncover the gradient of functional dynamics spanning the whole CNS (cortical, subcortical, and spinal cord), thereby revealing the large-scale organization principle.

To assess age-related changes in local dynamics, we used LMMs for each network (nine brain and two spinal cord networks) and feature, using age and sex as fixed effects, as well as participant as a random effect (see “Statistical analyses” for details).

#### Structural and functional coupling

Structural and functional coupling was estimated across the spinal cord using participant-averaged matrices. For each matrix (microstructural, SpiFC, SpiDyn), individual matrices were first stacked and averaged across participants to produce group-level matrices. From these matrices, only the upper-triangle edges were extracted (n_nodes x (n_nodes-1)/2), and Spearman correlations were computed between the corresponding edges, yielding one coupling coefficient for each matrix pair. Coupling analyses involving morphometric similarity were performed only in participants with complete structural and functional acquisitions (*n* = 54), whereas coupling between functional metrics (SpiFC-SpiDyn) used the 67 participants. Next, the age-related change was investigated using OLS regression models for each pairwise region combination, with coupling strength as the dependent variable, age as a fixed effect, and sex as a covariate. Finally, to determine whether spinal region–region connection or similarities exhibit corresponding age-related changes across microstructure, SpiFC, and SpiDyn, we computed Spearman correlations between pairwise regional *t* values derived from the OLS age-effect models. In these analyses, each data point represents a pairwise region combination at the group level (i.e., the average or age-related *t* value).

#### Statistical analyses

Across all analyses, age was modeled as a continuous linear predictor. Separate regression models were applied to structural metrics, FC, and intrinsic spinal dynamics metrics, at both the spinal cord and brain levels. Sex was included as a covariate in all models. When participants contributed multiple observations to a given analysis, participant identity was included as a random effect, resulting in two types of models: ordinary least squares (OLS) regression with age and sex as fixed effects, and linear mixed-effects models (LMMs) with age and sex as fixed effects and participant as a random effect. When multiple models were applied across different regions or metrics, the resulting statistical tests were corrected for multiple comparisons using the false discovery rate (FDR) procedure. A summary of age terms, covariates, modeling choices, and multiple-comparison corrections procedure for each analysis is provided in Supplementary Table S2.

We performed bootstrap resampling with 1000 iterations for all OLS and LMM analyses to assess the stability of the results. This non-parametric method generates new datasets by randomly sampling observations from the original dataset with replacement, so that some data points may appear multiple times while others may be omitted in each resampled dataset. These resampled datasets are then used to create an empirical distribution of the model’s beta values. For each model, the resulting bootstrap distributions were summarized using the median beta values and 95% confidence intervals.

### Reporting summary

Further information on research design is available in the Nature Portfolio Reporting Summary linked to this article.

## Supplementary information

Supplementary Information

Reporting Summary

Transparent Peer Review file

## Source data

Source Data

## Data Availability

The data generated in this study are available under restricted access as they are part of an ongoing study. The complete dataset is available upon reasonable request by contacting the corresponding author. Data from one representative participant are available at 10.18112/openneuro.ds007313.v1.0.0, and functional data from the 32 younger participants are also publicly available^91^ at 10.18112/openneuro.ds005075.v1.0.1.  Source data are provided with this paper.

## Code availability

The code used for data preprocessing and analysis is available for public use here^79^: https://github.com/CarolineLndl/Landelle_spinebrain_aging.
