# Supplementary material for: Spinal cord structural and functional architecture and its shared organization with the brain across the adult lifespan
Source: Nat Commun. 2026 Apr 16;17:5269. doi: 10.1038/s41467-026-71963-2 (PMC13265777; doi:10.1038/s41467-026-71963-2)
Supplement: Supplementary file 1 — Supplementary Information [file 41467_2026_71963_MOESM1_ESM.pdf]

Supplementary figures

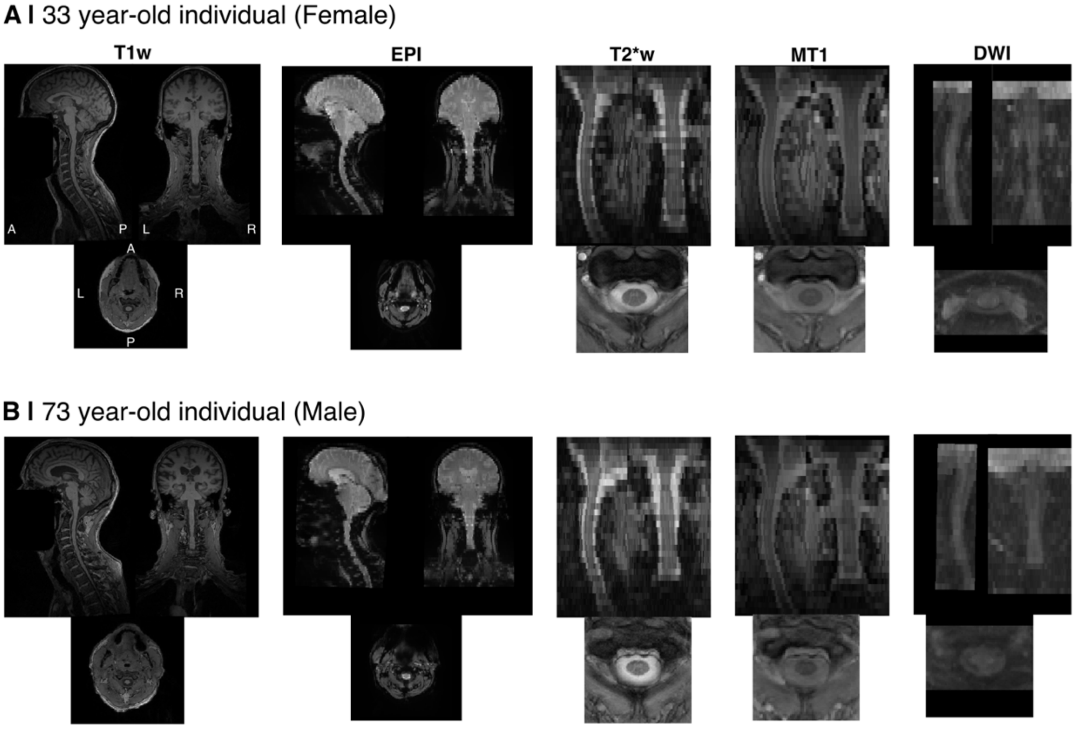

Fig. S1 | Representative individuals. Example of raw images for T1-weighted (T1w), T2*-weighted (T2*w), Magnetization transfer (MT1), and mean images for EPI, Diffusion weighted images (DWI) of two participants: (A) 33 year-old female, (B) 73 year-old male)

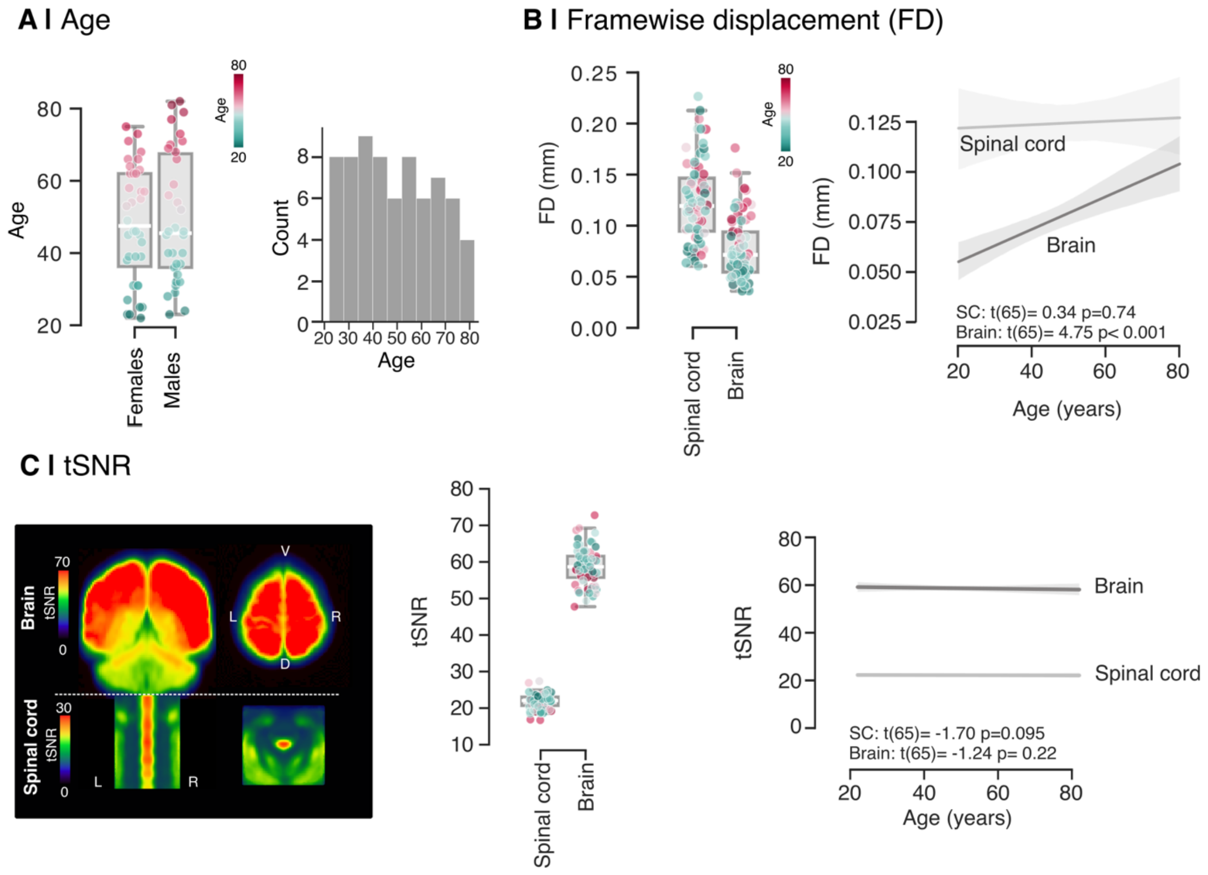

Figure S2 | Age distribution and quality check metrics. A. Left: Distribution of participant ages by sex. Right: Histogram of age distribution (n=67). **B.** Framewise displacement (FD, n=67). Left: boxplots of FD for spinal cord and brain masks. Each box extends from the 25th to the 75th percentile of the group's distribution, and the medians are represented by the horizontal line inside the box. The vertical extending lines denote the extreme values within 1.5 interquartile ranges. Individual data points are colored by participant age from green (younger) to pink (older). Right: Linear regressions between age and FD for the spinal cord and the brain (right), solid line represents the linear regression fit and the shaded gray area indicated the 95% confidence interval **C.** Temporal signal-to-noise ratio (tSNR, n=67). Left: Average tSNR maps for the brain and the spinal cord, displayed with distinct color scales for optimal visualization. Middle: boxplots of tSNR for the spinal cord and brain masks. Each box extends from the 25th to the 75th percentile of the group's distribution, and the medians are represented by the horizontal line inside the box. Individual data points are colored by participant age from green (younger) to pink (older). Right: Linear regressions between age and tSNR for the spinal cord and the brain), solid line represents the linear regression fit and the shaded gray area indicated the 95% confidence interval. D: dorsal; V: ventral; L: left; R: right.

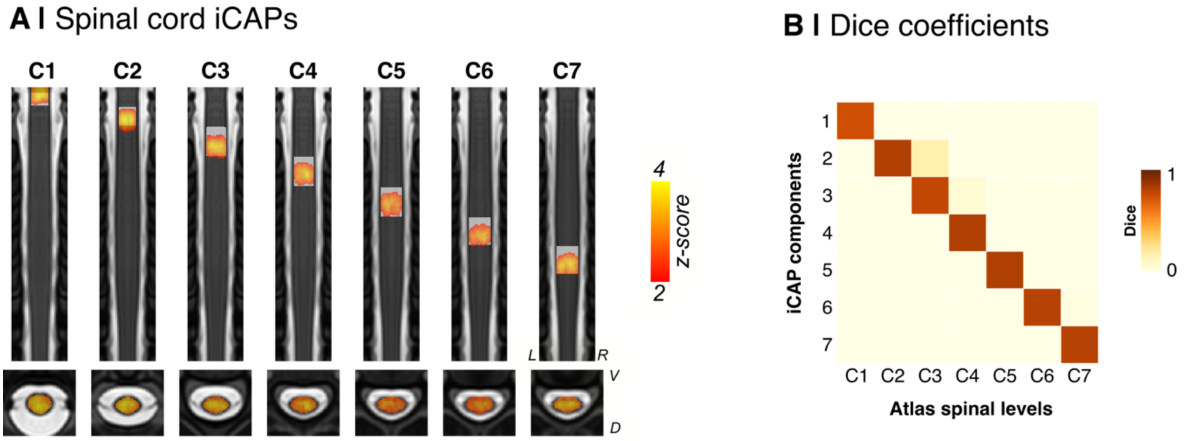

Figure S3 | Spinal cord segmental levels. A. iCAPs were computed on the 67 participants. Spinal cord iCAPs presented in rostro-caudal order. The labels of the iCAPs (from C1 to C7) are determined based on the spinal segmental atlas (Frostell et al., 2016), plotted as a white underlay. L: left; R: right; V: ventral; D: dorsal. **B.** The similarity between the iCAP components and the atlas-based spinal levels was evaluated using Dice coefficients between the two maps. iCAPs : innovation-driven co-analysis Patterns,

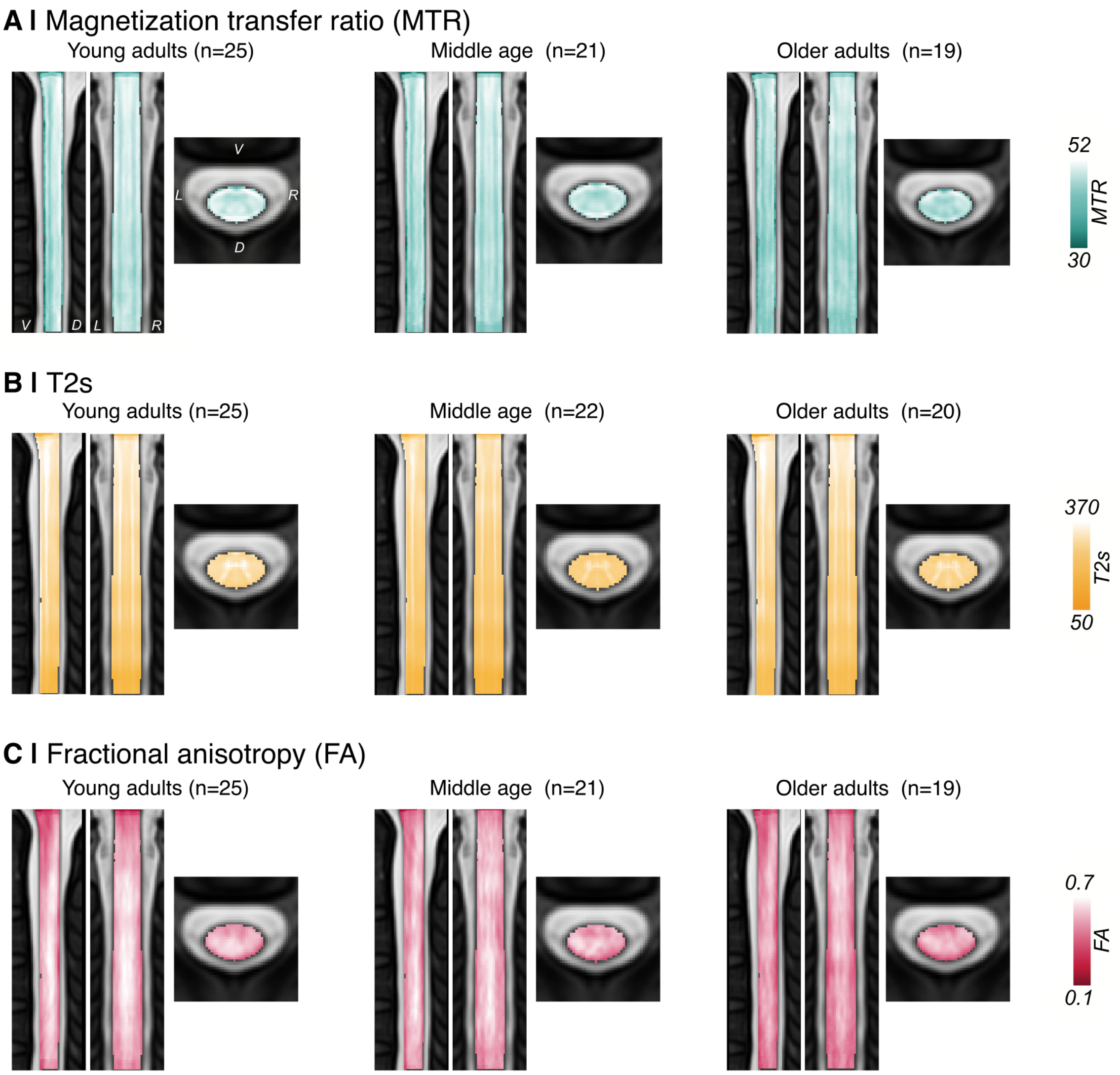

Figure S4 | Average metric maps. We computed the average metric maps for MTR (A), T2s (B) and FA (C) metrics by dividing into three age groups: young adults (20-40 years), middle-aged adults (40-60 years) and older adults (60-80 years). L: left; R: right; V: ventral; D: dorsal, MTR: Magnetization transfer ratio, FA: Fractional anisotropy.

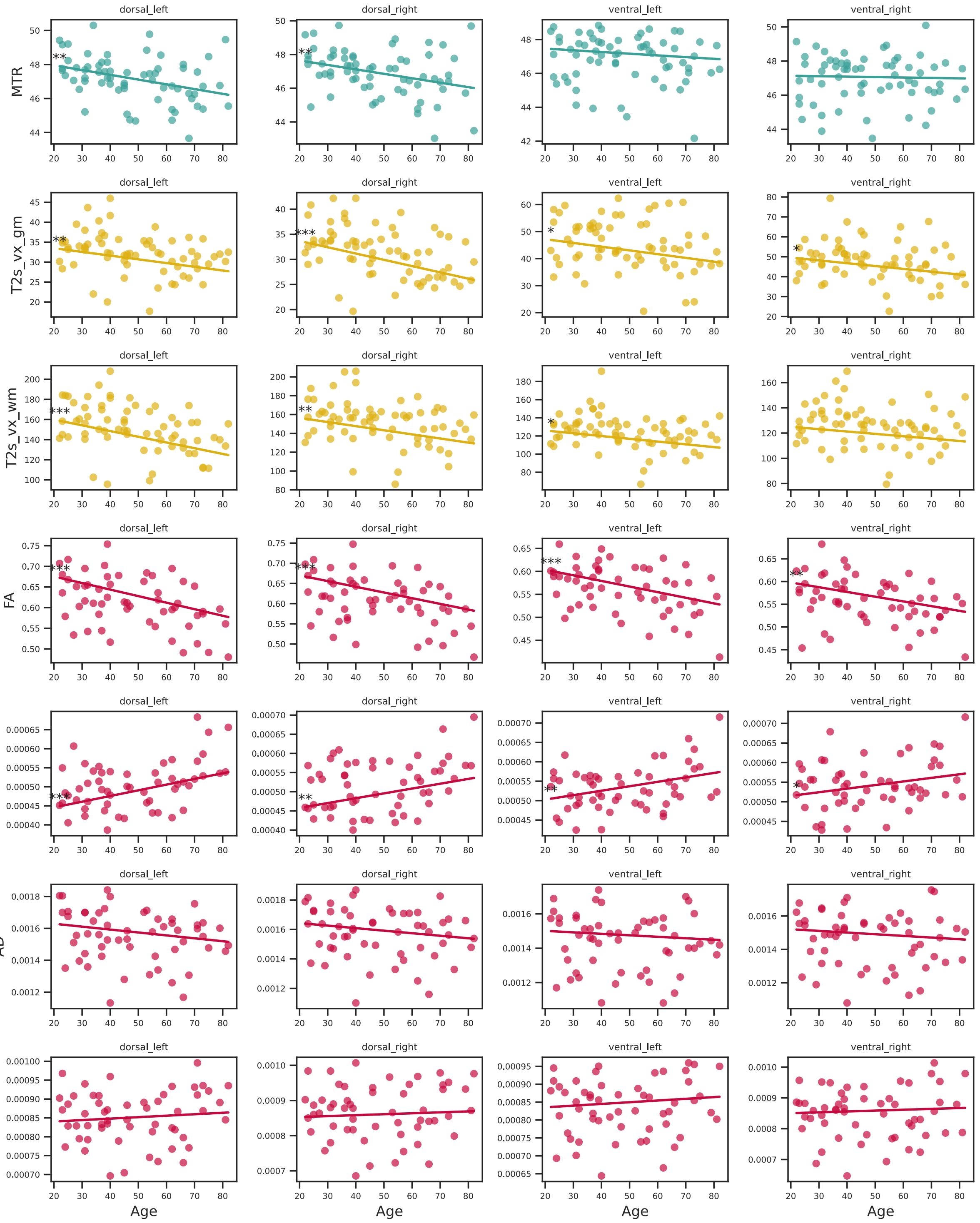

Fig S5 | Microstructural changes in the spinal cord. Magnetization transfer ratio (MTR, n=55), GM voxel counts (T2s_vx_gm, n=67), WM voxel counts (T2s_vx_wm, n=67), and several diffusion metrics (n=55) were extracted within four spinal cord quadrants. Each point represents the mean value per subject for a given ROI across spinal levels, and lines show the predicted trend from linear mixed-effects models controlling for sex as a covariate. Random intercepts account for repeated measures within subjects. FA: fractional anisotropy; RD: radial diffusivity; MD: mean diffusivity; AD: axial diffusivity. Asterisks indicate significance of the age effect: *p < 0.05; **p < 0.01; ***p < 0.001 (uncorrected, see Table S3 for FDR-corrected values).

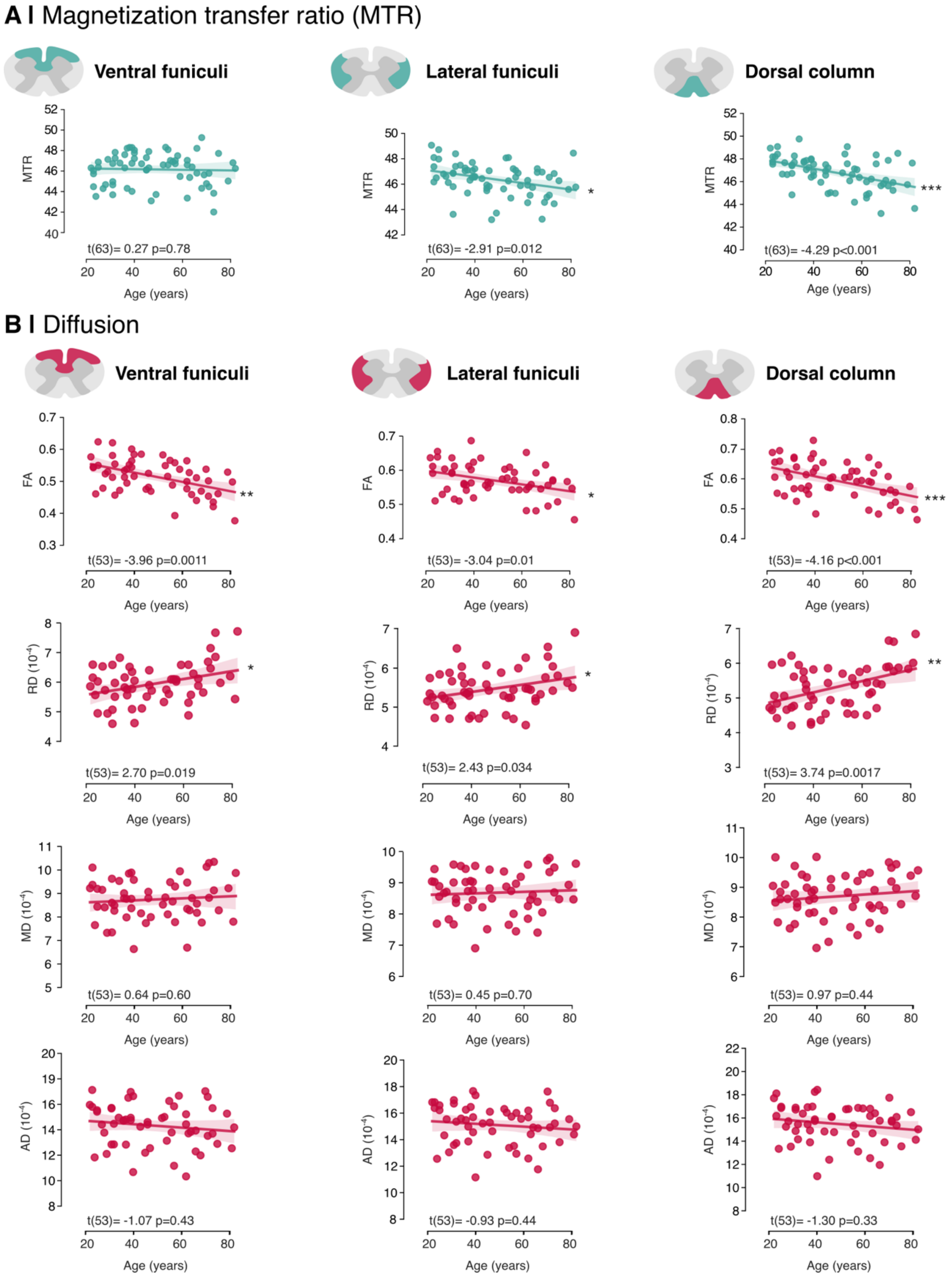

Fig S6 | Microstructural changes in the spinal cord white matter (WM) tracts. Magnetization transfer ratio (MTR, n=55, **A**) and several diffusion metrics (n=54, **B**) were extracted within three white matter tracts (dorsal column, lateral funiculi and ventral funiculi). Linear regressions between age and each metric were computed. t-values and degree of freedom (t(df)) as well as FDR-corrected p-values for the age effect are reported on each graph). Solid line represents the linear regression fit and the shaded area indicated the 95% confidence interval. FA: fractional anisotropy, RD: radial diffusivity, MD: mean diffusivity, AD: axial diffusivity. P-values are corrected for multiple comparison *: $p < 0.05$; ***: $p < 0.01$; ***: $p < 0.001$;

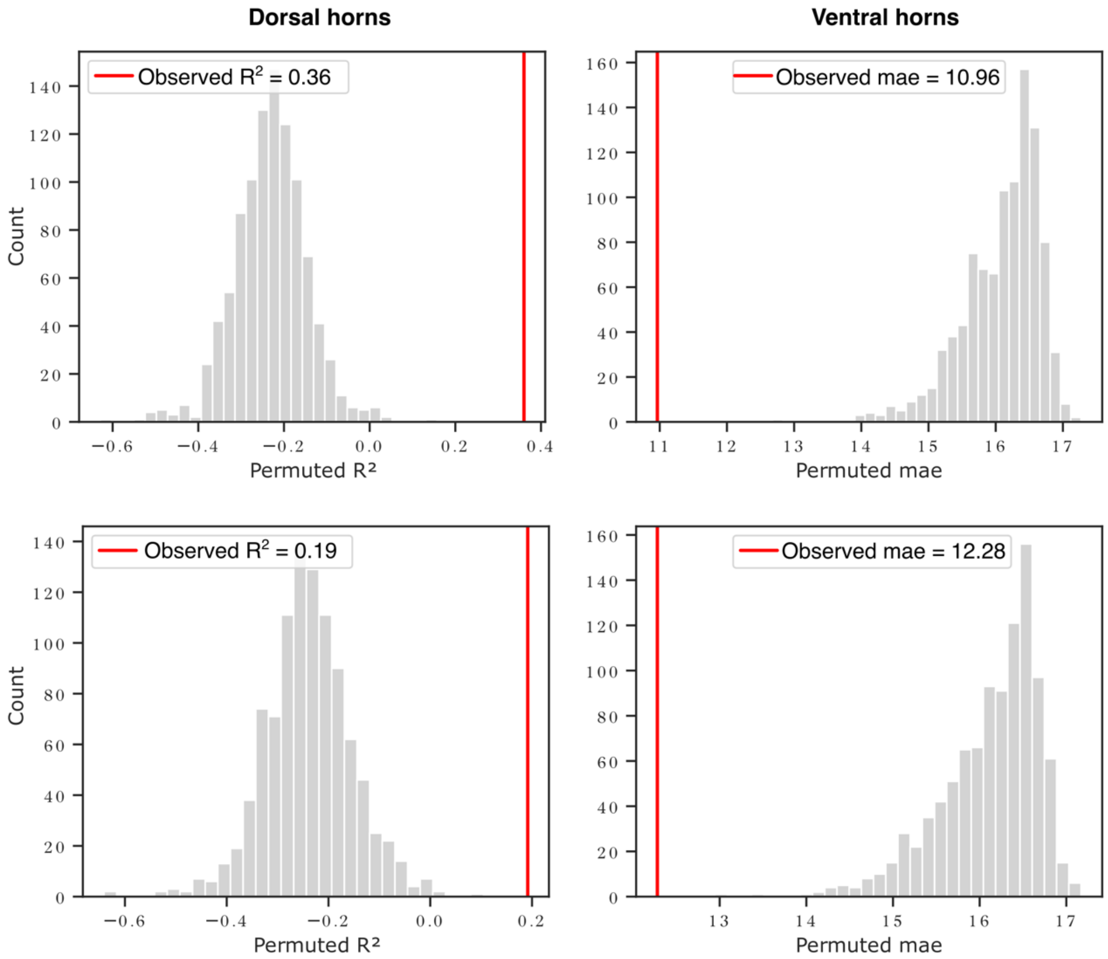

Fig S7 | Permutation tests on age prediction models. The permutation procedure consisted of randomly shuffling participants' age labels 1000 times and refitting the ElasticNet prediction models for each permutation, generating null distributions of R^2 and MAE values. This was performed separately for models applied to the dorsal and ventral horns. Gray bars represent the distribution of R^2 (left) or MAE (right) values across the 1000 iterations, and the red vertical line indicates the observed value. The observed model performance for both dorsal and ventral ROIs exceeded the values expected by chance ($p < 0.001$). MAE: Mean Absolute Error; R^2 : proportion of variance in chronological age explained by the model predictions; ROI: Region of Interest.

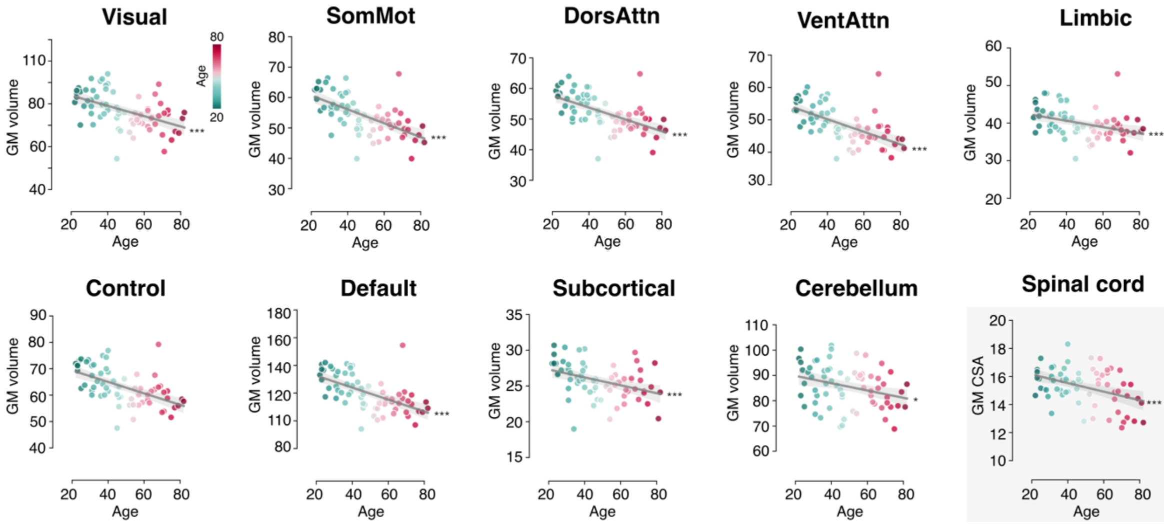

Fig S8 | Structural changes in the gray matter (GM). GM volume was extracted across seven cortical networks, as well as the cerebellum and subcortical regions. For the spinal cord, cross-sectional area (CSA) was measured. Linear regressions between age and each metric were computed. Age effect: *: $p_{FDR} < 0.05$; ***: $p_{FDR} < 0.01$; ***: $p_{FDR} < 0.001$.

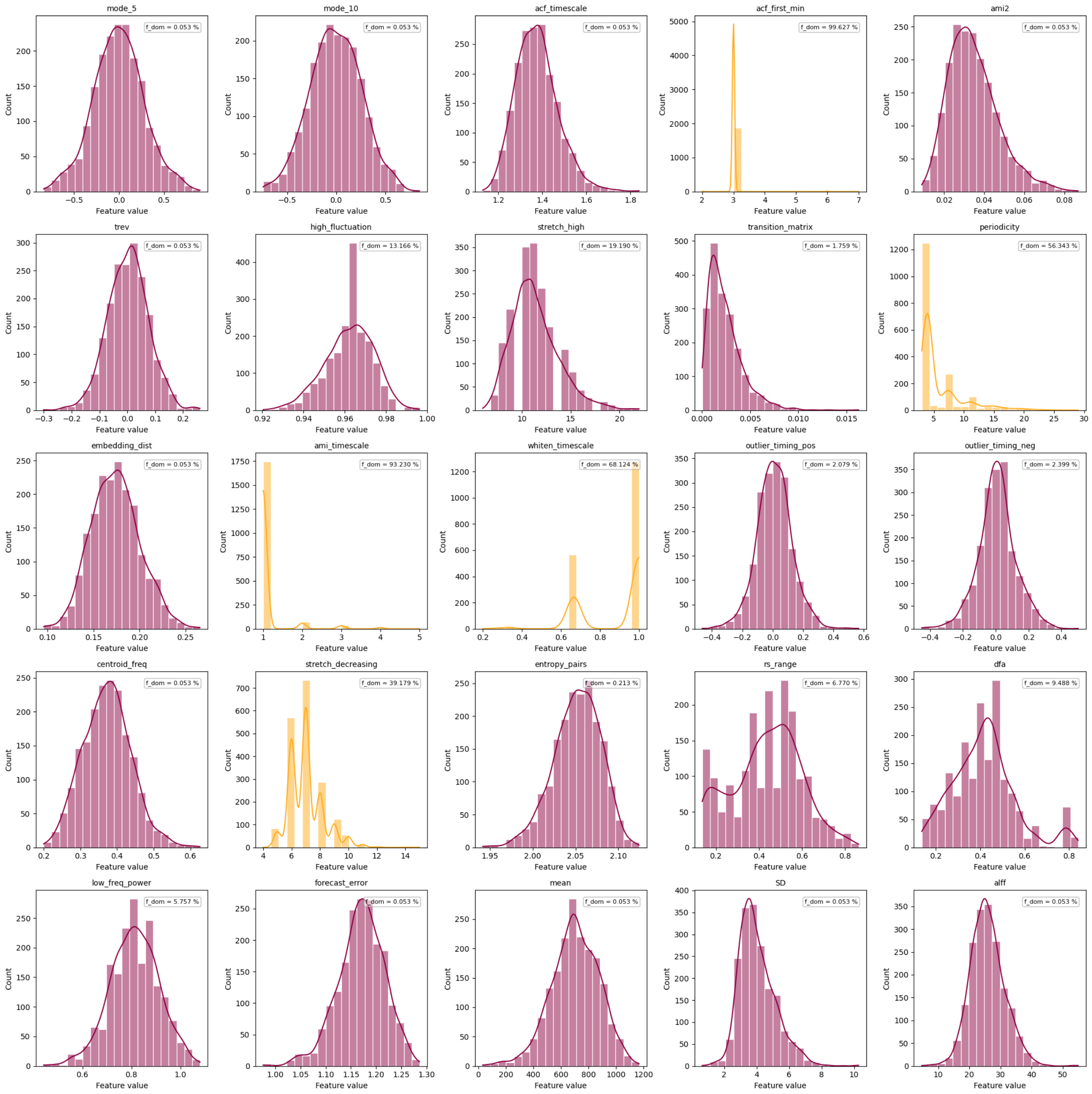

Figure S9 | Distribution of the SpiDyn features. Distribution of each feature, with pink indicating the features retained for further analyses and yellow indicating features excluded due to a dominant value and inherent lack of variability. The proportion of dominant value is reported on each graph as ‘f_dom’.

Supplementary tables

Contrasts	# Participants (#Females/ #Males)	Age
fMRI/ T1w /T2s	67 (36/31)	48.67 ± 17.18
MTR	65 (35/30)	48.28 ± 17.08
DWI	55 (29/26)	48.29 ± 17.58
All	54 (28/26)	48.33 ± 17.74

Table S1 | Demographic population. The number of participants, mean ± standard deviation, as well as the number of females and males, are provided for each contrast after participant exclusions. The last row evaluates the population across the different contrasts.

	Test	X	Y	Cov	Random	Model	Multiple correction
**Spinal cord**							
**Morpho**	Intra vs. inter Fig. 1C	Intra Similarity	Inter Similarity	-	-	Paired t-test	-
	Age-effect Fig. 1D Fig. S5	Age	Morpho. metric	Sex	IDs	LMM	FDR (4 rois x 7 metrics)
	Age-effect Fig S6	Age	Morpho. metric	Sex	-	OLS	FDR (3 tracts x 5 metrics)
**FC**	Intra vs. inter Fig 2B	Intra correlation	Inter correlation	-	-	Paired t-test	-
	Age-effect Fig 2D /Table S4	Age	Inter Correlation	Sex	IDs	LMM	FDR (6 types of connexions)
	Age-effect Table S5	Age	Intra Correlation	Sex	IDs	LMM	FDR (6 types of connexions)
**Dyn**	Intra vs. inter Fig 2B	Intra Similarity	Inter Similarity	-	-	Paired t-test	-
	Age-effect Fig 2E / Table S6	Age	SpiDyn metric	Sex	IDs	LMM	FDR (4 rois * 20 metrics)
**All**	Coupling, Fig 2C, 3BE	Similarity/ FC	Similarity/ FC	-	-	Spearman correlation	-
	Age-effect in coupling Fig 2F, 3CF	t-values (Similarity or FC)	t-values (Similarity or FC)	-	-	Spearman correlation	-
**Brain – spinal cord**							
**Morpho**	Age-effect Fig. S8 /Table S7	Age	GM csa or GM volume	Sex	-	OLS	FDR (10 networks)
	csa vs. brain Fig 4A	Age * GM volume	GM csa	Sex	-	OLS	-
**FC**	Intra vs. inter Table S8	Intra correlation	Inter correlation	-	-	Paired t-test	-
	Inter vs. 0 Table S9	Intra correlation	0	-	-	t-test	-
	Inter vs. 0 Table S9	Inter correlation	0	-	-	t-test	-
	Age effect Fig 4D /Tab S10	Age	Inter Correlation	Sex	IDs	LMM	FDR (10 networks)
	Age effect Fig 4D /Tab S11	Age	Intra Correlation	Sex	IDs	LMM	FDR (10 networks)
**Dyn**	Age-effect Fig 4F/Table S12	Age	SpiDyn metric	Sex	-	LMM	FDR (11 networks)

Table S2. Summary of statistical analyses. For each morphometric (morpho), functional connectivity (FC) or functional dynamics analyses (Dyn). The table lists the type of test, independent variable (X), dependent variable (Y), covariates included, random effects (if applicable), statistical model used, and any multiple comparison corrections applied. IDs: participants; LMM: Linear mixed model; OLS: Ordinary least squares; FDR: False discovery rate; GM: Gray matter

Features	ROI	Predictor	Beta	95% Bootstrap CI	t-value	p-value	p-value FDR	Sign.
MTR (n=55)	DL	age	-0.028	[-0.044 -0.013]	-3.00	0.0027	0.011	*
		sex	-0.0020	[-0.49 0.49]	-0.0064	0.99	0.99	ns
	DR	age	-0.026	[-0.045 -0.0073]	-2.69	0.0071	0.016	*
		sex	-0.030	[-0.47 0.58]	0.089	0.92	0.96	ns
	VL	age	-0.010	[-0.024 0.0056]	-1.12	0.26	0.35	ns
		sex	-0.71	[-1.24 -0.15]	-2.31	0.021	0.058	.
	VR	age	-0.0025	[-0.018 0.013]	-0.24	0.80	0.80	ns
		sex	-0.26	[-0.80 0.26]	-0.77	0.44	0.56	ns
GM (n=67)	DL	age	-0.093	[-0.14 -0.049]	-2.61	0.0090	0.019	*
		sex	2.82	[0.82 4.77]	2.30	0.020	0.058	.
	DR	age	-0.12	[-0.17 -0.077]	-4.09	<0.001	0.0012	**
		sex	3.050	[1.44 4.67]	2.92	0.0035	0.014	*
	VL	age	-0.14	[-0.25 -0.044]	-2.17	0.030	0.055	.
		sex	4.052	[0.58 7.076]	1.85	0.063	0.131	ns
	VR	age	-0.14	[-0.25 -0.048]	-2.14	0.032	0.056	.
		sex	4.82	[1.31 8.56]	2.12	0.033	0.079	.
WM (n=67)	DL	age	-0.57	[-0.79 -0.35]	-3.92	<0.001	0.0012	**
		sex	16.092	[8.85 24.02]	3.24	0.0011	0.0090	**
	DR	age	-0.44	[-0.65 -0.21]	-2.96	0.0030	0.011	*
		sex	16.41	[8.43 24.35]	3.24	0.0012	0.0090	**
	VL	age	-0.30	[-0.49 -0.15]	-2.57	0.010	0.0120	*
		sex	14.58	[7.83 20.90]	3.58	<0.001	0.0089	**
	VR	age	-0.188	[-0.36, 0.021]	-1.61	0.11	0.17	ns
		sex	12.06	[5.85 18.07]	3.02	0.0024	0.014	*
FA (n=55)	DL	age	-0.0016	[-0.0023 -0.0009]	-3.80	<0.001	0.0013	**
		sex	-0.037	[-0.059 -0.016]	-2.52	0.011	0.040	*
	DR	age	-0.0014	[-0.0020 -0.00075]	-3.51	<0.001	0.0031	**
		sex	-0.045	[-0.067 -0.024]	-3.22	0.0012	0.0090	**
	VL	age	-0.0012	[-0.0018 -0.0005]	-3.34	<0.001	0.0040	**
		sex	-0.016	[-0.036 0.0044]	-1.23	0.22	0.32	ns
	VR	age	-0.0010	[-0.0017 -0.0004]	-2.85	0.0043	0.014	*
		sex	-0.021	[-0.040 -0.0003]	-1.61	0.11	0.20	ns
RD (n=55)	DL	age	1.50e-6	[6.75e-7 2.24e-6]	3.46	<0.001	0.0031	**
		sex	3.20e-5	[7.21e-6 5.45e-5]	2.12	0.033	0.079	ns
	DR	age	1.26e-6	[5.27e-7 1.97e-6]	2.76	0.0056	0.016	*
		sex	4.70e-5	[2.21e-5 7.37e-5]	2.97	0.0030	0.014	*

	VL	age	1.14e-6	[3.93e-7 1.85e-6]	2.72	0.0065	0.016	*
		sex	-4.00e-6	[-2.80e-5 1.78e-5]	-0.27	0.78	0.88	ns
	VR	age	9.17e-7	[3.70e-7 1.72e-6]	1.96	0.049	0.081	.
		sex	6.00e-6	[-2.00e-5 3.30e-5]	0.39	0.70	0.91	ns
AD (n=55)	DL	age	-1.83e-6	[-3.67e-6 -1.65e7]	-1.49	0.14	0.20	ns
		sex	-5.81e-5	[-1.21e-4 6.99e6]	-1.35	0.17	0.27	ns
	DR	age	-1.72e-6	[-3.68e-6 1.71e7	-1.31	0.19	0.26	ns
		sex	-5.07e-5	[-1.20e-4 -1.81e-5]	-1.11	0.26	0.37	ns
	VL	age	-8.60e-7	[-2.67e-6 8.81e-7]	-0.68	0.50	0.55	ns
		sex	-8.11e-5	[-1.49e-4 -1.64e-5]	-1.83	0.065	0.13	ns
	VR	age	-1.00e-6	[-3.11e-6 8.10e-7]	-0.80	0.42	0.53	ns
		sex	-6.87e-5	[-1.37e-4 -4.94e6]	-1.58	0.11	0.20	ns
MD (n=55)	DL	age	3.88e-7	[-5.36e-7 1.14e-6]	0.72	0.47	0.54	ns
		sex	1.98e-6	[-2.83e-5 3.02e-5]	0.10	0.91	0.96	ns
	DR	age	2.67e-7	[-6.38e-7 1.07e-6]	0.44	0.65	0.68	ns
		sex	1.45e-5	[-1.91e-6 4.88e-5]	0.69	0.49	0.59	ns
	VL	age	4.76e-7	[-5.19e-7 1.48e-6]	0.78	0.43	0.53	ns
		sex	-2.97e-5	[-6.35e-5 4.23e-6]	1.40	0.16	0.26	.
	VR	age	2.78e-7	[-7.74e-7 1.25e-6]	0.44	0.66	0.68	ns
		sex	-1.87e-5	[-5.18e-5 1.50-5]	-0.84	0.40	0.53	ns

Table S3 | Age and sex effects on individual morphometric features. The age-related effect was obtained using a linear mixed model including age and sex as fixed effects (predictors) and participants as random effects. The significance of each fixed effect of each model (each feature and each quadrant) was assessed using a two-sided t-test. The number of participants included in each model is reported in the first column (n). The coefficient of regression (Beta), the 95% boostraps confidence interval, the t-values, p-values and FDR-corrected p-values are reported for each fixed effect. For the sex effect, Females is the reference level. The coefficient (and its t-value) represents the extent to which the mean outcome for Males differs from that for Females; thus, a positive value indicates higher values in Males *compared to* Females, and *vice versa*. The last column represents the significance: ns: not significant, . : significant before FDR but not after, *: $p_{FDR} < 0.05$, **: $p_{FDR} < 0.01$; ***: $p_{FDR} < 0.001$. CI: confidence interval.

ROI-pairs	Predictor	Beta	95% Bootstrap CI	t-value	p-value	p-value FDR	Sign.
**Dorso-Dorsal (DL-DR)**	age	0.00031	[-1.60e-5 6.50e-4]	1.62	0.10	0.10	ns
	sex	-0.018	[-0.027 -0.0073]	-2.71	0.0067	0.040	*
**Ventro-Ventral (VL-VR)**	age	0.00047	[1.7e-4 7.59e-4]	2.44	0.015	0.018	*
	sex	-0.0082	[-0.017 0.0016]	-1.25	0.21	0.32	ns
**R Dorso-Ventral (DR-VR)**	age	0.00051	[2.3e-4 8.3e-4]	2.71	0.0068	0.014	*
	sex	-0.0056	[-0.016 0.0041]	-0.87	0.38	0.40	ns
**L Dorso-Ventral (DL-VL)**	age	0.00050	[1.93e-4 8.1e-4]	2.56	0.010	0.016	*
	sex	-0.0056	[-0.016 0.0038]	-0.84	0.40	0.40	ns
**L Cross (DL-VR)**	age	0.00062	[3.42e-4 8.82e-4]	3.62	2.90^{e-4}	0.0017	**
	sex	-0.0074	[-0.017 0.0015]	-1.26	0.21	0.32	ns
**R Cross (DR-VL)**	age	0.00056	[2.67e-4 8.9e-4]	2.94	0.0032	0.0096	**
	sex	-0.012	[-0.023 -0.0020]	-1.91	0.056	0.17	ns

Table S4 | Age and sex effects on inter-segmental functional connectivity. The age-related effect was obtained using mixed linear models including age and sex as fixed effects (predictors) and participants as random effects. The fixed effects of each model (each pair of ROIs between distinct segments) were assessed using a two-sided t-test. The degree of freedom was always 65 (*i.e.*, 67 individuals – 2 fixed factors) and the coefficient of regression (Beta), the 95% boostraps confidence interval, t-values, p-values and FDR-corrected p-values are reported for each predictor. For the sex effect, Females is the reference level. The coefficient (and its t-value) represents the extent to which the mean outcome for Males differs from that for Females; thus, a positive value indicates higher values in Males *compared to* Females, and *vice versa*. The last column represents the significance: ns: not significant, . : significant before FDR but not after, *: $p_{FDR} < 0.05$, **: $p_{FDR} < 0.01$; ***: $p_{FDR} < 0.001$. CI: confidence interval. DL: dorsal left, DR: dorsal right, VL: ventral left, VR: ventral right.

ROI-pairs	Predictor	Beta	95% Bootstrap CI	t-value	p-value	p-value FDR	Sign.
**Dorso-Dorsal (DL-DR)**	age	-8.76e-05	[-0.0013 0.0013]	-0.10	0.92	0.92	ns
	sex	-0.061	[-0.10 -0.017]	-2.048	0.040	0.048	*
**Ventro-Ventral (VL-VR)**	age	-0.0014	[-0.0025 -0.00023]	-1.80	0.072	0.14	ns
	sex	-0.10	[-0.15 -0.062]	-3.89	1.01e-4	1.01e-4	***
**R Dorso-Ventral (DR-VR)**	age	0.00083	[5.70e-05 1.70e-03]	1.57	0.11	0.17	ns
	sex	-0.032	[-0.061 -0.0041]	-1.79	0.072	0.072	ns
**L Dorso-Ventral (DL-VL)**	age	0.0013	[0.00073 0.0023]	2.71	0.0065	0.039	*
	sex	-0.044	[-0.072 -0.014]	-2.36	0.018	0.027	*
**L Cross (DL-VR)**	age	0.00079	[-6.88e-05 1.58e-03]	2.26	0.023	0.069	.
	sex	-0.044	[-0.071 -0.015]	-2.72	0.0064	0.019	*
**R Cross (DR-VL)**	age	0.0011	[0.00032 0.0018]	1.44	0.15	0.18	ns
	sex	-0.045	[-0.071 -0.020]	-2.39	0.017	0.072	.

Table S5 | Age and sex effects on intra-segmental functional connectivity. The age-related effect was obtained using mixed linear models including age and sex as fixed effects (predictors) and participants as random effects. The fixed effects of each model (each pair of rois within the same segment) were assessed using a two-sided t-test. The degree of freedom was always 65 (i.e., 67 individuals – 2 fixed factors) and the coefficient of regression (Beta), the 95% boostraps confidence interval, the t-values, p-values and FDR-corrected p-values are reported for each predictor. For the sex effect, Females is the reference level. The coefficient (and its t-value) represents the extent to which the mean outcome for Males differs from that for Females; thus, a positive value indicates higher values in Males compared to Females, and *vice versa*. The last column represents the significance: ns: not significant, . : significant before FDR but not after, *: $p_{FDR} < 0.05$, **: $p_{FDR} < 0.01$; CI: confidence interval; ***: $p_{FDR} < 0.001$. DL: dorsal left, DR: dorsal right, VL: ventral left, VR: ventral right

Feature	Rois	Pred.	Beta	95% Bootstrap CI	t-value	p-value	p-value FDR	Sign.
**SD**	**DL**	age	0.016	[0.010 0.021]	3.71	2.05^{e-4}	0.016	*
		sex	0.21	[0.0047 0.42]	1.44	0.15	0.74	ns
	**DR**	age	0.013	[0.0068 0.019]	3.20	0.0013	0.035	*
		sex	0.24	[0.023 0.44]	1.72	0.85	0.64	ns
	**VL**	age	0.0080	[0.0017 0.014]	2.11	0.035	0.24	.
		sex	0.039	[-0.16 0.22]	0.30	0.76	0.99	ns
	**VR**	age	0.0037	[-0.0022 0.0099]	0.80	0.42	0.73	ns
		sex	0.0054	[-0.19 0.18]	0.033	0.97	0.99	ns
**ACF timescale**	**DL**	age	9.61e-4	[3.72e-4 1.54e-3]	3.12	0.0018	0.036	*
		sex	-8.87e-3	[-0.027 0.0084]	-0.84	0.40	0.89	ns
	**DR**	age	5.36e-4	[5.42e-5 1.05e-3]	1.72	0.085	0.42	ns
		sex	9.92e-3	[-0.0066 0.026]	0.93	0.35	0.89	ns
	**VL**	age	5.3e-4	[6.82e-5 1.04e-3]	1.65	0.099	0.42	ns
		sex	8.44e-4	[-0.016 0.018]	0.077	0.93	0.99	ns
	**VR**	age	4.14e-4	[3.44e-5 8.36e-4]	1.60	0.11	0.42	ns
		sex	-0.014	[-0.028 0.00016]	-1.60	0.11	0.63	ns
**ALFF**	**DL**	age	0.066	[0.033 0.099]	2.90	0.0037	0.042	*
		sex	3.47	[2.28 4.63]	4.47	7.78^{e-6}	6.30^{e-4}	***
	**DR**	age	0.060	[0.026 0.093]	2.70	0.0070	0.071	.
		sex	3.070	[1.94 4.25]	4.04	5.32^{e-5}	0.002	**
	**VL**	age	0.023	[-0.012 0.061]	1.036	0.29	0.66	ns
		sex	2.31	[1.090 3.44]	3.02	0.0025	0.068	.
	**VR**	age	0.013	[-0.021 0.045]	0.58	0.56	0.77	ns
		sex	2.14	[0.97 3.24]	2.89	0.0038	0.077	.
**Centroid frequency**	**DL**	age	0.00075	[3.04e-4 0.0017]	3.34	8.45^{e-4}	0.034	*
		sex	-0.0026	[-0.015 0.0099]	-0.34	0.73	0.99	ns
	**DR**	age	0.00022	[-9.66e-5 6.09e-4]	1.01	0.31	0.65	ns
		sex	0.0086	[-0.0023 0.020]	1.15	0.25	0.82	ns
	**VL**	age	0.0003	[2.74e-5 6.47e-4]	1.49	0.13	0.43	ns
		sex	-1.97e-4	[-0.012 0.012]	-0.025	0.97	0.99	ns
	**VR**	age	0.00032-	[1.14e-5 5.86e-4]	1.57	0.11	0.42	ns
		sex	0.011	[-0.022 -0.00034]	-1.55	0.12	0.64	ns
**Embedding distance**	**DL**	age	1.49e-4	[2.84e-5 2.83e-4]	2.07	0.038	0.24	.
		sex	-0.0017	[-0.0060 0.0023]	-0.69	0.49	0.95	ns
	**DR**	age	9.80	[-3.27e-5 2.22e-4]	1.37	0.16	0.48	ns
		sex	0.0012	[-0.0032 0.0055]	0.48	0.63	0.87	ns
	**VL**	age	9.24e-5	[-4.16e-5 2.52e-4]	1.07	0.28	0.66	ns
		sex	0.0011	[-0.0055 0.0034]	-0.37	0.71	0.99	ns
	**VR**	age	0.00014	[5.09e-5 2.51e-4]	2.01	0.04	0.25	.
		sex	0.0066	[-0.010 -0.0029]	-2.64	0.008	0.13	.
**Entropy pairs**	**DL**	age	-1.79e-4	[-3.34e-4 -2.71e-5]	-2.21	0.027	0.21	.
		sex	0.0024	[-0.0018 0.0070]	0.85	0.39	0.89	ns

	**DR**	age	-6.26e-5	[-2.20e-4 8.68e-5]	-0.75	0.45	0.72	ns
		sex	-0.0036	[-0.0083 9.1-e-4]	-1.26	0.21	0.82	ns
	**VL**	age	-6.27e-5	[-1.96e-4 7.38e-5]	-0.82	0.41	0.73	ns
		sex	3.17e-4	[-0.0041 0.0050]	-0.12	0.90	0.99	ns
	**VR**	age	-5.73e-6	[-0.00013 0.00010]	-0.077	0.94	0.95	ns
		sex	0.0045	[0.00068 0.0084]	-1.77	0.077	0.64	ns
**Low frequency power**	**DL**	age	-9.95e-4	[-0.0016 -0.00033]	-2.94	0.0032	0.042	*
		sex	0.0033	[-0.015 0.024]	0.28	0.77	0.99	ns
	**DR**	age	-5.05e-4	[-9.94e-4 -4.07e-5]	-1.68	0.09	0.42	ns
		sex	-0.017	[-0.034 -0.0011]	-1.63	0.10	0.63	ns
	**VL**	age	-4.14e-4	[-9.10e-4 5.51e-5]	-1.28	0.20	0.53	ns
		sex	-6.10e-4	[-0.022 0.011]	-0.55	0.58	0.97	ns
	**VR**	age	-1.90e-5	[-2.72e-5 7.14e-5]	0.58	0.56	0.90	ns
		sex	-1.64e-4	[-0.0016 0.0018]	0.15	0.88	0.99	ns
**Forecast error**	**DL**	age	-5.87e-4	[-9.28e-4 -2.76e-4]	-3.058	0.0022	0.035	*
		sex	0.0023	[-0.0067 0.011]	0.35	0.72	0.99	ns
	**DR**	age	-3.29e-4	[-5.81e-4 -9.43e-5]	-2.066	0.039	0.23	.
		sex	-0.0028	[-0.011 0.064]	-0.50	0.61	0.97	ns
	**VL**	age	-2.90e-4	[-5.47e-4 -5.28e-5]	-1.75	0.079	0.42	ns
		sex	3.00e-4	[-0.0089 0.0083]	-0.053	0.96	0.99	ns
	**VR**	age	-3.16e-4	[-5.32e-4 -1.12e-4]	-2.38	0.017	0.15	.
		sex	0.0053	[-0.0022 0.012]	1.10	0.27	0.82	ns

Table S6 | Age and sex effects on functional dynamic profiles. The age-related effect was obtained for each feature and four regions using a linear mixed model including age and sex as fixed effects and participants as random effects. The fixed effects were assessed using a two-sided t-test. We report the model result for the features with a significant age effect before FDR correction for at least one region. The degree of freedom was always 65 (*i.e.,* 67 individuals – 2 fixed factors) and the coefficient of regression (Beta), the 95% boostraps confidence interval, the t-values, p-values and FDR-corrected p-values are reported for each fixed effect. For the sex effect, Female is the reference level. The coefficient (and its t-value) represents the extent to which the mean outcome for Males differs from that for Females; thus, a positive value indicates higher values in Males compared to Females, and *vice versa*. The last column represents the significance: ns: not significant. : significant before FDR but not after, *: $p_{FDR} < 0.05$, **: $p_{FDR} < 0.01$; ***: $p_{FDR} < 0.001$. CI: confidence interval; DL: dorsal left, DR: dorsal right, VL: ventral left, VR: ventral right, SD: Standard deviation, ALFF: amplitude of low frequency fluctuation, ACF: autocorrelation function.

Networks	Predictor	Beta	95% Bootstrap CI	t-value	p-value	p-value FDR	Sign.
**Visual**	age	-0.24	[-0.33 -0.15]	-4.73	1.26^{e-05}	2.11^{e-05}	***
	sex	3.57	[0.22 7.15]	2.02	0.047	0.089	.
**SomMot**	age	-0.23	[-0.29 -0.17]	-6.49	1.41^{e-08}	1.41^{e-07}	***
	sex	2.40	[0.21 4.91]	1.97	0.052	0.089	ns
**DorsAttn**	age	-0.20	[-0.26-0.14]	-6.05	8.16^{e-08}	2.04^{e-07}	***
	sex	2.07	[-0.070 4.47]	1.88	0.064	0.092	ns
**VentAttn**	age	-0.20	[-0.25 -0.14]	-6.11	6.51^{e-08}	2.04^{e-07}	***
	sex	2.60	[0.40 5.0081]	2.36	0.021	0.070	.
**Limbic**	age	-0.087	[-0.13 -0.036]	-3.66	5.09^{e-04}	7.27^{e-04}	***
	sex	3.36	[1.88 5.014]	4.13	1.06^{e-4}	0.0011	**
**Control**	age	-0.22	[-0.28 -0.15]	-5.54	5.96^{e-07}	1.19^{e-06}	***
	sex	2.94	[0.23 5.88]	2.17	0.033	0.084	.
**Default**	age	-0.44	[-0.56 -0.30]	-6.10	6.71^{e-08}	2.04^{e-07}	***
	sex	6.59	[1.88 11.93]	2.69	0.009	0.046	*
**Subcortical**	age	-0.056	[-0.086 -0.025]	-3.50	8.54^{e-04}	9.49^{e-04}	***
	sex	0.96	[-0.17 2.086]	1.75	0.084	0.10	ns
**Cerebellum**	age	-0.15	[-0.24 -0.034]	-2.62	0.011	0.011	*
	sex	2.56	[-1.18 6.25]	1.33	0.19	0.21	ns
**Spinal cord**	age	-0.030	[-0.045 -0.015]	-3.53	7.78^{e-04}	9.94^{e-04}	***
	sex	-0.27	[-0.83 0.31]	-0.90	0.37	0.37	ns

Table S7 | Age and sex effects on brain GM volume and spinal cord CSA. The age-related effect on gray matter (GM) volume was obtained for each brain network and the GM cross-sectional areas for the spinal cord using an ordinary least squares (OLS) model including age and sex as fixed effects. The fixed effects were assessed using a two-sided t-test. The degree of freedom was always 65 (*i.e.,* 67 individuals – 2 fixed factors) and the coefficient of regression (Beta), the 95% boostraps confidence interval, the t-values, p-values and FDR-corrected p-values are reported for each fixed effect. For the sex effect, Female is the reference level. The coefficient (and its t-value) represents the extent to which the mean outcome for Males differs from that for Females; thus, a positive value indicates higher values in Males *compared to* Females, and *vice versa*. CI: confidence interval; The last column represents the significance: ns: not significant, . : significant before FDR but not after, *: $p_{FDR} < 0.05$, **: $p_{FDR} < 0.01$; ***: $p_{FDR} < 0.001$.

Networks	Intra FC (Mean ± SD)	Inter (Mean ± SD)	t-value	p-value	Significance
**Visual**	0.20 ± 0.074	0.0086 ± 0.015	13.20	3.44^{e-20}	***
**SomMot**	0.16 ± 0.051	0.022 ± 0.017	23.09	2.53^{e-33}	***
**DorsAttn**	0.092 ± 0.034	0.020 ± 0.019	18.60	5.93^{e-28}	***
**VentAttn**	0.13 ± 0.044	0.031 ± 0.015	20.78	1.14^{e-30}	***
**Limbic**	0.13 ± 0.044	0.025 ± 0.016	21.31	2.69^{e-31}	***
**Control**	0.067 ± 0.028	0.016 ± 0.015	14.95	2.76^{e-25}	***
**Default**	0.095 ± 0.040	0.013 ± 0.015	16.62	4.86^{e-20}	***
**Subcortical**	0.27 ± 0.081	0.029 ± 0.015	24.67	4.93^{e-33}	***
**Cerebellum**	0.16 ± 0.069	0.030 ± 0.018	18.30	1.47^{e-27}	***
**Spinal cord**	0.17 ± 0.077	0.033 ± 0.024	17.25	3.78^{e-26}	***

Table S8 | Intra *versus* inter-networks functional connectivity. The comparison between intra *versus* inter-network functional connectivity was obtained using a two-sided paired t-test. The degree of freedom was always 66, and the mean ± standard deviation, t-values and p-values are reported. The last column represents the significance: ns: not significant, *: p< 0.05, **: p< 0.01; ***: p < 0.001. SD: standard deviation

Networks	Intra FC			Inter FC		
	t-value	p-value	Signif.	t-value	p-value	Signif.
**Visual**	13.10	4.86^{e-20}	***	4.85	7.86^{e-07}	***
**SomMot**	25.11	1.74^{e-35}	***	10.56	8.01^{e-16}	***
**DorsAttn**	20.43	3.03^{e-30}	***	9.015	4.08^{e-13}	***
**VentAttn**	24.50	7.61^{e-35}	***	16.53	3.70^{e-25}	***
**Limbic**	23.33	1.38^{e-33}	***	12.27	1.09^{e-18}	***
**Control**	19.40	5.77^{e-29}	***	8.33	6.88^{e-12}	***
**Default**	19.70	2.38^{e-29}	***	7.00	1.57^{e-09}	***
**Subcortical**	26.90	2.74^{e-37}	***	15.37	1.74^{e-23}	***
**Cerebellum**	19.04	1.61^{e-28}	***	13.54	1.02^{e-20}	***
**Spinal cord**	18.37	1.18^{e-27}	***	11.40	3.11^{e-17}	***

Table S9 | Intra and inter-networks functional connectivity. The comparison between functional connectivity and 0 was obtained using a two-sided t-test against 0. The degree of freedom was always 66, and the t-values and p-values are reported. The last column represents the significance: ns: not significant, *: p< 0.05, **: p< 0.01; ***: p < 0.001. SD: standard deviation

Networks	Predictor	Beta	95% Bootstrap CI	t-value	p-value	p-value FDR	Sign.
**Visual**	age	2.98e-4	[1.24e-4 4.84e-4]	3.19	0.0022	0.0055	**
	sex	9.53e-4	[-0.015 -0.0036]	-2.98	0.0040	0.016	*
**SomMot**	age	2.84e-4	[7.76e-5 5.13e-4]	2.50	0.015	0.016	*
	sex	-0.0094	[-0.017 -0.0017]	-2.43	0.018	0.035	*
**DorsAttn**	age	4.40e-4	[1.92e-4 6.87e-4]	3.75	3.78^{e-04}	0.0012	**
	sex	-0.012	[-0.019 -0.0037]	-2.91	0.0050	0.016	*
**VentAttn**	age	2.72e-4	[6.64e-5 4.74e-4]	2.67	0.0096	0.012	*
	sex	-0.0093	[-0.016 -0.0026]	2.67	0.0096	0.024	*
**Limbic**	age	2.30e-4	[4.34e-5 4.44e-4]	2.050	0.044	0.044	*
	sex	0.0087	[-0.016 -0.0010]	-2.26	0.027	0.037	*
**Control**	age	4.60e-4	[2.55e-4 6.89e-4]	4.87	7.68^{e-05}	$7.68e^{-05}$	***
	sex	-0.0073	[-0.013 -0.0012]	-2.26	0.027	0.039	*
**Default**	age	2.82e-4	[9.01e-5 5.00e-4]	2.82	0.0064	0.0091	**
	sex	-0.011	[-0.017 -0.0039]	-3.11	0.0027	0.016	*
**Subcortical**	age	2.967e-4	[9.09e-5 5.12e-4]	2.86	0.0057	0.0091	**
	sex	-0.0052	[-0.012 0.0020]	-1.48	0.14	0.16	ns
**Cerebellum**	age	4.52e-4	[2.20e-4 6.70e-4]	3.84	2.28^{e-04}	0.0012	**
	sex	-0.0057	[-0.013 0.0020]	-1.41	0.16	0.16	ns
**Spinal cord**	age	4.69e-4	[1.25 0.00078]	2.93	0.0046	0.0091	**
	sex	-0.010	[-0.021 0.0014]	-1.89	0.062	0.078	ns

Table S10 | Age and sex effects on inter-network functional connectivity. The age-related effect on the inter-network was obtained for each brain network and the spinal cord using an ordinary least squares (OLS) model including age and sex as fixed effects. The fixed effects were assessed using a two-sided t-test. The degree of freedom was always 65 (*i.e.,* 67 individuals – 2 fixed factors) and the coefficient of regression (Beta), the 95% boostraps confidence interval, t-values, p-values and FDR-corrected p-values are reported for each fixed effect. For the sex effect, Female is the reference level. The coefficient (and its t-value) represents the extent to which the mean outcome for Males differs from that for Females; thus, a positive value indicates higher values in Males *compared to* Females, and *vice versa*. The last column represents the significance: ns: not significant, . : significant before FDR but not after, *: $p_{FDR} < 0.05$, **: $p_{FDR} < 0.01$; ***: $p_{FDR} < 0.001$. CI: confidence interval.

Networks	Predictor	Beta	95% Bootstrap CI	t-value	p-value	p-value FDR	Sign.
**Visual**	age	0.0020	[0.0010 0.0030]	4.36	4.85^{e-05}	1.62^{e-04}	***
	sex	-0.036	[-0.064 -0.0020]	-2.24	0.029	0.10	ns
**SomMot**	age	6.92	[-6.85e-4 8.92e-4]	0.18	0.85	0.85	ns
	sex	0.0052	[-0.022 0.028]	0.41	0.68	0.79	ns
**DorsAttn**	age	6.91e-4	[1.14e-4 0.0013]	2.80	0.0067	0.016	*
	sex	-0.017	[-0.033 -0.0025]	-2.08	0.041	0.10	.
**VentAttn**	age	6.21e-4	[-4.34e-5 1.36e-3]	1.97	0.052	0.10	ns
	sex	0.00043	[-0.020 0.022]	0.040	0.97	0.97	ns
**Limbic**	age	5.20e-4	[-8.23e-5 1.13e-3]	1.63	0.11	0.13	ns
	sex	-0.0060	[-0.028 0.016]	-0.55	0.59	0.79	ns
**Control**	age	3.58e-4	[-9.34e-5 8.04e-4]	1.79	0.077	0.13	ns
	sex	0.0025	[-0.010 0.016]	0.36	0.71	0.79	ns
**Default**	age	0.0011	[5.89e-4 0.0016]	4.51	2.80^{e-05}	$1.40e^{-04}$	***
	sex	0.0065	[-0.011 0.022]	0.76	0.45	0.75	ns
**Subcortical**	age	9.57e-4	[-1.20e-4 0.0021]	1.72	0.090	0.13	ns
	sex	0.041	[0.0036 0.078]	2.16	0.035	0.10	.
**Cerebellum**	age	0.0021	[0.0014 0.0029]	5.11	4.16e-06	3.16^{e-05}	***
	sex	-0.023	[-0.050 0.0025]	-1.60	0.11	0.22	ns
**Spinal cord**	age	4.47e-4	[-4.93e-4 0.0014]	0.85	0.39	0.44	ns
	sex	-0.055	[-0.087 -0.023]	-3.07	0.0030	0.030	*

Table S11 | Age and sex effects on intra-network functional connectivity. The age-related effect on intra-network was obtained for each brain network and the spinal cord using an ordinary least squares model including age and sex as fixed effects. The fixed effects were assessed using a two-sided t-test. The degree of freedom was always 65 (*i.e.,* 67 individuals – 2 fixed factors) and the coefficient of regression (Beta), the 95% boostraps confidence interval, the t-values, p-values and FDR-corrected p-values are reported for each fixed effect. For the sex effect, Female is the reference level. The coefficient (and its t-value) represents the extent to which the mean outcome for Males differs from that for Females; thus, a positive value indicates higher values in Males *compared to* Females, and *vice versa*. The last column represents the significance: ns: not significant, . : significant before FDR but not after, *: $p_{FDR} < 0.05$, **: $p_{FDR} < 0.01$; ***: $p_{FDR} < 0.001$. CI: confidence interval.

Features	ROI	Predictor	Beta	95% Bootstrap CI	t-value	p-value	p-value FDR	Sign.
ALFF	Cereb	age	0.23	[0.040 0.41]	1.73	0.083	0.131	ns
		sex	7.31	[0.88 13.76]	1.64	0.10	0.370	ns
	Control	age	0.15	[0.057 0.25]	2.49	0.013	0.047	*
		sex	1.85	[1.06 4.81]	0.92	0.36	0.482	ns
	Default	age	0.18	[0.089 0.27]	2.90	0.004	0.024	*
		sex	1.79	[-1.61 4.99]	0.85	0.40	0.482	ns
	Dors Attn	age	0.05	[-0.034 0.15]	0.91	0.36	0.442	ns
		sex	1.13	[-2.01 3.79]	0.57	0.57	0.571	ns
	Limbic	age	0.19	[0.060 0.33]	2.27	0.023	0.060	.
		sex	3.11	[-1.17 7.45]	1.06	0.29	0.453	ns
	SomMot	age	0.13	[0.054 0.23]	2.21	0.027	0.060	.
		sex	2.22	[-0.80 5.45]	1.06	0.29	0.453	ns
	SubCx	age	0.04	[-0.053 0.14]	0.67	0.50	0.505	ns
		sex	3.02	[-0.60 6.55]	1.35	0.17	0.387	ns
	Vent Attn	age	0.12	[0.027 0.22]	1.89	0.059	0.108	ns
		sex	3.22	[-0.035 6.20]	1.50	0.13	0.370	ns
	Visuel	age	0.10	[-0.015 0.24]	1.23	0.22	0.303	ns
		sex	1.95	[-2.48 6.33]	0.67	0.50	0.555	ns
	Spinal Dorsal	age	0.06	[0.029 0.095]	2.85	0.004	0.024	*
		sex	3.27	[2.15 4.42]	4.33	1.48^{e-05}	0.0016	**
	Spinal Ventral	age	0.02	[-0.016 0.051]	0.83	0.407	0.447	ns
		sex	2.22	[1.18 3.31]	3.01	0.003	0.014	*
Embed Dist	Cereb	age	-8.30e-5	[-1.59e-4 1.53e-4]	-0.08	0.936	0.936	ns
		sex	-1.07e-4	[-5.55e-4 0.0054]	-0.03	0.976	0.976	ns
	Control	age	-2.42e-4	[-3.90e-04 -5.72e-5]	-2.40	0.016	0.090	ns
		sex	0.0094	[0.0043 0.015]	2.74	0.006	0.033	*
	Default	age	1.51e-4	[-2.87e-04 1.80e-5]	-1.61	0.106	0.195	ns
		sex	0.0097	[0.0047 0.014]	3.03	0.002	0.027	*
	Dors Attn	age	-2.39e-4	[-4.37e-04 -1.47e-5]	-1.99	0.047	0.129	.
		sex	0.010	[0.0041 0.017]	2.54	0.011	0.041	*
	Limbic	age	-1.23e-4	[-3.03e-04 4.28e-5]	-1.20	0.231	0.317	ns
		sex	0.0078	[0.0024 0.013]	2.20	0.028	0.076	.
	SomMot	age	-3.15e-4	[-4.74 e-4 -1.23 e-4]	-3.08	0.002	0.023	*
		sex	6.39e-4	[7.78e-4 0.012]	1.83	0.068	0.124	ns
	SubCx	age	-1.10e-4	[-2.70e-04 6.19e-5]	-0.92	0.358	0.413	ns
		sex	0.0065	[4.34e-4 0.013]	1.58	0.113	0.156	ns
	Vent Attn	age	-1.49e-4	[-3.25e-04 4.43e-5]	-1.42	0.156	0.245	ns
		sex	0.0071	[0.0018 0.012]	1.97	0.049	0.108	ns
	Visuel	age	-1.07e-4	[-2.9 e-4 1.05 e-4]	-0.89	0.376	0.413	ns
		sex	0.0057	[-2.70e-4 0.012]	1.37	0.170	0.207	ns
	Spinal Dorsal	age	1.24e-4	[2.73e-5 2.36e-4]	2.09	0.037	0.129	.
		sex	-2.60e-4	[-0.0035 0.0030]	-0.13	0.898	0.976	ns
	Spinal Ventral	age	1.19e-4	[2.04e-5 2.21e-4]	1.88	0.061	0.134	ns
		sex	-0.0038	[-0.0069 -4.28e-4]	-1.75	0.079	0.125	ns
Entrop. Pairs	Cereb	age	5.7e-5	[-9.54e-05 1.92e-04]	0.56	0.574	0.647	ns
		sex	-7.71e-4	[-0.0058 0.0044]	-0.22	0.825	0.825	ns
	Control	age	3.70e-4	[1.41e-4 5.50 e-4]	3.24	0.001	0.012	*
		sex	-0.0116	[-0.018 -0.0053]	-2.92	0.004	0.029	*
	Default	age	1.43e-4	[-3.61e-05 2.94e-04]	1.39	0.164	0.258	ns
		sex	-0.0087	[-0.014 -0.0035]	-2.52	0.012	0.037	*
	Dors Attn	age	3.63e-4	[1.28e-4 5.79]	2.74	0.006	0.023	*
		sex	-0.013	[-0.020 -0.0054]	-2.79	0.005	0.029	ns
	Limbic	age	-4.9e-5	[-2.51 1.46]	-0.46	0.647	0.647	ns
		sex	-0.0089	[-0.014 -0.0031]	-2.41	0.016	0.037	*
	SomMot	age	3.71e-4	[1.16e-4 5.56e-4]	3.06	0.002	0.012	*

		sex	-0.0072	[-0.014 -2.87e-4]	-1.73	0.084	0.115	ns
	SubCx	age	1.36e-4	[-6.95e-5 3.22e-4]	1.02	0.310	0.426	ns
		sex	-0.011	[-0.018 -0.0034]	-2.32	0.020	0.037	*
	Vent Attn	age	1.90e-4	[-3.39e-5 3.66e-4]	1.68	0.093	0.170	ns
		sex	-0.0091	[-0.015 -0.0034]	-2.37	0.018	0.037	*
	Visuel	age	2.54e-4	[7.16e-6 4.74e-4]	1.97	0.048	0.111	.
		sex	-0.0092	[-0.016 -0.0021]	-2.09	0.036	0.057	ns
	Spinal Dorsal	age	-1.21e-4	[-2.37e-4 -1.70e-5]	-1.96	0.050	0.111	ns
		sex	-6.03e-4	[-0.0044 0.0030]	-0.29	0.776	0.825	ns
	Spinal Ventral	age	-3.4 e-5	[-1.36e-4 6.04e-5]	-0.50	0.615	0.647	ns
		sex	0.0024	[-7.20e-4 0.0056]	1.04	0.298	0.365	ns
Forecast Error	Cereb	age	-1.94e-4	[-4.82e-4 6.42e-5]	-1.01	0.312	0.490	ns
		sex	8.45e-5	[-0.0098 0.010]	0.01	0.990	0.990	ns
	Control	age	2.55e-4	[-4.18e-5 5.32e-4]	1.35	0.178	0.489	ns
		sex	-0.019	[-0.029 -0.0093]	-2.95	0.003	0.017	*
	Default	age	-1.04e-4	[-4.18e-4 1.71e-4]	-0.54	0.588	0.809	ns
		sex	-0.014	[-0.024 -0.0041]	-2.16	0.030	0.078	.
	Dors Attn	age	2.45e-4	[-1.48e-4 5.76e-4]	1.17	0.241	0.490	ns
		sex	-0.021	[-0.032 -0.011]	-2.99	0.003	0.017	*
	Limbic	age	-2.07e-4	[-5.57e-4 8.16e-5]	-1.06	0.289	0.490	ns
		sex	-0.013	[-0.023 -0.0030]	-1.92	0.055	0.101	ns
	SomMot	age	3.25e-4	[-3.70e-5 6.81e-4]	1.49	0.135	0.489	ns
		sex	-0.016	[-0.029 -0.0047]	-2.10	0.035	0.078	.
	SubCx	age	-8.82e-4	[-4.47e-4 2.57 e-4]	-0.38	0.704	0.860	ns
		sex	-0.013	[-0.026 -4.94e-4]	-1.67	0.095	0.137	ns
	Vent Attn	age	8.3e-4	[-3.34e-4 3.44e-4]	0.04	0.965	0.965	ns
		sex	-0.017	[-0.027 -0.0068]	-2.59	0.010	0.035	*
	Visuel	age	1.87e-4	[-3.71e-4 3.57e-4]	0.09	0.930	0.965	ns
		sex	-0.012	[-0.022 -0.0011]	-1.65	0.100	0.137	ns
	Spinal Dorsal	age	-4.52e-4	[-7.18e-4 -2.22 e-4]	-3.19	0.001	0.016	*
		sex	-2.42e-4	[-0.0076 0.0072]	-0.05	0.960	0.990	ns
	Spinal Ventral	age	-3.05e-4	[-4.90e-4 -1.21e-4]	-2.50	0.012	0.068	.
		sex	0.0023	[-0.0037 0.00861]	0.57	0.572	0.699	ns
Low Freq Power	Cereb	age	-2.43e-4	[-7.58e-4 2.47e-4]	-0.69	0.489	0.751	ns
		sex	-0.0020	[-0.020 0.016]	-0.17	0.868	0.868	ns
	Control	age	6.23e-4	[1.35e-6 1.16e-3]	1.92	0.055	0.152	ns
		sex	-0.030	[-0.047 -0.012]	-2.64	0.008	0.025	*
	Default	age	-2.30e-5	[-4.98e-4 4.02e-4]	-0.07	0.943	0.943	ns
		sex	-2.85	[-0.044 -0.012]	-2.61	0.009	0.025	*
	Dors Attn	age	6.29e-4	[-1.00e-4 0.0012]	1.82	0.069	0.152	ns
		sex	-0.038	[-0.055 -0.021]	-3.20	0.001	0.010	*
	Limbic	age	-2.97e-4	[-0.0010 3.71e-4]	-0.78	0.438	0.751	ns
		sex	-0.025e-4	[-0.047 -0.0032]	-1.88	0.060	0.087	ns
	SomMot	age	6.94 e-4	[-6.16e-5 1.28e-3]	1.86	0.062	0.152	ns
		sex	-0.028	[-0.047 -0.0091]	-2.17	0.030	0.062	.
	SubCx	age	2.20e-4	[-5.18e-4 8.65e-4]	0.50	0.614	0.751	ns
		sex	-0.032	[-0.055 -0.0098]	-2.12	0.034	0.062	.
	Vent Attn	age	1.82e-4	[-4.70e-4 7.08e-4]	0.55	0.582	0.751	ns
		sex	-0.035	[-0.053 -0.019]	-3.13	0.002	0.010	*
	Visuel	age	1.23 e-4	[-4.31e-4 6.02e-4]	0.39	0.696	0.765	ns
		sex	-0.020	[-0.036 -0.0031]	-1.85	0.064	0.087	ns
	Spinal Dorsal	age	-7.50e-4	[-0.0012 -2.27e-4]	-2.89	0.004	0.042	*
		sex	-0.0067	[-0.020 0.0072]	-0.76	0.447	0.546	ns
	Spinal Ventral	age	-4.53e-4	[-8.15e-4 -3.54e-5]	-1.83	0.067	0.152	ns
		sex	0.0015	[-0.012 0.015]	0.18	0.856	0.868	ns

	Cereb	age	0.79	[-0.090 1.62]	1.33	0.185	0.290	ns
		sex	57.78	[25.03 90.34]	2.83	0.005	0.052	.
	Control	age	1.14	[0.37 1.88]	2.44	0.015	0.051	.
		sex	22.26	[0.13 47.01]	1.40	0.163	0.299	ns
	Default	age	1.06	[0.31 1.81]	2.35	0.019	0.051	.
		sex	22.57	[-0.62 46.24]	1.46	0.143	0.299	ns
	Dors	age	1.17	[0.29 1.97]	2.24	0.025	0.055	.
	Attn	sex	20.54	[-8.45 47.07]	1.15	0.249	0.312	ns
	Limbic	age	0.68	[-0.38 1.69]	0.90	0.366	0.478	ns
		sex	29.32	[-6.54 69.04]	1.14	0.256	0.312	ns
mean	SomMot	age	1.53	[0.73 2.38]	2.85	0.004	0.048	*
		sex	26.30	[-2.26 52.76]	1.43	0.151	0.299	ns
	SubCx	age	-0.25	[-1.29 0.74]	-0.40	0.691	0.691	ns
		sex	14.06	[-18.92 45.77]	0.66	0.509	0.545	ns
	Vent	age	1.29	[0.50 2.06]	2.52	0.012	0.051	.
	Attn	sex	30.08	[3.74 56.41]	1.72	0.085	0.299	ns
	Visuel	age	-0.84	[-1.76 0.12]	-1.42	0.157	0.287	ns
		sex	12.34	[-17.87 44.33]	0.60	0.545	0.545	ns
	Spinal	age	0.27	[-0.71 1.17]	0.42	0.675	0.691	ns
	Dorsal	sex	40.76	[10.77 74.88]	1.84	0.066	0.299	ns
	Spinal	age	-0.54	[-1.57 0.43]	-0.86	0.391	0.478	ns
	Ventral	sex	27.94	[-3.02 60.42]	1.29	0.197	0.310	ns
	Cereb	age	0.0067	[0.0039 0.0098]	3.64	2.75^{e-04}	0.0010	**
		sex	0.0861	[-0.0095 0.179]	1.37	0.171	0.316	ns
	Control	age	0.0017	[2.70e-4 0.0035]	1.91	0.056	0.083	ns
		sex	0.0421	[-0.0064 0.087]	1.36	0.172	0.316	ns
	Default	age	0.0030	[0.0016 0.0047]	3.35	8.11^{e-04}	0.0022	**
		sex	0.0344	[-0.011 0.086]	1.11	0.267	0.419	ns
	Dors	age	-0.0008	[-0.0022 7.40e-4]	-0.93	0.355	0.355	ns
	Attn	sex	0.0228	[-0.019 0.068]	0.74	0.460	0.562	ns
	Limbic	age	0.0029	[0.0015 0.0045]	3.27	0.001	0.0024	**
		sex	0.0262	[-0.022 0.072]	0.86	0.389	0.535	ns
SD	SomMot	age	0.0016	[3.09e-4 0.0029]	1.88	0.060	0.083	ns
		sex	0.0403	[-0.007 0.085]	1.38	0.169	0.316	ns
	SubCx	age	0.0033	[0.0020 0.0049]	3.84	1.24^{e-04}	0.0010	**
		sex	0.0731	[0.026 0.11]	2.48	0.013	0.122	.
	Vent	age	0.0025	[0.0013 0.0040]	3.14	0.002	0.043	*
	Attn	sex	0.0622	[0.022 0.10]	2.29	0.022	0.122	.
	Visuel	age	0.0026	[4.08e-4 0.0051]	1.75	0.081	0.099	ns
		sex	0.0117	[-0.071 0.083]	0.23	0.817	0.861	ns
	Spinal	age	0.0143	[0.0084 0.020]	3.74	1.18^{e-04}	0.0010	**
	Dorsal	sex	0.2227	[0.021 0.42]	1.71	0.088	0.316	ns
	Spinal	age	0.0059	[2.78e-4 0.011]	1.58	0.114	0.126	ns
	Ventral	sex	0.0221	[-0.17 0.22]	0.17	0.861	0.861	ns

Table S12 | Age and sex effects on functional dynamic profiles in both brain and spinal cord regions. The age-related effect was obtained for each feature and four regions using a linear mixed model including age and sex as fixed effects and participant as random effects. The fixed effects were assessed using a two-sided t-test. We report the fixed effects of the model when the age effect was significant after FDR correction for at least one region. The degree of freedom was always 65 (*i.e.,* 67 individuals – 2 fixed factors) and the coefficient of regression (Beta), the 95% boostraps confidence interval, the t-values, p-values and FDR-corrected p-values are reported for each fixed effect. For the sex effect, Female is the reference level. The coefficient (and its t-value) represents the extent to which the mean outcome for Males differs from that for Females; thus, a positive value indicates higher values in Males *compared to*

Females, and *vice versa*. The last column represents the significance: ns: not significant. : significant before FDR but not after, *: $p_{FDR} < 0.05$, **: $p_{FDR} < 0.01$; ***: $p_{FDR} < 0.001$. CI: confidence interval. DL: dorsal left, DR: dorsal right, VL: ventral left, VR: ventral right, SD: Standard deviation, ALFF: amplitude of low frequency fluctuation, ACF: autocorrelation function.
