# Supplementary material for: Spinal cord structural and functional architecture and its shared organization with the brain across the adult lifespan
Source: Nat Commun. 2026 Apr 16;17:5269. doi: 10.1038/s41467-026-71963-2 (PMC13265777; doi:10.1038/s41467-026-71963-2)
Supplement: Supplementary file 2 — Reporting Summary [file 41467_2026_71963_MOESM2_ESM.pdf]

Corresponding author(s): Caroline Landelle

Last updated by author(s): Mar 20, 2026

Reporting Summary

Nature Portfolio wishes to improve the reproducibility of the work that we publish. This form provides structure for consistency and transparency in reporting. For further information on Nature Portfolio policies, see our Editorial Policies and the Editorial Policy Checklist.

Statistics

For all statistical analyses, confirm that the following items are present in the figure legend, table legend, main text, or Methods section.

n/a	Confirmed	
☐	☒	The exact sample size (*n*) for each experimental group/condition, given as a discrete number and unit of measurement
☐	☒	A statement on whether measurements were taken from distinct samples or whether the same sample was measured repeatedly
☐	☒	The statistical test(s) used AND whether they are one- or two-sided<br>*Only common tests should be described solely by name; describe more complex techniques in the Methods section.*
☐	☒	A description of all covariates tested
☐	☒	A description of any assumptions or corrections, such as tests of normality and adjustment for multiple comparisons
☐	☒	A full description of the statistical parameters including central tendency (e.g. means) or other basic estimates (e.g. regression coefficient) AND variation (e.g. standard deviation) or associated estimates of uncertainty (e.g. confidence intervals)
☐	☒	For null hypothesis testing, the test statistic (e.g. *F*, *t*, *r*) with confidence intervals, effect sizes, degrees of freedom and *P* value noted<br>*Give P values as exact values whenever suitable.*
☒	☐	For Bayesian analysis, information on the choice of priors and Markov chain Monte Carlo settings
☒	☐	For hierarchical and complex designs, identification of the appropriate level for tests and full reporting of outcomes
☐	☒	Estimates of effect sizes (e.g. Cohen's *d*, Pearson's *r*), indicating how they were calculated

Our web collection on statistics for biologists contains articles on many of the points above.

Software and code

Policy information about availability of computer code

Data collection	No specific code were used for data collection
Data analysis	The pipelines for analyses are available here: https://github.com/CarolineLndl/Landelle_spinebrain_aging

For manuscripts utilizing custom algorithms or software that are central to the research but not yet described in published literature, software must be made available to editors and reviewers. We strongly encourage code deposition in a community repository (e.g. GitHub). See the Nature Portfolio guidelines for submitting code & software for further information.

Data

Policy information about availability of data

All manuscripts must include a data availability statement. This statement should provide the following information, where applicable:

- Accession codes, unique identifiers, or web links for publicly available datasets
- A description of any restrictions on data availability
- For clinical datasets or third party data, please ensure that the statement adheres to our policy

The datasets generated and analyzed during the current study are available upon request.
Data from one representative participant are available at: https://doi.org/10.18112/openneuro.ds007313.v1.0.0

Research involving human participants, their data, or biological material

Policy information about studies with human participants or human data. See also policy information about sex, gender (identity/presentation), and sexual orientation and race, ethnicity and racism.

Reporting on sex and gender	Sex was determined based on participants' self-reports. The cohort comprised individuals aged 20 to 80 years, and participants were matched for sex across the different age ranges to ensure balanced representation (Fig S2A). Sex was included as a covariate in all statistical analyses, and the effects of sex are reported in the main text or in the supplementary materials.<br><br>Gender identity was not assessed in the present study. According to Statistics Canada (2021), approximately 0.34% of Canadians identify as transgender or non-binary. Extrapolating this proportion to our sample of 70 participants suggests that the number of participants identifying as transgender or non-binary would likely be very small. Given this expected sample composition, gender was not included as a factor in the analyses.
Reporting on race, ethnicity, or other socially relevant groupings	Race, ethnicity or other socially groupings were not included in our study
Population characteristics	The cohort comprised right-handed, healthy individuals aged 20 to 80 years, with participants matched for sex across age ranges to ensure balanced representation (Fig. S2A). The sample included 36 females (mean age = 48.82 ± 16.94 years).
Recruitment	Participants were recruited through advertisements posted across McGill University and on social media platforms.
Ethics oversight	The experiment was approved by the local ethics committee, McGill University Health Centre (MUHC REB 2019-4626)

Note that full information on the approval of the study protocol must also be provided in the manuscript.

Field-specific reporting

Please select the one below that is the best fit for your research. If you are not sure, read the appropriate sections before making your selection.

☒ Life sciences ☐ Behavioural & social sciences ☐ Ecological, evolutionary & environmental sciences

For a reference copy of the document with all sections, see nature.com/documents/nr-reporting-summary-flat.pdf

Life sciences study design

All studies must disclose on these points even when the disclosure is negative.

Sample size	A sample size of 70 participants was considered sufficient for this study for several reasons. First, spinal cord imaging datasets are rare because acquiring high-quality data in this region is technically challenging, and no large cohort exists for healthy aging, making formal sample size calculations difficult. Second, our previous spinal fMRI study in a smaller cohort of about 20 healthy control participants—albeit not multimodal—demonstrated replicable findings across dataset (Kinany, Landelle et al. 2024). Third, in a spinal fMRI study of 54 Parkinson's disease patients, robust results were observed (Landelle et al. 2023), supporting the adequacy of a sample of this size for detecting meaningful effects in multimodal imaging analyses .
Data exclusions	Quality control involved visual inspection of quantitative imaging data, as well as assessment of framewise displacement and temporal signal-to-noise ratio (tSNR) for functional MRI data (Fig. S2). Following quality control, the final sample comprised 67 participants with T1-weighted, T2-weighted, and functional MRI data, of whom 55 also had magnetization transfer (MT) and/or diffusion-weighted imaging (DWI) data. A total of 54 participants had all imaging sequences (T1-weighted, T2-weighted, functional MRI, MT, and DWI) and were included in analyses requiring the full set of modalities, whereas analyses specific to individual imaging types included the corresponding 67 or 55 participants as appropriate.
Replication	As this dataset is unique and not available elsewhere, direct replication of our analyses in an independent cohort is not feasible. Nevertheless, the multimodal acquisition allowed us to replicate findings across different imaging modalities—for example, functional observations could be confirmed by structural data and vice versa (i.e., age-related atrophy of the dorsal spinal cord). By integrating analyses of both the spinal cord and the brain, we were able to reproduce key findings at the brain level that were initially observed in the spinal cord, providing converging evidence that the aging process affects the central nervous system as a whole (decrease GM, dedifferenciation processes ...).
Randomization	All statistical analyses were complemented by bootstrap resampling. Permutation testing was additionally performed for models predicting participants' age.
Blinding	Blinding was not applicable to this study, as all participants were included in a single observational cohort and no experimental interventions or group assignments were performed.

Behavioural & social sciences study design

All studies must disclose on these points even when the disclosure is negative.

Study description	*Briefly describe the study type including whether data are quantitative, qualitative, or mixed-methods (e.g. qualitative cross-sectional, quantitative experimental, mixed-methods case study).*
Research sample	*State the research sample (e.g. Harvard university undergraduates, villagers in rural India) and provide relevant demographic information (e.g. age, sex) and indicate whether the sample is representative. Provide a rationale for the study sample chosen. For studies involving existing datasets, please describe the dataset and source.*
Sampling strategy	*Describe the sampling procedure (e.g. random, snowball, stratified, convenience). Describe the statistical methods that were used to predetermine sample size OR if no sample-size calculation was performed, describe how sample sizes were chosen and provide a rationale for why these sample sizes are sufficient. For qualitative data, please indicate whether data saturation was considered, and what criteria were used to decide that no further sampling was needed.*
Data collection	*Provide details about the data collection procedure, including the instruments or devices used to record the data (e.g. pen and paper, computer, eye tracker, video or audio equipment) whether anyone was present besides the participant(s) and the researcher, and whether the researcher was blind to experimental condition and/or the study hypothesis during data collection.*
Timing	*Indicate the start and stop dates of data collection. If there is a gap between collection periods, state the dates for each sample cohort.*
Data exclusions	*If no data were excluded from the analyses, state so OR if data were excluded, provide the exact number of exclusions and the rationale behind them, indicating whether exclusion criteria were pre-established.*
Non-participation	*State how many participants dropped out/declined participation and the reason(s) given OR provide response rate OR state that no participants dropped out/declined participation.*
Randomization	*If participants were not allocated into experimental groups, state so OR describe how participants were allocated to groups, and if allocation was not random, describe how covariates were controlled.*

Ecological, evolutionary & environmental sciences study design

All studies must disclose on these points even when the disclosure is negative.

Study description	*Briefly describe the study. For quantitative data include treatment factors and interactions, design structure (e.g. factorial, nested, hierarchical), nature and number of experimental units and replicates.*
Research sample	*Describe the research sample (e.g. a group of tagged Passer domesticus, all Stenocereus thurberi within Organ Pipe Cactus National Monument), and provide a rationale for the sample choice. When relevant, describe the organism taxa, source, sex, age range and any manipulations. State what population the sample is meant to represent when applicable. For studies involving existing datasets, describe the data and its source.*
Sampling strategy	*Note the sampling procedure. Describe the statistical methods that were used to predetermine sample size OR if no sample-size calculation was performed, describe how sample sizes were chosen and provide a rationale for why these sample sizes are sufficient.*
Data collection	*Describe the data collection procedure, including who recorded the data and how.*
Timing and spatial scale	*Indicate the start and stop dates of data collection, noting the frequency and periodicity of sampling and providing a rationale for these choices. If there is a gap between collection periods, state the dates for each sample cohort. Specify the spatial scale from which the data are taken*
Data exclusions	*If no data were excluded from the analyses, state so OR if data were excluded, describe the exclusions and the rationale behind them, indicating whether exclusion criteria were pre-established.*
Reproducibility	*Describe the measures taken to verify the reproducibility of experimental findings. For each experiment, note whether any attempts to repeat the experiment failed OR state that all attempts to repeat the experiment were successful.*
Randomization	*Describe how samples/organisms/participants were allocated into groups. If allocation was not random, describe how covariates were controlled. If this is not relevant to your study, explain why.*
Blinding	*Describe the extent of blinding used during data acquisition and analysis. If blinding was not possible, describe why OR explain why blinding was not relevant to your study.*

Did the study involve field work? ☐ Yes ☐ No

Field work, collection and transport

Field conditions	*Describe the study conditions for field work, providing relevant parameters (e.g. temperature, rainfall).*
Location	*State the location of the sampling or experiment, providing relevant parameters (e.g. latitude and longitude, elevation, water depth).*
Access & import/export	*Describe the efforts you have made to access habitats and to collect and import/export your samples in a responsible manner and in compliance with local, national and international laws, noting any permits that were obtained (give the name of the issuing authority, the date of issue, and any identifying information).*
Disturbance	*Describe any disturbance caused by the study and how it was minimized.*

Reporting for specific materials, systems and methods

We require information from authors about some types of materials, experimental systems and methods used in many studies. Here, indicate whether each material, system or method listed is relevant to your study. If you are not sure if a list item applies to your research, read the appropriate section before selecting a response.

Materials & experimental systems

n/a	Involved in the study
☒	☐ Antibodies
☒	☐ Eukaryotic cell lines
☒	☐ Palaeontology and archaeology
☒	☐ Animals and other organisms
☒	☐ Clinical data
☒	☐ Dual use research of concern
☒	☐ Plants

Methods

n/a	Involved in the study
☒	☐ ChIP-seq
☒	☐ Flow cytometry
☐	☒ MRI-based neuroimaging

Antibodies

Antibodies used	*Describe all antibodies used in the study; as applicable, provide supplier name, catalog number, clone name, and lot number.*
Validation	*Describe the validation of each primary antibody for the species and application, noting any validation statements on the manufacturer's website, relevant citations, antibody profiles in online databases, or data provided in the manuscript.*

Eukaryotic cell lines

Policy information about cell lines and Sex and Gender in Research

Cell line source(s)	*State the source of each cell line used and the sex of all primary cell lines and cells derived from human participants or vertebrate models.*
Authentication	*Describe the authentication procedures for each cell line used OR declare that none of the cell lines used were authenticated.*
Mycoplasma contamination	*Confirm that all cell lines tested negative for mycoplasma contamination OR describe the results of the testing for mycoplasma contamination OR declare that the cell lines were not tested for mycoplasma contamination.*
Commonly misidentified lines (See ICLAC register)	*Name any commonly misidentified cell lines used in the study and provide a rationale for their use.*

Palaeontology and Archaeology

Specimen provenance	*Provide provenance information for specimens and describe permits that were obtained for the work (including the name of the issuing authority, the date of issue, and any identifying information). Permits should encompass collection and, where applicable, export.*
Specimen deposition	*Indicate where the specimens have been deposited to permit free access by other researchers.*

Dating methods	*If new dates are provided, describe how they were obtained (e.g. collection, storage, sample pretreatment and measurement), where they were obtained (i.e. lab name), the calibration program and the protocol for quality assurance OR state that no new dates are provided.*

☐ Tick this box to confirm that the raw and calibrated dates are available in the paper or in Supplementary Information.

Ethics oversight	*Identify the organization(s) that approved or provided guidance on the study protocol, OR state that no ethical approval or guidance was required and explain why not.*

Note that full information on the approval of the study protocol must also be provided in the manuscript.

Animals and other research organisms

Policy information about studies involving animals; ARRIVE guidelines recommended for reporting animal research, and Sex and Gender in Research

Laboratory animals	*For laboratory animals, report species, strain and age OR state that the study did not involve laboratory animals.*
Wild animals	*Provide details on animals observed in or captured in the field; report species and age where possible. Describe how animals were caught and transported and what happened to captive animals after the study (if killed, explain why and describe method; if released, say where and when) OR state that the study did not involve wild animals.*
Reporting on sex	*Indicate if findings apply to only one sex; describe whether sex was considered in study design, methods used for assigning sex. Provide data disaggregated for sex where this information has been collected in the source data as appropriate; provide overall numbers in this Reporting Summary. Please state if this information has not been collected. Report sex-based analyses where performed, justify reasons for lack of sex-based analysis.*
Field-collected samples	*For laboratory work with field-collected samples, describe all relevant parameters such as housing, maintenance, temperature, photoperiod and end-of-experiment protocol OR state that the study did not involve samples collected from the field.*
Ethics oversight	*Identify the organization(s) that approved or provided guidance on the study protocol, OR state that no ethical approval or guidance was required and explain why not.*

Note that full information on the approval of the study protocol must also be provided in the manuscript.

Clinical data

Policy information about clinical studies

All manuscripts should comply with the ICMJE guidelines for publication of clinical research and a completed CONSORT checklist must be included with all submissions.

Clinical trial registration	*Provide the trial registration number from ClinicalTrials.gov or an equivalent agency.*
Study protocol	*Note where the full trial protocol can be accessed OR if not available, explain why.*
Data collection	*Describe the settings and locales of data collection, noting the time periods of recruitment and data collection.*
Outcomes	*Describe how you pre-defined primary and secondary outcome measures and how you assessed these measures.*

Dual use research of concern

Policy information about dual use research of concern

Hazards

Could the accidental, deliberate or reckless misuse of agents or technologies generated in the work, or the application of information presented in the manuscript, pose a threat to:

No	Yes	
☐	☐	Public health
☐	☐	National security
☐	☐	Crops and/or livestock
☐	☐	Ecosystems
☐	☐	Any other significant area

Experiments of concern

Does the work involve any of these experiments of concern:

No	Yes	
☐	☐	Demonstrate how to render a vaccine ineffective
☐	☐	Confer resistance to therapeutically useful antibiotics or antiviral agents
☐	☐	Enhance the virulence of a pathogen or render a nonpathogen virulent
☐	☐	Increase transmissibility of a pathogen
☐	☐	Alter the host range of a pathogen
☐	☐	Enable evasion of diagnostic/detection modalities
☐	☐	Enable the weaponization of a biological agent or toxin
☐	☐	Any other potentially harmful combination of experiments and agents

Plants

Seed stocks	N/A
Novel plant genotypes	N/A
Authentication	N/A

ChIP-seq

Data deposition

☐ Confirm that both raw and final processed data have been deposited in a public database such as GEO.

☐ Confirm that you have deposited or provided access to graph files (e.g. BED files) for the called peaks.

Data access links<br>*May remain private before publication.*	*For "Initial submission" or "Revised version" documents, provide reviewer access links. For your "Final submission" document, provide a link to the deposited data.*
Files in database submission	*Provide a list of all files available in the database submission.*
Genome browser session<br>(e.g. UCSC)	*Provide a link to an anonymized genome browser session for "Initial submission" and "Revised version" documents only, to enable peer review. Write "no longer applicable" for "Final submission" documents.*

Methodology

Replicates	*Describe the experimental replicates, specifying number, type and replicate agreement.*
Sequencing depth	*Describe the sequencing depth for each experiment, providing the total number of reads, uniquely mapped reads, length of reads and whether they were paired- or single-end.*
Antibodies	*Describe the antibodies used for the ChIP-seq experiments; as applicable, provide supplier name, catalog number, clone name, and lot number.*
Peak calling parameters	*Specify the command line program and parameters used for read mapping and peak calling, including the ChIP, control and index files used.*
Data quality	*Describe the methods used to ensure data quality in full detail, including how many peaks are at FDR 5% and above 5-fold enrichment.*
Software	*Describe the software used to collect and analyze the ChIP-seq data. For custom code that has been deposited into a community repository, provide accession details.*

Flow Cytometry

Plots

Confirm that:

- [ ] The axis labels state the marker and fluorochrome used (e.g. CD4-FITC).
- [ ] The axis scales are clearly visible. Include numbers along axes only for bottom left plot of group (a 'group' is an analysis of identical markers).
- [ ] All plots are contour plots with outliers or pseudocolor plots.
- [ ] A numerical value for number of cells or percentage (with statistics) is provided.

Methodology

Sample preparation	*Describe the sample preparation, detailing the biological source of the cells and any tissue processing steps used.*
Instrument	*Identify the instrument used for data collection, specifying make and model number.*
Software	*Describe the software used to collect and analyze the flow cytometry data. For custom code that has been deposited into a community repository, provide accession details.*
Cell population abundance	*Describe the abundance of the relevant cell populations within post-sort fractions, providing details on the purity of the samples and how it was determined.*
Gating strategy	*Describe the gating strategy used for all relevant experiments, specifying the preliminary FSC/SSC gates of the starting cell population, indicating where boundaries between "positive" and "negative" staining cell populations are defined.*

- [ ] Tick this box to confirm that a figure exemplifying the gating strategy is provided in the Supplementary Information.

Magnetic resonance imaging

Experimental design

Design type	Multimodal acquisition including Resting-state (i.e., no explicit task)
Design specifications	Each participant completed a single MRI session (1h) during which they underwent T1-weighted imaging (brain and spinal cord), functional MRI (brain and spinal cord), T2-weighted imaging (spinal cord), magnetization transfer imaging (spinal cord), and diffusion-weighted imaging (spinal cord).
Behavioral performance measures	No behavioral measures were assessed in this study.

Acquisition

Imaging type(s)	functional, structural (T1w) and microstructural (T2*w, Magnetization transfert and diffusion)
Field strength	3T (Brain and spinal cord)
Sequence & imaging parameters	T2*w: spinal cord, 30 slices; 0.4x0.4x5 mm3; Grappa; TR: 34ms; TE: 14ms; Flip angle:5 Accelation factor: 2<br>MT: spinal cord, 30 slices; 0.9x0.9x5 mm3; Grappa; TR: 35ms, TE: 2.62ms; Flip angle:9 Accelation factor: 2<br>DWI: spinal cord, 24 slices; 0.9x0.9x5 mm3; Grappa; TR: 780ms; TE: 64ms Flip angle:90<br>T1w (MPRAGE): brain and spinal cord, 288 slices; 1.3x1.3x1.3 mm3; Grappa; TR: 2300ms, TE: 3.3ms; Flip angle:9 Accelation factor: 2<br>EPI (echo planar): brain and spinal cord, 69 slices; 230 volumes; 1.6x1.6x4 mm3; Grappa; TR: 1.55ms, TE: 23ms; Flip angle:70; Accelation factor: 2; multiband 3
Area of acquisition	cervical Spinal cord (T2*w; MT; DWI)<br>brain + cervical spinal cord (T1w and Functional)

Diffusion MRI — [x] Used — [ ] Not used

Preprocessing

Preprocessing software	Preprocessing pipeline can be found here : https://github.com/CarolineLndl/Landelle_spinebrain_aging<br>Data were sorted, transformed into NIFTI, and organized using the Brain Imaging Data Structure (BIDS) standard (with dcm2bids v2.1.4). We used an in-house python pipeline79 (publicly available) based on the Spinal Cord Toolbox (SCT, version 5.6.0)80, the Oxford Center for fMRI of the Software Library (FSL, version 5.0), the Statistical Parametric Mapping (SPM12, running on Matlab 2021b), the Tapas PhysiO toolbox (release 2022a, V8.1.0)81and the Nilearn toolbox (version 0.9.1).
Normalization	Brain normalization was performed using a cohort-specific DARTEL template. Spinal cord normalization was conducted using the Spinal Cord Toolbox (SCT).

Normalization template | Brain normalization was done in MNI space; spinal cord normalization was computed in PAM50 space

Noise and artifact removal | Functional data were denoised using RETROICOR to account for physiological signals and CompCor to remove noise related to cerebrospinal fluid (CSF) signals.

Volume censoring | Framewise displacement was measured for each functional volume.

Statistical modeling & inference

Model type and settings | Several analytical approaches were employed, including morphometric similarity analysis, ordinary least squares modeling, age prediction, functional connectivity analysis, innovation-driven co-activation patterns (iCAPs) analysis, time-series dynamic property measurements, and dynamic profile similarity assessment.

Effect(s) tested | Similarity, functional connectivity, structure-function coupling and age-effect

Specify type of analysis: ☐ Whole brain ☒ ROI-based ☐ Both

Statistic type for inference (See Eklund et al. 2016) | Statistical inference was performed using correlation analyses for similarity measures and ordinary least squares (OLS) or linear mixed models for regression analyses to test age and sex effects.

Correction | False discovery rate (FDR) correction was applied to account for multiple comparisons.

Models & analysis

n/a	Involved in the study
☐	☒ Functional and/or effective connectivity
☒	☐ Graph analysis
☐	☒ Multivariate modeling or predictive analysis

Functional and/or effective connectivity | Pearson correlation

Graph analysis | *Report the dependent variable and connectivity measure, specifying weighted graph or binarized graph, subject- or group-level, and the global and/or node summaries used (e.g. clustering coefficient, efficiency, etc.).*

Multivariate modeling and predictive analysis | Multiple analyses were performed and are described in the Methods section. The two main analyses involved feature extraction and structure-function coupling. For microstructural features, voxel-wise measures including voxel count, magnetization transfer ratio (MTR), fractional anisotropy (FA), radial diffusivity (RD), mean diffusivity (MD), and axial diffusivity (AD) were extracted. For functional features, connectivity measures and 22 time-series features were extracted. Structure-function coupling was assessed using correlation analyses between corresponding microstructural and functional features. Finally, ordinary least squares (OLS) models were employed to examine the effects of age and sex.
