# Supplementary material for: Spinal cord structural and functional architecture and its shared organization with the brain across the adult lifespan
Source: Nat Commun. 2026 Apr 16;17:5269. doi: 10.1038/s41467-026-71963-2 (PMC13265777; doi:10.1038/s41467-026-71963-2)
Supplement: Supplementary file 3 — Transparent Peer Review file [file 41467_2026_71963_MOESM3_ESM.pdf]

nature portfolio

Peer Review File

Spinal cord structural and functional architecture and its shared organization with the brain across the adult lifespan

Corresponding Author: Dr Caroline Landelle

This file contains all reviewer reports in order by version, followed by all author rebuttals in order by version.

Version 0:

Reviewer comments:

Reviewer #1

(Remarks to the Author)
This study aimed to characterize cervical spinal cord microstructure and cortico-spinal function across lifespan. Authors obtained spinal architecture, structure-function coupling, and age-related alterations from multimodal spinal MRI datasets. Interestingly they applied brain and cord imaging together to explore the shared CNS-wide aging trajectories. Overall I do think that addressing spinal-cerebral integration is novel as yet underexplored in aging neuroscience. However, I do remain some major concerns.

1. Despite some considerable methodological strengths, the study suffers from a critical flaw, small sample size, which I think will significantly impact the interpretation of lifespan related findings. Only 67 participants (covers 20 to 80 years old, assumes one participant per year) had T2s, T1w, and fMRI imaging, of whom 55 had also MT and/or DWI data. Out of these datasets, many features were calculated such as FC, dyn, FA, MD, MTR etc. Associations with age was determined, elastic net regression models were used for predictions, and even sex differences were observed. So many analysis with such limited sample and external validation datasets would be welcomed to generalize findings. Although authors tested correlations between different methods for validation, bootstrap methods might still be needed to assess the stability of selected features across the datasets.
2. Authors claimed that the finding of somatosensory pathway vulnerability with aging aligns with clinical observations of age-related sensory decline, while there were not sensory related testing in the current cohorts, such as a simple QST test for sensory/pain threshold. Also maybe in the future study diseases with accelerating aging or sensory decline could be included as well to prove cord structure and functional architecture changes accordingly.
3. Authors employed two elastic net regression models incorporating all extracted features to predict participants' age. While cross-validation is used for hyperparameter tuning and model evaluation, no external validation dataset is included. This raises concerns about the generalizability of the models to unseen data. Also without permutation testing, it is difficult to determine whether the model's performance metrics (e.g., R^2, MAE) are truly indicative of a meaningful relationship between the input features and age or simply the result of overfitting or spurious correlations.
4. When examining the six specific connection types (i.e., dorso-dorsal, ventro-ventral, left dorso-ventral, right dorso- ventral, left cross-horns, right cross-horns), inter-segmental connectivity significantly increased with age across all connections except the dorso-dorsal? This is quite difficult to follow as previously authors found the somatosensory pathway vulnerability with aging which suggesting sensory decline, but DD connectivity did not reflect this accordingly.
5. Authors defined a cortical-subcortical-spinal cord gradient of intrinsic dynamics, which I am not sure about its relationship with age? Or simply can try the cortico-spinal resting state connectivity pattern discovered in Vahdat et al. Plos Biology 2020 paper?

(Remarks on code availability)

Reviewer #2

(Remarks to the Author)
I co-reviewed this manuscript with one of the reviewers who provided the listed reports. This is part of the Nature Communications initiative to facilitate training in peer review and to provide appropriate recognition for Early Career

Researchers who co-review manuscripts.

This manuscript reports a multimodal MRI study of cervical spinal cord structure and function across the adult lifespan, and relates these spinal measures to brain organization. The authors quantify age-related changes in gray and white matter morphometry and microstructure, derive resting-state functional connectivity between spinal regions, and extract dynamic properties of spinal BOLD time series. They then assess structure–function coupling within the cord and examine how spinal microstructural, functional, and dynamic metrics, and their age dependencies, relate to brain gray matter, functional connectivity, and large-scale networks. The central claim is that aging is associated with convergent, somatosensory-dominant microstructural decline, reduced functional specificity ("dedifferentiation"), and increased spontaneous activity in both spinal cord and brain.

The study addresses an important question: how spinal cord structure and function are organized in vivo across adulthood, and how this relates to known principles of brain organization. The dataset is strong: cervical spinal microstructure, spinal resting-state fMRI, and whole-brain imaging were all acquired in the same participants with a setup adapted to the cord. Bringing several spinal metrics together in one framework is a clear strength, as is the effort to link these spinal measures to brain networks. The focus on somatosensory-dominated aging effects, structure–function coupling, and dynamic properties of spinal activity is original and adds a useful systems-level perspective.

Overall, this is a novel and technically demanding study with clear potential impact for spinal and systems neuroscience. The acquisition and analysis framework are solid, but the reported age effects appear relatively focal and tract-dependent, and it would be helpful to relate them more explicitly to prior qMRI work that has reported widespread age-related microstructural differences in the brain. At the same time, several conclusions rely on complex derived metrics and on a cross-sectional sample that spans a broad age range, so the interpretation needs to be cautious.

Major comments

1. The spinal parcellation is based on segment × quadrant (dorsal/ventral, left/right) rather than tract-specific ROIs (for example dorsal columns or corticospinal tract). It would be helpful if the authors could elaborate on this design choice. For instance, were tract-level ROIs considered but rejected due to SNR, registration, or anatomical variability, and how might the chosen parcellation influence the interpretation of somatosensory-dominant versus motor-dominant effects?
2. Since the main claims concern convergent age-related changes across structure, connectivity, and dynamics, it would be helpful to consider a simple unified model that includes a small set of key spinal and brain metrics in a single framework, or to briefly explain why such a joint model is not feasible or appropriate. More generally, the age modelling across spinal and brain measures could be described and justified more clearly. Several analyses include linear age terms for spinal microstructure, SpiFC, SpiDyn, and brain–spine relationships. I suggest adding a short paragraph or compact table that specifies, for each model, how age was entered (for example continuous linear term or age groups), which covariates were included (for example sex, motion, tSNR), and whether any non-linear terms were used for inference or only for visualization.
3. The claims about structure–function coupling in the cord and "CNS-wide" organization are interesting, but different types of evidence are currently discussed together. It would help to more clearly separate (a) structure–function coupling within the cord (for example Fig. 3), (b) direct relationships between spinal and brain measures (for example Fig. 4), and (c) parallel age effects seen separately in brain and cord and interpreted as CNS-wide patterns. Making explicit which analyses support each level would strengthen the argument. I would also suggest using terms such as "CNS-wide organization" or "shared CNS-wide aging mechanisms" only when that specific level of evidence is meant.
4. For the brain, the analyses focus on gray-matter morphometry and resting-state functional connectivity. Could the authors clarify whether diffusion or additional quantitative MRI measures were available and, if so, why the analysis was restricted to GM metrics? A short statement on available modalities and the rationale for focusing on the chosen measures would help the reader understand the scope and limits of the brain–spine comparisons.
5. For spinal structure, several quantitative measures (T2-based voxel counts, MTR, FA, RD, etc.) are combined into a multivariate feature vector per region and then summarized as morphometric similarity. While this is consistent with previous brain morphometric similarity work, it would help if the authors could (i) clearly summarize which individual metrics show the strongest age effects in dorsal versus ventral regions and (ii) comment on whether the observed pattern of morphometric similarity and its age dependence is robust across individual metrics or is driven mainly by a subset of them. Even a brief figure or table in the Supplementary Material could clarify this.

Minor comments

1. There are a few typos and grammar errors (for example "ans show increase in GM", "GM voxels count"), so a careful proofread would be useful.
2. In the Fig. 4 caption, CSA is reported "in mm^3", but CSA is an area measure and should be "in mm^2".
3. The QC information is spread across the Methods, Supplementary Figs. S2–S3, and Table S1, which makes it hard to get an overview of data quality. A short QC summary in the main text that brings together FD and tSNR values (and any age effects), Dice for GM and main tracts, and the number of exclusions per modality, plus a statement on whether any QC metrics were tested as covariates, would make the results easier to interpret.
4. In the "Time series denoising" and "Functional connectivity analyses" sections, you describe RETROICOR, CSF CompCor, DCT terms, and slice-wise motion regressors, and then compute FC on denoised, z-scored time series. It is not fully clear whether the same nuisance model was used for brain and spinal cord, or whether additional steps were applied for brain FC. A brief clarification of the denoising model for each (and any differences between them) would make the FC and SpiDyn results easier to follow.
5. You report tSNR values for spinal cord and brain (for example mean spinal tSNR 22.16 ± 2.0 with no significant age effect; QC section and Fig. S2C), but it is not stated whether tSNR was used as a criterion for excluding participants or runs. Clarifying whether any tSNR thresholds were applied, or confirming that tSNR was only used descriptively, would make the QC procedure more transparent.
6. In the Methods section "Spinal dynamic features (SpiDyn)" and the Supplementary figures, you describe extracting time-series features (catch-22, ALFF, standard deviation), excluding those with a dominant single value (f_dom), and later

applying PCA with n = 10 components. It would help to summarize, in one place, how many features remain after exclusion and which of these are actually entered into the age analyses.
It would also be helpful to cite related qMRI and CNS aging work, in particular Callaghan et al., Neurobiology of Aging 2014, and Labounek et al., Imaging Neuroscience 2025.

(Remarks on code availability)

Reviewer #3

(Remarks to the Author)
I co-reviewed this manuscript with one of the reviewers who provided the listed reports. This is part of the Nature Communications initiative to facilitate training in peer review and to provide appropriate recognition for Early Career Researchers who co-review manuscriptsM

(Remarks on code availability)
The GitHub repo is clearly linked to the manuscript and lists the main dependencies and versions, with a sensible structure (code, configs, figure notebooks, templates). Scripts and notebooks for preprocessing and Figures 1–4 are provided, but the README notes that some parts (especially supplementary material) are still being uploaded and there is no single step-by-step pipeline. I have not installed or run the code, so I cannot judge full reproducibility, but it seems usable for experienced users and would be easier to use with a more detailed “how to run” guide.

Reviewer #4

(Remarks to the Author)
In this paper, Landelle et al. investigates the structural and functional changes along the whole neuroaxis in a cohort across the lifespan. The paper and data are definitely of high interest and timely. The figures are beautiful. And I can see that, authors did a lot of work to acquire and analyze the data with novel methods. I do have major and minor comments.

Major: My major comments relate to interpretability, clarity of age-related findings, and methodological transparency.
My main concern: The authors have done rigorous work. However, the main take-away are not clear. I would love to see clear and understandable age effects on each metric as shown in Supplementary Figure S5. I further specified this concern below.
Results:
1. I would like to see a histogram of Age across people. It is unclear how the Age is distributed across the sample.
Also, please provide a missingness analysis, are the subjects who have missing data, older people? If this is the case, is not this a confound? I believe the Supp. Fig S4 is incorrect (B and C are identical), the N is inconsistent.

2. Although very interesting, the paper is very dense and hard to follow. I appreciate the rigor and novelty in the analysis, the main premise of the paper is to demonstrate the effects of aging across CNS. However, this premise gets very hard to clearly see with the metrics like “morphometric similarity” and “SpiDyn”. What does morphometric similarity mean, how can one interpret it? What do SpiDyn features mean and how can we interpret the similarity between them. It is abstraction of an abstract feature. I really would like to see Supplementary Figure 5 in the main text and the more complex metrics can be introduced more in detail later (and some of them can be moved to supplement).

3. Similar to this, SpiDyn, is an interesting and novel concept, however, it makes it harder to follow the paper and reduces interpretability. Have those metrics been used previously (in brain literature) – if yes, how have they been interpreted? I am also not sure about the “Dyn” in the name. ALFF has not been considered a “dynamic” resting-state fMRI measure. Additionally, I can see that the metrics also include mean and SD. How is the mean showing the dynamic properties of that ROI? Since the SpiDyn is highly correlated with spiFC, I do not think it adds that much to the manuscript (particularly considering the interpretability). These should be moved to supplement and age effects Age Vs spiFC can be shown clearly.

4. Although I find the figures aesthetically very appealing, the coloring is confusing. Please make sure it is consistent between the figures and always indicates the same thing. For instance,in Figure 1D, the t value map has the same color scheme for age in Figure 4A (same for supplementary figures).

5. Please make sure Figure legends are informative and information rich in a way that what the figures show can be understood without going back to Methods. Especially for some figures, I found it hard to understand what exactly each plot shows. For instance, it should be specified in the legend that figure 1C shows group-averaged similarity matrix. Sometimes the FDR corrected values are depicted as (*); see Figure 2E as an example, and sometimes not (Figure 4D). Please make sure these are consistent across Figures, and the same symbol always signifies the same thing/value.

6. Following up on that, it is unclear what ROI has been used for which analysis? For spiFC, was it gray matter only or quadrant? If quadrant, Figure X is misleading. If gray matter for spiFC and whole quadrant for spiDyn, why is the discrepancy?

7. Which analysis was done in template space, which analysis was done native space? It is very hard to follow. Please make it clearer.

8. Related to that, some of the scatter plots such as Figure 3 show multiple points for each subject. What exactly do these shows? How many points from each subject are there? How has this been this accounted in analysis (a mixed-effects models needs to be used not to artificially inflate the p-values)?

9. Intra vs inter network results are interesting, but could not it be mainly due to the spatial proximity? How many EPI slices were within one segment? Was the analysis done in native space or template space?

10. Please clarify spinal cord GM volume calculation. Native space, template space? Is it normalized?

Over-statements in Discussion:
11. The authors conclude that the findings “lay the groundwork for early biomarkers of sensorimotor decline”. This oversteps the evidence – the study is cross-sectional in healthy adults and did not track actual sensorimotor decline or test predictive power for future impairment. No behavioral measures of sensorimotor performance were included, so there is no direct link between the MRI metrics and functional decline. Calling these measures “early biomarkers” is speculative. To support such a claim, longitudinal data showing that these imaging measures predict sensorimotor decline (or at least correlate with current performance deficits) would be needed.

12. The authors claim to provide the “first evidence that dedifferentiation extends across the CNS”, referring to the age-related increase in inter-network functional connectivity in both brain and spinal cord. It is true the data show a trend toward less network-specific connectivity with age (i.e. more between-network connectivity, interpreted as dedifferentiation) in the spinal cord, mirroring a known brain aging effect. However, this interpretation may be somewhat overstated. First, the dedifferentiation effect size appears modest. For example, the reported increase in inter-network connectivity with age, though significant, is not very large in magnitude (the authors describe it as a “weak but significant” correlation in the spinal cord). Additionally, not all aspects of network organization followed the dedifferentiation pattern uniformly. In the brain, some networks (visual, dorsal attention, cerebellar, etc.) actually showed increased intra-network connectivity with age, which is opposite to a loss of specialization. This nuanced result suggests the story is more complex than a simple global dedifferentiation.

13. The authors observed that the left dorsal quadrant of the cervical cord (which corresponds to afferents from the non-dominant arm in this all right-handed sample) exhibited the most pronounced aging effects, and they speculate that “reduced limb use may accelerate aging in its corresponding spinal territory”. This is an interesting hypothesis, but it is presented rather strongly despite being unsupported by direct evidence in the study. No data on limb use frequency, physical activity, or motor behavioral decline in the left vs. right side were collected. Thus, attributing the greater left-side changes to disuse is speculative. The asymmetry could have other explanations.

Minor:
14. There are some typos across the manuscript, please correct them.

15. References are unorganized. Sometimes they are numbered and sometimes they are in-text APA style (with authors surname and year).

16. Why are the small ROI to ROI FC matrices (RV-RV etc) not symmetric?

17. I understand that one cannot try every method. But what if the age effects were not linear? Did authors explore that?

(Remarks on code availability)
Code is there, seems well organized and commented. I understand authors may not want to share data due to ongoing analysis. Are there any example derivatives files (from one subject) which can be added to follow the code?

Version 1:

Reviewer comments:

Reviewer #1

(Remarks to the Author)
I am happy with the detailed responses from authors and have no further comments.

(Remarks on code availability)

Reviewer #2

(Remarks to the Author)
The authors did a great job in replying to my colleagues queries.

(Remarks on code availability)

Reviewer #3

(Remarks to the Author)
I co-reviewed this manuscript with one of the reviewers who provided the listed reports. This is part of the Nature Communications initiative to facilitate training in peer review and to provide appropriate recognition for Early Career Researchers who co-review manuscripts.

(Remarks on code availability)

Reviewer #4

(Remarks to the Author)
I thank authors for their detailed responses and for implementing the suggestions!

(Remarks on code availability)

Open Access This Peer Review File is licensed under a Creative Commons Attribution 4.0 International License, which permits use, sharing, adaptation, distribution and reproduction in any medium or format, as long as you give appropriate credit to the original author(s) and the source, provide a link to the Creative Commons license, and indicate if changes were made.
In cases where reviewers are anonymous, credit should be given to 'Anonymous Referee' and the source.
The images or other third party material in this Peer Review File are included in the article's Creative Commons license, unless indicated otherwise in a credit line to the material. If material is not included in the article's Creative Commons license and your intended use is not permitted by statutory regulation or exceeds the permitted use, you will need to obtain permission directly from the copyright holder.
To view a copy of this license, visit https://creativecommons.org/licenses/by/4.0/

We thank the reviewers for their enthusiasm toward our study and for their insightful comments and suggestions, which have substantially improved the quality and clarity of the manuscript. We have carefully addressed all comments and questions raised by the reviewers point-by-point. Our responses are provided below in blue, and text that has been modified or added to the manuscript is shown in **bold red** in this document.

Thanks to reviewer comments, during the revision process, we identified several collateral inconsistencies that required correction. The following changes have been implemented in the revised manuscript:

1. In some figure captions and tables, the statistical model was inconsistently reported (OLS vs. LMM), and in a few cases, the incorrect model was initially applied. These issues have been corrected, and all statistical analyses were rerun, including Bootstrapping to assess the stability of the results. A new paragraph in the method section and the table S2 explicitly state which model was used for each analysis. This led to numerical changes in reported statistics, but did not alter the overall pattern of results or the main conclusions. All statistical tables and figures have been double-checked and updated accordingly.

2. The estimation of white-matter voxel counts was previously affected by an implementation error, in which an outdated processing step was inadvertently retained. We corrected this issue and reran the analysis. As a consequence, microstructural morphometric similarity matrices and all related statistics were recomputed. With the corrected implementation, white-matter measures now show age-related effects that were not detected previously. Importantly, this correction strengthens rather than changes our original conclusions, particularly regarding pronounced age-related effects in the left dorsal horn.

Reviewer 1

This study aimed to characterize cervical spinal cord microstructure and cortico-spinal function across lifespan. Authors obtained spinal architecture, structure-function coupling, and age-related alterations from multimodal spinal MRI datasets. Interestingly they applied brain and cord imaging together to explore the shared CNS-wide aging trajectories. Overall I do think that addressing spinal-cerebral integration is novel as yet underexplored in aging neuroscience. However, I do remain some major concerns.

1. Despite some considerable methodological strengths, the study suffers from a critical flaw, small sample size, which I think will significantly impact the interpretation of lifespan related findings. Only 67 participants (covers 20 to 80 years old, assumes one participant per year) had T2s, T1w, and fMRI imaging, of whom 55 had also MT and/or DWI data. Out of these datasets, many features were calculated such as FC, dyn, FA, MD, MTR etc. Associations with age was determined, elastic net regression models were used for predictions, and even sex differences were observed. So many analysis with such limited sample and external validation datasets would be welcomed to generalize findings. Although authors tested correlations between different methods for validation, bootstrap methods might still be needed to assess the stability of selected features across the datasets.

We understand the reviewer's comment about the sample size and validation methods. To the best of our knowledge, this dataset represents the largest cohort to date combining

multimodal spinal cord MRI (structural, microstructural, functional, and dynamic measures) with brain imaging acquired in the same individuals across a wide adult age range. While we agree with the reviewer that the generalization of the findings from this sample size may be problematic given the wide range for age, we believe that the suggested bootstrap approach may mitigate it. In addition, given that spinal cord imaging remains a technically challenging and relatively young field compared to brain imaging, where large-scale datasets already exist (for instance, not all MRI centers are equipped with dedicated coils for spinal fMRI or have the technical expertise required to develop and implement such sequences), it is not feasible, at the moment to perform a direct comparisons with large spinal cord cohorts as they are currently non-existent. In this regard, we believe that our results, even if the sample size is limited, may provide a basis for such comparisons for future studies.

We believe that the internal validity of our results may benefit from the fact that the acquisition protocol was intentionally comprehensive, prioritizing rich multimodal characterization within individuals. This approach allowed us to investigate convergence across complementary features, thereby strengthening the interpretation of the findings despite the modest sample size. We hope that continued and more systematic acquisition of spinal cord MRI following our protocol, potentially through multi-site initiatives, will enable larger cohorts in the future and allow further validation and extension of the present findings.

Following the reviewer's recommendation, we have now applied nonparametric bootstrap resampling (1,000 iterations) to all OLS and LMM analyses to assess the stability of the results. In each iteration, participants were sampled with replacement and the regression coefficients recalculated. From the resulting empirical distributions, 95% confidence intervals were derived for each coefficient, providing an estimate of uncertainty and stability.
Here is an illustration of the analysis for the WM voxel counts.

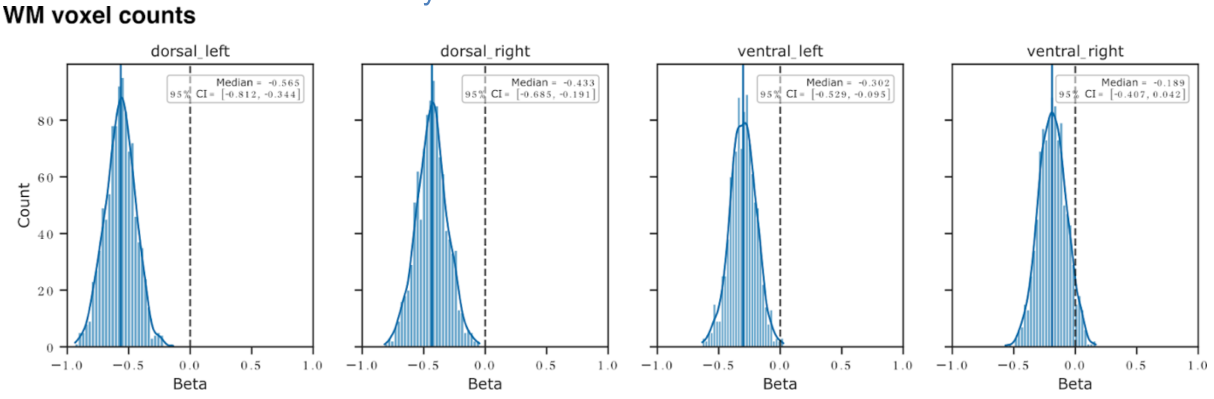

Results for all metrics are reported in supplementary Tables 3-7 and 10-12, and the bootstrap procedure is described in detail in the Methods section:

- Line 980: "**We performed bootstrap resampling with 1000 iterations for all OLS and LMM analyses to assess the stability of the results. This non-parametric method generates new datasets by randomly sampling observations from the original dataset with replacement, so that some data points may appear multiple times while others may be omitted in each resampled dataset. These resampled datasets are then used to create an empirical distribution of the model's beta values. For each model, the resulting bootstrap distributions were summarized using the median beta values and 95% confidence intervals.**"
- Line 173: **"To assess the stability of these estimates, we performed bootstrap resampling (1,000 iterations), and the resulting 95% confidence intervals are reported in Supplementary Table S2.**"

2. Authors claimed that the finding of somatosensory pathway vulnerability with aging aligns with clinical observations of age-related sensory decline, while there were not sensory

related testing in the current cohorts, such as a simple QST test for sensory/pain threshold. Also maybe in the future study diseases with accelerating aging or sensory decline could be included as well to prove cord structure and functional architecture changes accordingly.
We thank the reviewer for this insightful comment. We agree that sensory testing was not included in the current cohort, and this represents a limitation of our study. We have added a discussion of this limitation and suggested that future studies could incorporate sensory assessments, such as quantitative sensory testing, and investigate populations with accelerated aging or sensory decline to further evaluate changes in cord structure and functional architecture.

Line 509: "To better understand these region-specific changes with aging along the sensory-motor axis, future studies should investigate spinal cord function beyond resting-state assessments by incorporating motor tasks, somatosensory-stimuli paradigms **or sensorimotor decline assessment**, allowing a more comprehensive evaluation of somatosensory and motor alterations associated with aging".
Line 610: "It is important to recognize the complementary insights offered by task-based paradigms, **and sensorimotor decline assessment** can more directly probe functional specialization and recruitment patterns during sensorimotor engagement."

3. Authors employed two elastic net regression models incorporating all extracted features to predict participants' age. While cross-validation is used for hyperparameter tuning and model evaluation, no external validation dataset is included. This raises concerns about the generalizability of the models to unseen data. Also without permutation testing, it is difficult to determine whether the model's performance metrics (e.g., R^2, MAE) are truly indicative of a meaningful relationship between the input features and age or simply the result of overfitting or spurious correlations.

We thank the reviewer for this important comment. We agree that external validation is the gold standard for assessing model generalizability. In the current study, our sample size did not allow for a fully independent test set. However, at the reviewer's suggestion we sought to ensure that our age prediction results were not driven by overfitting or spurious correlations. Thus, we have now implemented a permutation testing procedure. Specifically, we randomly shuffled the participants' age labels 1000 times and re-fitted the ElasticNet models for each permutation, generating null distributions of R^2 and MAE values. The observed model performance for both dorsal and ventral ROIs exceeded the values expected by chance ($p < 0.001$), confirming that the models capture meaningful relationships between spinal cord microstructural features and age. These additional analyses provide robust evidence for the validity of our predictive models.

We have now added a supplementary figure S7, illustrating the permutation test results.

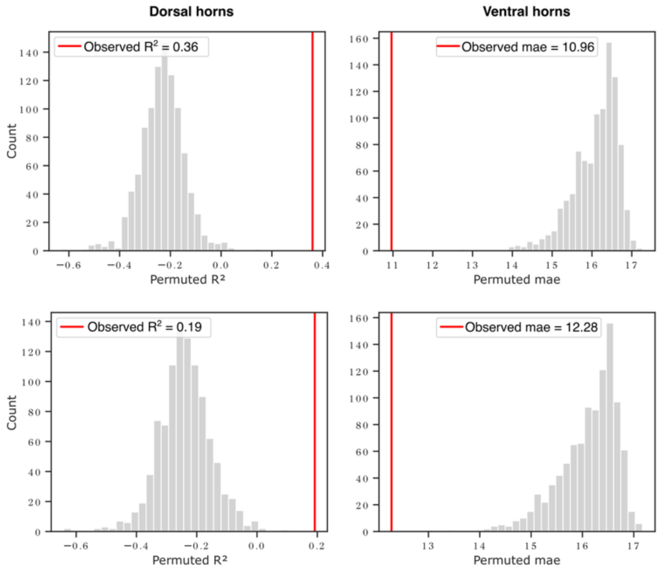

Fig S7 | Permutation tests on age prediction models. The permutation procedure consisted of randomly shuffling participants' age labels 1000 times and refitting the ElasticNet prediction models for each permutation, generating null distributions of R^2 and MAE values. This was performed separately for models applied to the dorsal and ventral horns. Gray bars represent the distribution of R^2 (left) or MAE (right) values across the 1000 iterations, and the red vertical line indicates the observed value. The observed model performance for both dorsal and ventral ROIs exceeded the values expected by chance ($p < 0.001$). MAE: Mean Absolute Error; R^2 : proportion of variance in chronological age explained by the model predictions; ROI: Region Of Interest.

We have added the following details in the main manuscript:

- Line 744: "For each iteration, the model produces regression weights for each predictor, *i.e.* each feature, corresponding to the importance of each feature in predicting age. **To ensure that our age prediction models were robust and not driven by spurious correlations, we performed permutation testing (1,000 permutations) by randomly shuffling age labels across participants. ElasticNet models were re-fitted for each permutation, generating null distributions of R^2 and MAE values."**
- Line 188: **Permutation testing (1000 iterations with shuffled age labels) confirmed that both models performed significantly above chance (dorsal: $p\_perm\_R^2 < 0.001$, $p\_perm\_MAE < 0.001$; ventral: $p\_perm\_R^2 < 0.001$, $p\_perm\_MAE < 0.001$; Fig. S7)**

4. When examining the six specific connection types (i.e., dorso-dorsal, ventro-ventral, left dorso-ventral, right dorso- ventral, left cross-horns, right cross-horns), inter-segmental connectivity significantly increased with age across all connections except the dorso-dorsal? This is quite difficult to follow as previously authors found the somatosensory pathway vulnerability with aging which suggesting sensory decline, but DD connectivity did not reflect this accordingly.

We thank the reviewer for this remark. We agree that the description can be difficult to follow, so we would like to clarify that, to the best of our knowledge, anatomically, there are no spinal circuits that support direct communication between the two dorsal horns, and therefore, functional connectivity is expected to be non-existent or weak. In contrast, the dorsal horn communicates extensively with the ipsilateral ventral horns to relay somatosensory signals to the motor command (sensorimotor integration) and with the contralateral ventral horns *via* commissural interneurons to support cross-limb coordination. Consequently, if aging affects the dorsal horn, we would primarily expect changes in dorso-ventral connectivity rather than within the two dorsal horns. This hypothesis is consistent with our results intra and inter-connectivity results, which show significant age-related

changes only in functional connectivity between the dorsal and ventral horns. These findings likely reflect the age-related changes in sensory processing at the spinal level that influence local sensorimotor integration.

The rationale has been added in the main text:

- Line 285: The increase in inter-segmental connections suggests a decline in the specificity of spinal connectivity with age. **In parallel,** selective increases in intra-segmental **dorso-ventral** FC, particularly those driven by the left dorsal horn**, likely reflect age-related changes in sensory modulation of motor neurons**.
- Line 485: **In addition,** increased **functional** connectivity **was found between the dorsal and ventral horns that** primarily **involves** the left dorsal quadrant (*i.e.,* left dorso-ventral and left dorsal-right ventral FC)**. This pattern suggests that aging may alter spinal sensorimotor integration, as local connectivity in the spinal cord is largely driven by dorso-ventral interactions where sensory signals modulate motor neuron activity.**
- Line 488**: Ipsilateral dorso-ventral connectivity, reflecting the relay of somatosensory signals to motor circuits (sensorimotor integration), may be sensitive to age-related alterations in spinal reflex loops[59]. Changes in connectivity between the left dorsal horn and the contralateral ventral horn may, in turn, reflect modifications in cross-communication via commissural interneurons involved in cross-limb coordination[60] .**

5. Authors defined a cortical-subcortical-spinal cord gradient of intrinsic dynamics, which I am not sure about its relationship with age? Or simply can try the cortico-spinal resting state connectivity pattern discovered in Vahdat et al. Plos Biology 2020 paper?
We thank the reviewer for this thoughtful suggestion. It is important to emphasize that each of our analyses begins with a general characterization that does not test age effects. Accordingly, the paper has two complementary aims: first, to provide a comprehensive description of the structural and functional organization of the spinal cord that is common and transcends age, and second, to investigate how this organization is modulated by aging. In this context, the primary goal of the present analysis was not to characterize aging effects *per se*, but to demonstrate that spinal intrinsic dynamics (SpiDyn) are meaningfully organized along the CNS axis and embedded within the well-described sensorimotor-to-associative gradient. We believe this result is important in its own right, as it situates the spinal cord within a CNS-wide organizational framework that is often overlooked in systems neuroscience. In parallel, we explicitly test the effect of age on each SpiDyn metric at both brain and spinal cord levels, allowing us to identify which features of spontaneous activity are preserved across the lifespan and which are selectively altered by aging.

- The rationale of the analysis was added in the result section (line 426): "**To situate spinal intrinsic dynamics within a broader CNS organizational framework**, we investigated the topographical organization of time-series dynamics across each level of the CNS."

We agree that investigating cortico-spinal resting-state functional connectivity patterns, such as those reported by Vahdat et al. (PLOS Biology, 2020), would be a valuable extension of the present work. However, we chose not to pursue these analyses here due to the already extensive scope of the study and the additional methodological challenges involved, including the definition of appropriate cortical and spinal regions of interest and the interpretation of their connectivity. We now explicitly acknowledge this direction as an important avenue for future studies in the Discussion.

- The discussion was updated Line 567: "We further examine local time-series dynamics using a principal functional gradient approach, to characterize the macroscale organization of intrinsic CNS activity macroscale organization of intrinsic CNS activity[1] **independently of aging effects. This analysis revealed two continuous patterns of intrinsic organization**.
- The discussion was updated Line 564: **Future studies could explore whether these spinal connectivity changes are linked to cortical networks and *vice versa* by examining cortico-spinal resting-state connectivity patterns** [20,22].

Reviewer 2

I co-reviewed this manuscript with one of the reviewers who provided the listed reports. This is part of the Nature Communications initiative to facilitate training in peer review and to provide appropriate recognition for Early Career Researchers who co-review manuscripts.

This manuscript reports a multimodal MRI study of cervical spinal cord structure and function across the adult lifespan, and relates these spinal measures to brain organization. The authors quantify age-related changes in gray and white matter morphometry and microstructure, derive resting-state functional connectivity between spinal regions, and extract dynamic properties of spinal BOLD time series. They then assess structure–function coupling within the cord and examine how spinal microstructural, functional, and dynamic metrics, and their age dependencies, relate to brain gray matter, functional connectivity, and large-scale networks. The central claim is that aging is associated with convergent, somatosensory-dominant microstructural decline, reduced functional specificity ("dedifferentiation"), and increased spontaneous activity in both spinal cord and brain.
The study addresses an important question: how spinal cord structure and function are organized in vivo across adulthood, and how this relates to known principles of brain organization. The dataset is strong: cervical spinal microstructure, spinal resting-state fMRI, and whole-brain imaging were all acquired in the same participants with a setup adapted to the cord. Bringing several spinal metrics together in one framework is a clear strength, as is the effort to link these spinal measures to brain networks. The focus on somatosensory-dominated aging effects, structure–function coupling, and dynamic properties of spinal activity is original and adds a useful systems-level perspective.
Overall, this is a novel and technically demanding study with clear potential impact for spinal and systems neuroscience. The acquisition and analysis framework are solid, but the reported age effects appear relatively focal and tract-dependent, and it would be helpful to relate them more explicitly to prior qMRI work that has reported widespread age-related microstructural differences in the brain. At the same time, several conclusions rely on complex derived metrics and on a cross-sectional sample that spans a broad age range, so the interpretation needs to be cautious.

Major comments

1. The spinal parcellation is based on segment × quadrant (dorsal/ventral, left/right) rather than tract-specific ROIs (for example dorsal columns or corticospinal tract). It would be helpful if the authors could elaborate on this design choice. For instance, were tract-level ROIs considered but rejected due to SNR, registration, or anatomical variability, and how might the chosen parcellation influence the interpretation of somatosensory-dominant versus motor-dominant effects?

We thank the reviewer for this important question. The spinal parcellation was based on segment × quadrant (dorsal/ventral, left/right), which include both GM and WM tissue, but complementary analyses were also restricted to the white matter tracts (Fig. S6). Spontaneous functional activity is mainly driven by gray matter rather than white matter tracts, while some quantitative MRI metrics are more sensitive to white matter. Combining gray and white matter within the same quadrants ensures consistency for structure–function coupling analyses. This approach balances anatomical interpretability with methodological reliability. In addition, it minimizes the partial volume effect between tissue types.
Importantly, complementary analyses focusing specifically on white matter tracts were also performed and are provided as supplementary data, offering additional support for our

findings (Fig. S6). These results confirm that the white matter dorsal column is more affected by aging than the lateral funiculi (corticospinal tract) or ventral funiculi Combining both quadrant analyses and tract-specific analyses confirms that the somatosensory pathways were more affected by aging compared to motor pathways.

A more detailed justification of the ROI choice has now been included in the Methods section.

- Line 112: "**Each parcel includes both gray and white matter, ensuring comparability between structural and functional analyses while minimizing the partial volume effects between tissue types.**"
- Line 704: "**Each quadrant includes both gray and white matter, as some quantitative MRI metrics are sensitive to microstructural properties of both tissue types. Complementary tract-specific analyses (dorsal columns, ventral funiculi and lateral funiculi) were also conducted for MT and DWI metrics.**"
- Line 855: "**Each quadrant included both gray and white matter, ensuring comparability with structural analyses while minimizing the partial volume effects between tissue types.**"

2. Since the main claims concern convergent age-related changes across structure, connectivity, and dynamics, it would be helpful to consider a simple unified model that includes a small set of key spinal and brain metrics in a single framework, or to briefly explain why such a joint model is not feasible or appropriate. More generally, the age modelling across spinal and brain measures could be described and justified more clearly. Several analyses include linear age terms for spinal microstructure, SpiFC, SpiDyn, and brain–spine relationships. I suggest adding a short paragraph or compact table that specifies, for each model, how age was entered (for example continuous linear term or age groups), which covariates were included (for example sex, motion, tSNR), and whether any non-linear terms were used for inference or only for visualization.
We thank the reviewer for this thoughtful suggestion. The primary aim of this study was to provide a comprehensive characterization of spinal cord and brain organization across structural, functional connectivity, and dynamical domains, and to examine how each of these dimensions is modulated by age. This choice was motivated by the distinct acquisition protocols and data availability for the brain and spinal cord, notably the availability of only T1-weighted structural imaging for the brain, whereas multiple quantitative microstructural metrics were available for the spinal cord. As a result, the structural measures were not directly comparable across CNS levels, making a unified multimodal model difficult to interpret. Importantly, this approach enabled us to identify the specific spinal and brain metrics and regions most sensitive to age-related effects, which can serve as a principled basis for future studies aimed at building integrated, multivariate models across modalities

To improve clarity of the statistical model, we have added a dedicated paragraph at the end of the Methods section describing the age-modelling strategy across all analyses, along with a summary table S2 in the Supplementary Materials specifying, for each model, how age was entered (continuous linear term), and which covariates were included. Briefly, age was modelled as a continuous linear predictor in all analyses; sex was included as a covariate. Motion was not entered explicitly as a covariate, as its effects were addressed during preprocessing for functional analyses through slice-wise motion correction and regression of motion-related nuisance regressors during denoising. Temporal SNR (tSNR) was used for quality control and descriptive purposes only and was not included in the statistical models.
Thanks to this comment, we realized that the use of distinct statistical models was not always clear and, in some cases, not fully appropriate. Accordingly, we reran the statistical analyses using a more rigorous approach, employing either ordinary least squares (OLS) or linear mixed models (LMM) as appropriate. The Methods section has been updated to reflect these changes, including a new paragraph justifying the choice of model, and a corresponding summary is provided in Supplementary Table S2. Statistical results in the main text and supplementary materials have been updated accordingly.

Line 968: "Statistical analyses

Across all analyses, age was modelled as a continuous linear predictor. Separate regression models were applied to spinal microstructural metrics, spinal functional connectivity (SpiFC), and intrinsic spinal dynamics (SpiDyn), at both the spinal cord and brain levels. Sex was included as a covariate in all models. When participants contributed multiple observations to a given analysis, participant identity was included as a random effect, resulting in two types of models: ordinary least squares (OLS) regression with age and sex as fixed effects, and linear mixed-effects models (LMMs) with age and sex as fixed effects and participant as a random effect. A summary of age terms, covariates, and modelling choices for each analysis is provided in Supplementary Table S2. We performed bootstrap resampling with 1000 iterations for all OLS and LMM analyses to assess the stability of the results. This non-parametric method generates new datasets by randomly sampling observations from the original dataset with replacement, so that some data points may appear multiple times while others may be omitted in each resampled dataset. These resampled datasets are then used to create an empirical distribution of the model's beta values. For each model, the resulting bootstrap distributions were summarized using the median beta values and 95% confidence intervals."

	Test	X	Y	Cov	Random	Model	Multiple correction
**Spinal cord**							
**Morpho**	Intra vs. inter Fig. 1C	Intra Similarity	Inter Similarity	-	-	Paired t-test	-
	Age-effect Fig. 1D Fig. S5	Age	Morpho. metric	Sex	IDs	LMM	FDR (4 rois x 7 metrics)
	Age-effect Fig S6	Age	Morpho. metric	Sex	-	OLS	FDR (3 tracts x 5 metrics)
**FC**	Intra vs. inter Fig 2B	Intra correlation	Inter correlation	-	-	Paired t-test	-
	Age-effect Fig 2D	Age	Inter Correlation	Sex	IDs	LMM	FDR (6 types of connexions)
	Age-effect Table S5	Age	Intra Correlation	Sex	IDs	LMM	FDR (6 types of connexions)
**Dyn**	Intra vs. inter Fig 2B	Intra Similarity	Inter Similarity	-	-	Paired t-test	-
	Age-effect Fig 2E	Age	SpiDyn metric	Sex	IDs	LMM	FDR (4 rois * 20 metrics)
**All**	Coupling Fig 2C, 3BE	Similarity/FC	Similarity/FC	-	-	Spearman correlation	-
	Age-effect in coupling Fig 2F, 3CD	t-values (Similarity or FC)	t-values (Similarity or FC)	-	-	Spearman correlation	-

Brain – spinal cord							
**Morpho**	Age-effect Table S7	Age	GM csa or GM volume	Sex	-	OLS	FDR (10 networks)
	csa vs. brain Fig 4A	Age * GM volume	GM csa	Sex	-	OLS	-
**FC**	Intra vs. inter Table S8	Intra correlation	Inter correlation	-	-	Paired t-test	-
	Age effect Fig 4C	Age	Intra Correlation	Sex	IDs	LMM	FDR (10 networks)
	Age effect Fig 4D	Age	Inter Correlation	Sex	IDs	LMM	FDR (10 networks)
	Intra vs. 0	Intra correlation	0	-	-	t-test	-
	Inter vs. 0	Inter correlation	0	-	-	t-test	-
**Dyn**	Age-effect Fig 4F	Age	SpiDyn metric	Sex	-	LMM	FDR (11 networks)

Table S2. Summary of statistical analyses. For each morphometric (morpho), functional connectivity (FC) or functional dynamics analyses (Dyn). The table lists the type of test, independent variable (X), dependent variable (Y), covariates included, random effects (if applicable), statistical model used, and any multiple comparison corrections applied. IDs: participants; LMM: Linear mixed model; OLS: Ordinary least squares; FDR: False discovery rate; GM: Gray matter

3. The claims about structure–function coupling in the cord and "CNS-wide" organization are interesting, but different types of evidence are currently discussed together. It would help to more clearly separate (a) structure–function coupling within the cord (for example Fig. 3), (b) direct relationships between spinal and brain measures (for example Fig. 4), and (c) parallel age effects seen separately in brain and cord and interpreted as CNS-wide patterns. Making explicit which analyses support each level would strengthen the argument. I would also suggest using terms such as "CNS-wide organization" or "shared CNS-wide aging mechanisms" only when that specific level of evidence is meant.

We thank the reviewer for this helpful comment. The addition of the detailed statistical table (S2) now makes it easier to distinguish which analyses support each level of interpretation, within the table statistical test refers to the related figure. The discussion is structured around three main points

- Spinal cord structural or functional changes
- Spinal cord structural-functional coupling
- CNS-wide organization

To improve clarity, we have added explicit statements throughout the discussion to indicate which analysis supports the discussion.

Line 465: **Using distinct microstructural and functional analyses**, we observed pronounced age-related changes in the dorsal spinal cord, which is primarily responsible for somatosensory processing.
Line 501: **Across both microstructural and functional metrics, the ventral horn showed less pronounced age-related in the ventral horn.**
Line 514: Beyond separate **spinal cord** structural and functional analyses, we examined their interplay and how this coupling evolves with aging.

Line 567: We further examined local time-series dynamics **across brain and spinal cord regions**, using a principal functional gradient approach, **to characterize the intrinsic macroscale organization of CNS activity**[1], independently of aging effects.

4. For the brain, the analyses focus on gray-matter morphometry and resting-state functional connectivity. Could the authors clarify whether diffusion or additional quantitative MRI measures were available and, if so, why the analysis was restricted to GM metrics? A short statement on available modalities and the rationale for focusing on the chosen measures would help the reader understand the scope and limits of the brain–spine comparisons.
Diffusion and other quantitative MRI measures were not acquired for the brain because including them would have roughly doubled the duration of each scanning session. For the brain analyses, we focused on gray-matter tissue to limit the number of regions of interest and maintain reasonable computation time. Importantly, each brain ROI still contains a large number of voxels compared to spinal cord ROIs, ensuring robust signal estimation. In addition, due to differences in organization, partial volume effects are less pronounced in the brain than in the spinal cord, where gray matter regions are small and closely surrounded by white matter. Importantly, structural comparisons between the brain and spinal cord were both limited to the gray-matter and performed using metrics that are most comparable across the two structures: cross-sectional area for the spinal cord and volume for the brain. No microstructural interpretation of spinal cord metrics was made with respect to the brain.

We have now added the following statements in the method section:

- Line 645: **"Thus, microstructural images were specific to the spinal cord, while functional and T1w images were simultaneously acquired for the brain and the spinal cord. Further analyses of structural integrity will consequently be based on T2*w, DWI, and MT images for the spinal cord, while brain analyses will be done based on T1w images."**
- Line 754: The motivation for using these two metrics is that they are the most widely used structural biomarkers for assessing spinal cord or brain atrophy, as they can be derived from various structural contrasts (T1w, T2w, MT), are highly feasible in clinical settings and **were selected as the most comparable metrics between the brain and spinal cord**.

5. For spinal structure, several quantitative measures (T2-based voxel counts, MTR, FA, RD, etc.) are combined into a multivariate feature vector per region and then summarized as morphometric similarity. While this is consistent with previous brain morphometric similarity work, it would help if the authors could (i) clearly summarize which individual metrics show the strongest age effects in dorsal versus ventral regions and (ii) comment on whether the observed pattern of morphometric similarity and its age dependence is robust across individual metrics or is driven mainly by a subset of them. Even a brief figure or table in the Supplementary Material could clarify this.

We thank the reviewer for this comment. We believe that the main text and figures already address parts of this point. Specifically, the morphometric age prediction plots (Fig. 1D) include donut plots showing the relative contribution of each feature to age prediction. In addition, to provide further clarification, the main text also reports the explained variance for each metric, highlighting their respective contributions in both ventral and dorsal horns. These results indicate that the observed pattern of morphometric similarity and its age dependence is robust across multiple metrics rather than being driven by a single measure. This has been specified now in the results section Line 192.
"**In the dorsal horn, WM voxel counts and FA contributed most strongly to age prediction** (WM voxel counts: 83.24% of the explain variance, FA: 73.21%, MTR: 32.93%, RD: 9.34%; GM voxel counts: 0.33%, AD: 0%, MD: 0%). **In the ventral horn, FA and GM voxel counts were the dominant contributors** (FA: 57.88%, GM counts: 57.40%, WM counts: 18.64%, RD: 11.07%, MTR: 0.81%, AD: 0%, MD: 0%). **These results indicate that the age prediction is not driven by a single metric, but instead reflect complementary metric contributions, with distinct dorsal–ventral profiles**."

Minor comments
1. There are a few typos and grammar errors (for example "ans show increase in GM", "GM voxels count"), so a careful proofread would be useful.

We thank the reviewer for this comment. We carefully went through the entire manuscript to correct typographical and grammatical errors.

2. In the Fig. 4 caption, CSA is reported "in mm^3", but CSA is an area measure and should be "in mm^2".
The correction has been done.

3. The QC information is spread across the Methods, Supplementary Figs. S2–S3, and Table S1, which makes it hard to get an overview of data quality. A short QC summary in the main text that brings together FD and tSNR values (and any age effects), Dice for GM and main tracts, and the number of exclusions per modality, plus a statement on whether any QC metrics were tested as covariates, would make the results easier to interpret.
We thank the reviewer for pointing this out. The following paragraph has been added to the main results section (Line 99), and statistical results for FD and tSNR have been removed from the method section.
Data quality was systematically assessed across modalities using visual inspection of all images, as well as quantitative measures including framewise displacement (FD, Fig S2B) and temporal signal-to-noise ratio (tSNR, Fig. S2C) of the functional images (Table S1). Three participants were excluded due to excessive motion (FD > 0.3 mm). When testing for age effects, FD showed a significant increase with age at the brain level ($t(65)=4.75$, $p<0.001$) but not at the spinal cord level ($t(65)=0.34$, $p=0.74$). Functional data quality was further evaluated using tSNR, which showed no significant age-related effects in either the spinal cord (mean ± SD: 22.16 ± 2.0; $p=0.095$) or the brain (58.55 ± 4.9; $p=0.22$). Exclusions based on structural scans were due to modality-specific artifacts (n=15) or incomplete acquisitions (n=1). After quality control, the final sample comprised 67 participants with T2s, T1w, and fMRI imaging. Among these, 65 participants also had MT and 55 DWI data, resulting in a total of 54 participants with complete scan acquisition across all modalities.

4. In the "Time series denoising" and "Functional connectivity analyses" sections, you describe RETROICOR, CSF CompCor, DCT terms, and slice-wise motion regressors, and then compute FC on denoised, z-scored time series. It is not fully clear whether the same nuisance model was used for brain and spinal cord, or whether additional steps were applied for brain FC. A brief clarification of the denoising model for each (and any differences between them) would make the FC and SpiDyn results easier to follow.
We have added this clarification to the manuscript line 822:
"We modelled slice-wise nuisance regressors to account for physiological noise for each participant. **We used the same denoising approach for both brain and spinal cord data, with the only difference being the number of CompCor components applied at each level**"

5. You report tSNR values for spinal cord and brain (for example mean spinal tSNR 22.16 ± 2.0 with no significant age effect; QC section and Fig. S2C), but it is not stated whether tSNR was used as a criterion for excluding participants or runs. Clarifying whether any tSNR thresholds were applied, or confirming that tSNR was only used descriptively, would make the QC procedure more transparent.
We have added the following statement to the main manuscript (Line 802):
tSNR was not used as a criterion for excluding participants; it was reported for descriptive quality control and to compare data quality across participants of different ages.

6. In the Methods section "Spinal dynamic features (SpiDyn)" and the Supplementary figures, you describe extracting time-series features (catch-22, ALFF, standard deviation), excluding those with a dominant single value (f_dom), and later applying PCA with n = 10 components. It would help to summarize, in one place, how many features remain after exclusion and which of these are actually entered into the age analyses.
We thank the reviewer for this helpful suggestion.
We believe that this information is already provided line 906: "In total, we obtained 20 time-series dynamic features (17 from catch-22, mean, standard deviation, ALFF) that include, but are not limited to, distributional properties, autocorrelations and variability of a given time-series."

To make this point even more explicit, we have added an additional clarification elsewhere in the manuscript:

- Line 912: '… normalized across spinal cord parcels to the unit interval using a scaled robust sigmoid function, ensuring comparability across the **20** features with different value ranges'
- Line 258: 'Specifically, **SpiDyn characterized the regional signal fluctuation from 20 features: the signal mean, standard deviation**, amplitude of low fluctuation frequency (ALFF); a commonly used fMRI biomarker of local spinal activity [40–42] , alongside **17** timeseries features derived from the catch-22 toolbox[43] , which quantify the dynamic properties of the spontaneous fMRI signals, such as signal complexity, temporal predictability and variability.'

7.It would also be helpful to cite related qMRI and CNS aging work, in particular Callaghan et al., Neurobiology of Aging 2014, and Labounek et al., Imaging Neuroscience 2025.
We thank the reviewer for this suggestion. Labounek et al., 2025 (ref. 68) was already cited in the manuscript. Regarding Callaghan et al., 2014, since our study does not include quantitative brain MRI but rather volumetric measurement, we have not added this reference, as it is not directly relevant to the scope of the current work

---

Reviewer 3

I co-reviewed this manuscript with one of the reviewers who provided the listed reports. This is part of the Nature Communications initiative to facilitate training in peer review and to provide appropriate recognition for Early Career Researchers who co-review manuscripts

The GitHub repo is clearly linked to the manuscript and lists the main dependencies and versions, with a sensible structure (code, configs, figure notebooks, templates). Scripts and notebooks for preprocessing and Figures 1–4 are provided, but the README notes that some parts (especially supplementary material) are still being uploaded and there is no single step-by-step pipeline. I have not installed or run the code, so I cannot judge full reproducibility, but it seems usable for experienced users and would be easier to use with a more detailed "how to run" guide.

We thank the reviewer for carefully examining the code repository and for this constructive suggestion. We have now completed the upload of all scripts and notebooks related to the supplementary material, which was missing at the time of initial submission. In addition, the README file has been updated to provide a clearer description of the repository structure and a step-by-step guide on how to run the main preprocessing and analysis pipelines. These additions are intended to facilitate reproducibility and make the code easier to use for readers who wish to explore or adapt the workflow.
https://github.com/CarolineLndl/Landelle_spinebrain_aging

Reviewer 4

In this paper, Landelle et al. investigates the structural and functional changes along the whole neuroaxis in a cohort across the lifespan. The paper and data are definitely of high interest and timely. The figures are beautiful. And I can see that, authors did a lot of work to acquire and analyze the data with novel methods. I do have major and minor comments.

Major: My major comments relate to interpretability, clarity of age-related findings, and methodological transparency.
My main concern: The authors have done rigorous work. However, the main take-away are not clear. I would love to see clear and understandable age effects on each metric as shown in Supplementary Figure S5. I further specified this concern below.
Results:

I would like to see a histogram of Age across people. It is unclear how the Age is distributed across the sample.
Also, please provide a missingness analysis, are the subjects who have missing data, older people? If this is the case, is not this a confound? I believe the Supp. Fig S4 is incorrect (B and C are identical), the N is inconsistent.
We thank the reviewer for this suggestion. To address the comment, we have added a histogram of participant ages to complement the age distribution shown in Supplementary Figure S2A.

A I Age

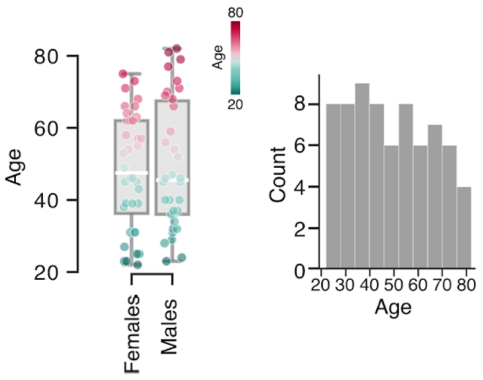

We also summarized the age of participants with and without missing data in the following table. The distributions appear comparable, suggesting that missing data is unlikely to introduce an age-related confound:

	N	Mean age	SD	Median	Range
**Missing data**	15	51.1	15.4	46	23-77
**All data**	67	48.83	16.94	46	22-82

Finally, we carefully checked Supplementary Figure S4 and the reported sample sizes. The labelling B and C has been corrected. We confirm that the Ns reported were correct: 65 participants for MT, 67 for T2s, and 55 for FA.

2. Although very interesting, the paper is very dense and hard to follow. I appreciate the rigor and novelty in the analysis, the main premise of the paper is to demonstrate the effects of aging across CNS. However, this premise gets very hard to clearly see with the metrics like “morphometric similarity” and “SpiDyn”. What does morphometric similarity mean, how can one interpret it? What do SpiDyn features mean and how can we interpret the similarity between them. It is abstraction of an abstract feature. I really would like to see Supplementary Figure 5 in the main text and the more complex metrics can be introduced more in detail later (and some of them can be moved to supplement).
We thank the reviewer for this thoughtful and important comment. We agree that the manuscript is dense and that the use of multiple measures, such as morphometric similarity, SpiDyn and SpiFC can make the central message harder to follow if their respective motivations and interpretations are not made sufficiently explicit. In essence, both the SpiDyn similarity matrix and the Morphometric similarity matrix are analogous to the functional connectivity matrix: they summarize patterns of similarity or connectivity across regions, highlighting which regions are more alike in their structural or functional properties. To address this, we have revised the manuscript in several ways. First, we now provide a clearer conceptual explanation of morphometric similarity and SpiDyn at their first introduction in the main text.

- Line 150 : “**To provide a multivariate representation of spinal cord microstructure, we combined all microstructural features into a single regional profile, allowing us to capture shared microstructural patterns between the different regions that are not evident when individual metrics are examined separately. Morphometric similarity between two spinal cord regions was then defined as the Pearson correlation between their microstructural features, providing an index of how similar regions are in their overall microstructural organization. High morphometric similarity therefore indicates that two regions share comparable microstructural organization across multiple MRI features. Using this approach, we found that** regions belonging to the same spinal cord

segment exhibited greater similarity than those from other cervical segments (Fig. 1C, paired two-tailed t-test; $t(53)= 22.86$, $p<0.001$)."

- Line 253 : **To capture the intra-regional dynamical signature of spinal cord function beyond inter-regional connectivity, we next analyzed the** dynamic profiles of spinal cord activity (SpiDyn) by analyzing the spontaneous temporal dynamic of timeseries fluctuations within **each of the 28 regions. Specifically, SpiDyn characterize the regional signal fluctuation from 20 features:** the signal mean, standard-deviation, amplitude of low fluctuation frequency (ALFF); a commonly used fMRI biomarker of local spinal activity 40–42 , alongside 17 timeseries features derived from the catch-22 toolbox43 , which quantify the dynamic properties of the spontaneous fMRI signals, such as signal complexity, temporal predictability and variability**. To summarize the similarity of dynamic signature across regions, we constructed a SpiDyn similarity matrix by computing pairwise correlations between the 20-dimensional feature vectors of each spinal region (Fig. 2b). This provides an index of how alike the dynamic profiles are across the spinal cord, analogous to the morphometric similarity approach applied to structural features.**

Supplementary Figure 6 (previously S5) represents an additional analysis conducted on tract-specific regions rather than the quadrant-based parcellation used in the main analyses. Therefore, it is not directly comparable to the main text figures. In contrast, Figure 1D summarizes the age effects across all four quadrants and all microstructural features in a single view, which we believe provides a more intuitive and comprehensive overview of which features exhibit age-related changes. This rationale has now been clarified in the main figure caption, and we add a new supplementary figure (S5) that represent that age effect for each metric and each quadrant.

- Improved figure caption (D): D. The heatmap represents t-values reflecting the age-related effect on each microstructural feature (blue indicates a decrease with age, gray indicates an increase). The mean absolute **age** effect across features is illustrated on an axial view of the spinal cord for each quadrant, **providing a concise overview of how each region is affected across metrics**.
- Clarity in the results text Line 168: **Fig. 1D provides a comprehensive overview of age effects across all features and quadrants**, showing significant age-related decreases for MTR, WM voxels count, GM voxels count and FA, alongside an increase in RD.

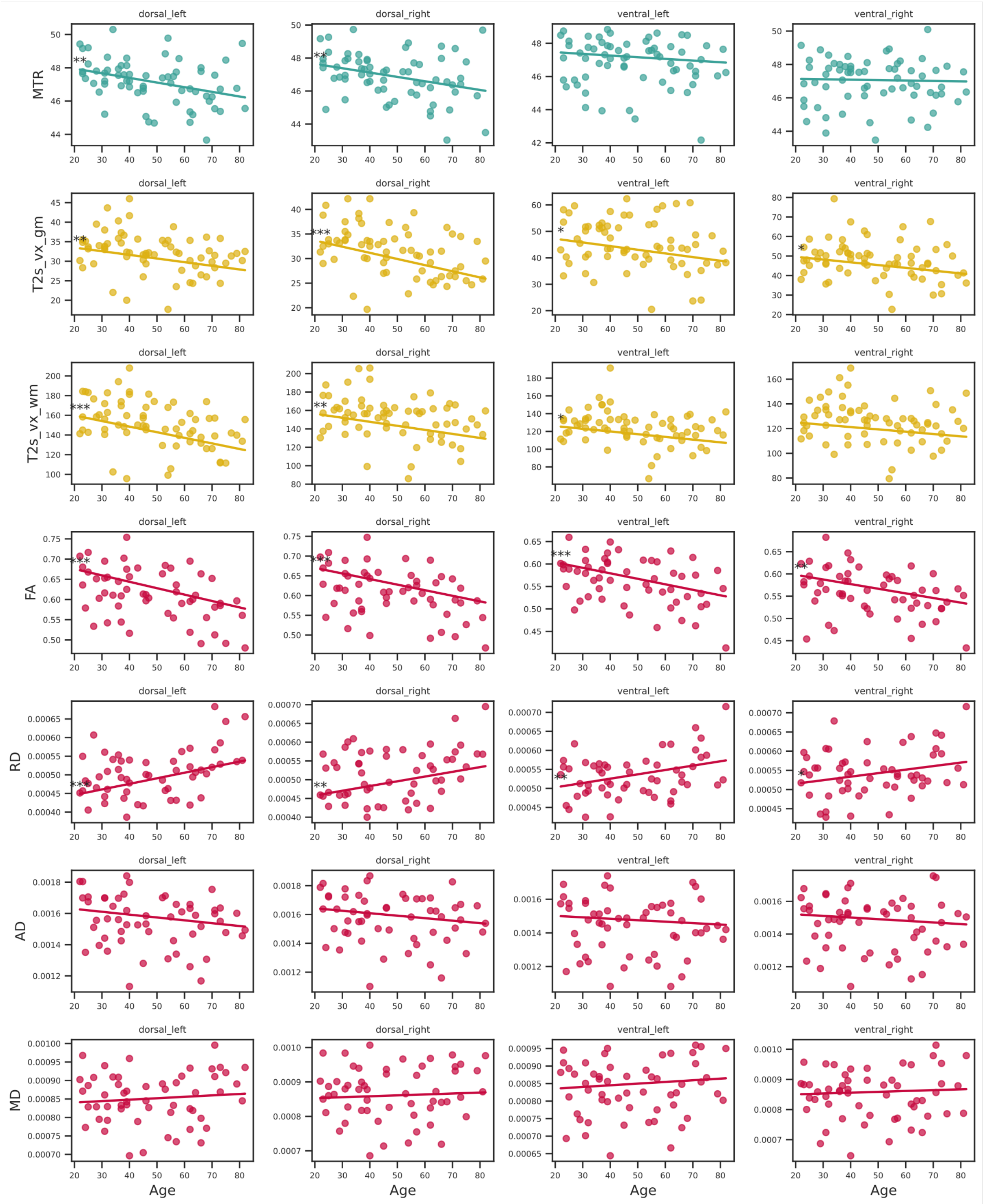

3. Similar to this, SpiDyn, is an interesting and novel concept, however, it makes it harder to follow the paper and reduces interpretability. Have those metrics been used previously (in brain literature) – if yes, how have they been interpreted? I am also not sure about the "Dyn" in the name. ALFF has not been considered a "dynamic" resting-state fMRI measure. Additionally, I can see that the metrics also include mean and SD. How is the mean showing the dynamic properties of that ROI? Since the SpiDyn is highly correlated with spiFC, I do not think it adds that much to the manuscript (particularly considering the interpretability). These should be moved to supplement and age effects Age Vs spiFC can be shown clearly.

We thank the reviewer for this thoughtful comment. We agree that SpiDyn is a relatively novel approach and can be harder to interpret than traditional FC metrics, but some of its components, such as ALFF or autocorrelation, are widely used in clinical studies. The rationale behind SpiDyn is to extend the characterization of spontaneous signal fluctuations using a broader set of timeseries

features. This approach is inspired by analogous approach in brain imaging (fMRI: Shafiei et al., 2020, Bryant et al. 2024; MEG: Shafiei et al., 2023), where features capturing variability, autocorrelation, and complexity (e.g., Catch-22 features, ALFF, SD, mean) are used to describe the dynamical properties of resting-state fMRI signals. Including SpiDyn complements SpiFC by capturing intra-regional temporal characteristics (*i.e.*, timeserie properties for a given region) that are not reflected in inter-regional correlations (*i.e.*, statistical dependence between the timeseries of two regions). Importantly, SpiDyn allows direct comparisons between local microstructural properties and local functional dynamics, reflecting heterogeneous regional activity, while spiFC provides information at the network level by linking structural organization with functional connectivity across regions or networks.

While SpiDyn and SpiFC values are correlated, they are not redundant, as SpiDyn adds insight into the heterogeneity of regional dynamics and allows us to quantify the similarity of these dynamic signatures across regions, analogous to how morphometric similarity summarizes structural profiles. This can be particularly useful because it yields to whole-spinal cord or whole-brain maps of regional dynamic profiles, enabling precise localization of functional alterations or disruptions. It also open the door to questions that cannot be addressed by pairwise functional connectivity alone, such as how local signal dynamics within a specific region changes in the context of lesions, focal pathology, or localized microstructural damage. For instance, in our study, we observed that dorso–ventral connectivity changes with aging, while spontaneous activity changes were predominantly located in the dorsal horn. This suggests that age-related connectivity alterations may be primarily driven by changes in dorsal horn activity, an insight that could not be inferred from functional connectivity analyses alone.

Importantly, the standard deviation (SD) is a simple yet informative measure of dynamics, as it captures the amplitude of temporal fluctuations. The mean does not directly reflect dynamics but provides the baseline signal level, contributing to the overall regional profile.

To improve clarity and interpretability, we have added a more explicit description of SpiDyn when it is introduced in the paper the first time:

- Line 253: "**To capture the intra-regional dynamical signature of spinal cord function beyond inter-regional connectivity**, we next analyzed the dynamic profiles of spinal cord activity (SpiDyn) by analyzing the **spontaneous temporal dynamic** of timeseries fluctuations within each of the 28 regions. **Specifically, SpiDyn characterizes the regional signal fluctuation from 20 features**: …"

Reference:

Shafiei G, Markello RD, Vos de Wael R, Bernhardt BC, Fulcher BD, Misic B. Topographic gradients of intrinsic dynamics across neocortex. Elife. 2020 Dec 17;9:e62116. doi: 10.7554/eLife.62116. PMID: 33331819; PMCID: PMC7771969.

Bryant AG, Aquino K, Parkes L, Fornito A, Fulcher BD. Extracting interpretable signatures of whole-brain dynamics through systematic comparison. PLoS Comput Biol. 2024 Dec 23;20(12):e1012692. doi: 10.1371/journal.pcbi.1012692. PMID: 39715231; PMCID: PMC11706466.

Shafiei G, Fulcher BD, Voytek B, Satterthwaite TD, Baillet S, Misic B. Neurophysiological signatures of cortical micro-architecture. Nat Commun. 2023 Sep 26;14(1):6000. doi: 10.1038/s41467-023-41689-6. PMID: 37752115; PMCID: PMC10522715.

4. Although I find the figures aesthetically very appealing, the coloring is confusing. Please make sure it is consistent between the figures and always indicates the same thing. For instance, in Figure 1D, the t value map has the same color scheme for age in Figure 4A (same for supplementary figures).
Figure 1D has been updated with a color map from blue to gray to avoid confusion with color attributed each microstructural metrics.

5. Please make sure Figure legends are informative and information rich in a way that what the figures show can be understood without going back to Methods. Especially for some figures, I found it hard to understand what exactly each plot shows. For instance, it should be specified in the legend that figure 1C shows group-averaged similarity matrix. Sometimes the FDR corrected values are

depicted as (*); see Figure 2E as an example, and sometimes not (Figure 4D). Please make sure these are consistent across Figures, and the same symbol always signifies the same thing/value.
We thank the reviewer for this important suggestion. We agree that figure legends must be sufficiently detailed. We have therefore carefully revised all figure captions to make them more explicit. Fig 1 caption now specifies that C shows the group-averaged morphometric similarity matrix.

We have standardized the notation used to indicate statistical significance across all figures. An asterisk * now consistently denotes results significant at $p < 0.05$ before multiple-comparison correction, while a circled asterisk (*) denotes results surviving FDR correction. This convention is now applied uniformly across all figures.

6. Following up on that, it is unclear what ROI has been used for which analysis? For spiFC, was it gray matter only or quadrant? If quadrant, Figure X is misleading. If gray matter for spiFC and whole quadrant for spiDyn, why is the discrepancy?
We thank the reviewer for this comment. For both SpiFc and SpiDyn, the same ROIs were used and included both gray and white matter. To improve clarity, we have updated the illustration in Figure 2D so that it accurately reflects the quadrants used and avoids any potential confusion.

7. Which analysis was done in template space, which analysis was done native space? It is very hard to follow. Please make it clearer.
We thank the reviewer for pointing out that the distinction between native-space and template-space analyses was not sufficiently clear. Briefly, structural and microstructural analyses (including voxel-based tissue counts, cross-sectional area, and quantitative MRI metrics) were performed in native space to preserve the highest spatial resolution and avoid interpolation errors that could bias voxel counts or CSA estimates. In contrast, functional analyses (SpiFC and SpiDyn) were performed in template space. This choice was motivated by the lower spatial resolution of fMRI compared to structural imaging and the greater inter-participant variability introduced by image deformation during motion and distortion correction. Registering functional data to template space ensures consistent alignment across participants.

We have added a concise statement in the Methods to specify that microstructural and structural analyses were conducted in native space, while functional MRI analyses were performed in template space, as already described in the fMRI processing section

- Line 690: '**Microstructural analyses were conducted in the participant's native space.**'
- Line 752: '**Structural metrics were extracted in the participant's native space.'**

8. Related to that, some of the scatter plots such as Figure 3 show multiple points for each subject. What exactly do these shows? How many points from each subject are there? How has this been this accounted in analysis (a mixed-effects models needs to be used not to artificially inflate the p-values)?
We thank the reviewer for raising this point. The scatter plots in Figure 3 do not show multiple data points per participant. Instead, each dot represents a pairwise combination of spinal regions, not individual participants. In panels B and E, coupling was performed on participant-averaged matrices and each point corresponds to one ROI-ROI pair, where the x-axis values represent functional connectivity or SpiDyn and y-axis values morphometric similarity. In panels C-F, each point also corresponds to one ROI-ROI pair, where the values plotted are t-statistics from age-effect models (C: Morphometric similarity vs SpiFC and F: Morphometric similarity and SpiDyn). Therefore, these scatter plots do not contain repeated observations from individual participants, and no participant contributes multiple independent data points to the correlation analyses. As a result, there is no inflation of degrees of freedom, and mixed-effects models are not required for these specific correlations.

To avoid further confusion, we have revised the figure captions to explicitly state that each dot represents a pairwise combination of spinal regions.
B, E. **Coupling between group-average morphometric similarity and SpiFC (B) or SpiDyn (E) was assessed using Spearman correlations across pairwise combinations of spinal regions (24x24). Each dot represents one region-region pair, and r denotes Spearman's correlation coefficient**. We found a strong positive correlation between the two matrices. C, F. **To determine whether spinal region pairs showing age-related changes in morphometric similarity also exhibit corresponding changes in SpiFC or SpiDyn, we computed correlations between t-values from age-effect models. The scatter plot shows the correlation between age-related**

effects on morphometric similarity and SpiDC or SpiFC across spinal region pairs (r denotes the Spearman's correlation coefficient).

The method section was also updated for clarity (line 946):
Structural and functional coupling was estimated across the spinal cord using participant-averaged matrices. For each matrix (microstructural, SpiFC, SpiDyn), individual matrices were first stacked and averaged across participants to produce group-level matrices. From these matrices, only the upper-triangle edges were extracted (n_nodes x (n_nodes-1)/2) and Spearman correlations were computed between the corresponding edges, yielding one coupling coefficient for each matrix pair. Coupling analyses involving morphometric similarity were performed only in participants with complete structural and functional acquisitions (n=54), whereas coupling between functional metrics (SpiFC-SpiDyn) used the 67 participants. Next, the age-related change **was investigated using** OLS regression models for each **pairwise** region **combination,** with coupling strength as the dependent variable, age as a fixed effect and sex as a covariate**. Finally, to determine whether spinal region-region connection or similarities exhibit corresponding age-related changes across microstructure, SpiFC, and SpiDyn, we computed Spearman correlations between pairwise regional t-values derived from the OLS age-effect models. In these analyses, each data point represents a pairwise region combination at the group level (i.e. the average or age-related t-value).**

9. Intra vs inter network results are interesting, but could not it be mainly due to the spatial proximity? How many EPI slices were within one segment? Was the analysis done in native space or template space?

We acknowledge that spatial proximity along the spinal cord could in principle contribute to higher similarity or connectivity between regions within the same or neighboring segments. Cervical segments are approximately 9-11 mm long (Cadotte et al., 2015), and in our dataset, each segment spans roughly two axial slices (functional slice thickness: 4 mm). Functional analyses (SpiFC and SpiDyn) were conducted in template space to ensure consistent alignment of spinal segments and quadrants across participants. Thus, regions within the same segment are not confined to a single imaging slice, and regions from adjacent segments are separated by comparable physical distances. No spatial smoothing was applied, minimizing artificially induced correlations between nearby regions.

If spatial proximity alone were driving the functional connectivity/similarity, inter-segment functional connectivity/similarity values would be expected to be uniform across all distances and not significantly different from 0. Instead, we observe a progressive decrease in similarity or connectivity with increasing segment distance, indicating that the effects reflect a functional gradient. Supporting this interpretation, in a previous study (Landelle et al. 2023) with the same acquisition protocol, we demonstrated that the decrease in connectivity with segment distance differed between control participants and Parkinson's patients at distinct disease stages, confirming that these effects are biologically meaningful rather than acquisition driven. In the present study, inter-segmental connectivity increased with aging, while intra-segmental connectivity did not. Moreover, selective age-related effects were observed in specific connection types, particularly dorso-ventral and cross-horn connections, further arguing against a purely distance-driven explanation.

10. Please clarify spinal cord GM volume calculation. Native space, template space? Is it normalized?
No gray matter volume was calculated for the spinal cord. Instead, we extracted the number of gray matter voxels and computed the gray matter cross-sectional area (CSA) for each segment. For microstructural analyses, all data were kept in each participant's native space and were not normalized. We clarify the sentence about GM count:
Line 696: First, we calculated the number of voxels within the GM or the WM in the T2*w image (voxel counts) **from** the automatic segmentation of the image in individual **native** space.

And added the two following sentences:

- Line 690: **Microstructural analyses were conducted in the participant's native space.**
- Line 752: **Structural metrics were extracted in the participant's native space.**

Over-statements in Discussion:

11. The authors conclude that the findings “lay the groundwork for early biomarkers of sensorimotor decline”. This oversteps the evidence – the study is cross-sectional in healthy adults and did not track actual sensorimotor decline or test predictive power for future impairment. No behavioral measures of sensorimotor performance were included, so there is no direct link between the MRI metrics and functional decline. Calling these measures “early biomarkers” is speculative. To support such a claim, longitudinal data showing that these imaging measures predict sensorimotor decline (or at least correlate with current performance deficits) would be needed.
We agree with the reviewer’s concern and have revised the text to tone down the statement.

Line 31: “Together, our findings provide a systems-level view of age-related alterations in CNS, **offering a foundation for future studies investigating potential imaging markers of sensorimotor dysfunction**.”

12. The authors claim to provide the “first evidence that dedifferentiation extends across the CNS”, referring to the age-related increase in inter-network functional connectivity in both brain and spinal cord. It is true the data show a trend toward less network-specific connectivity with age (i.e. more between-network connectivity, interpreted as dedifferentiation) in the spinal cord, mirroring a known brain aging effect. However, this interpretation may be somewhat overstated. First, the dedifferentiation effect size appears modest. For example, the reported increase in inter-network connectivity with age, though significant, is not very large in magnitude (the authors describe it as a “weak but significant” correlation in the spinal cord). Additionally, not all aspects of network organization followed the dedifferentiation pattern uniformly. In the brain, some networks (visual, dorsal attention, cerebellar, etc.) actually showed increased intra-network connectivity with age, which is opposite to a loss of specialization. This nuanced result suggests the story is more complex than a simple global dedifferentiation.
We thank the reviewer for this important comment. We would like to clarify that the description of “weak but significant” specifically referred to the SpiFC-SpiDyn coupling (line 304), not the inter-network functional connectivity itself. In fact, the age-related increase in inter-network connectivity was highly significant, as further confirmed by our new bootstrap analyses, which showed that the observed effects were consistently greater than zero across 1000 resamples for all networks (Table S10). Moreover, we believe that the observed increases in intra-network connectivity in some brain regions do not contradict the general trend of reduced network specificity. These increases could reflect enhanced integrative processes or compensatory mechanisms. However, we agree that the overall story is more complex and falls beyond the main focus of our study. To simplify the discussion and avoid over-interpretation, we have replaced the term “dedifferentiation” with the more neutral “network segregation” throughout the manuscript.

- Abstract: Extending analyses to the brain uncovered convergent CNS-wide aging mechanisms, including gray matter loss, **decreased functional segregation**
- Line 418: We found a significant increase in the spinal cord and brain inter-network FC with aging, suggesting a shift toward less specific FC patterns, **reflecting less segregated networks** [46] at each CNS level
- Line 562: Our results suggest that a similar **reduction in network segregation** extends across the CNS.

13. The authors observed that the left dorsal quadrant of the cervical cord (which corresponds to afferents from the non-dominant arm in this all right-handed sample) exhibited the most pronounced aging effects, and they speculate that “reduced limb use may accelerate aging in its corresponding spinal territory”. This is an interesting hypothesis, but it is presented rather strongly despite being unsupported by direct evidence in the study. No data on limb use frequency, physical activity, or motor behavioral decline in the left vs. right side were collected. Thus, attributing the greater left-side changes to disuse is speculative. The asymmetry could have other explanations.
We agree that attributing the greater age-related effects in the left dorsal quadrant to reduced limb use is speculative, as we did not collect behavioral measures of limb activity or motor function. We have revised the manuscript to present this observation more cautiously, highlighting the lateralized effect without over-interpreting its cause.
Line 496: **While this study did not allow us to determine the reason for this lateralization, it may reflect differences in limb use, horn specialization, or other factors, highlighting a potential target for future studies and somatosensory rehabilitation**.

Minor:
14. There are some typos across the manuscript, please correct them.
We thank the reviewer for pointing this out. We have carefully proofread the entire manuscript and corrected all typos and minor language errors to improve clarity and readability.

15. References are unorganized. Sometimes they are numbered and sometimes they are in-text APA style (with authors surname and year).
We thank the reviewer for this comment. The references have now been standardized and are consistently formatted throughout the manuscript.

16. Why are the small ROI to ROI FC matrices (RV-RV etc) not symmetric?
The apparent asymmetry in the small ROI-to-ROI FC matrices was due to a graphical error, which has now been corrected in Figures 1 and 2.

17. I understand that one cannot try every method. But what if the age effects were not linear? Did authors explore that?
We retained the linear modeling approach for clarity and interpretability, while acknowledging that future studies with larger samples or longitudinal designs could more fully characterize potential nonlinear trajectories of aging.

Line 601: "Longitudinal studies will be essential to disentangle progressive decline from compensatory reorganization **and to explore potential nonlinear trajectories of aging**."

Reviewer #4 (Remarks on code availability):

Code is there, seems well organized and commented. I understand authors may not want to share data due to ongoing analysis. Are there any example derivatives files (from one subject) which can be added to follow the code?

We thank the reviewer for the suggestion. We have now provided examples of the raw data and derivative files from a single participant. https://doi.org/10.18112/openneuro.ds007313.v1.0.0
In addition, we now provide a complete Source Data zip folder containing the data underlying all figures.
